# Author Correction: IL-17D-induced inhibition of DDX5 expression in keratinocytes amplifies IL-36R-mediated skin inflammation

**DOI:** 10.1038/s41590-026-02636-x

**Published:** 2026-08-17

**Authors:** Xinhui Ni, Yi Xu, Wang Wang, Baida Kong, Jian Ouyang, Jiwei Chen, Man Yan, Yawei Wu, Qi Chen, Xinxin Wang, Hongquan Li, Xiaoguang Gao, Hongquan Guo, Lian Cui, Zeyu Chen, Yuling Shi, Ronghui Zhu, Wei Li, Tieliu Shi, Lin-Fa Wang, Jinling Huang, Chen Dong, Yuping Lai

**Affiliations:** 1 Shanghai Frontiers Science Center of Genome Editing and Cell Therapy, Shanghai Key Laboratory of Regulatory Biology, School of Life Sciences, East China Normal University, Shanghai, China; 2 Department of Dermatology, Shanghai Tenth People’s Hospital, School of Medicine, Tongji University, Shanghai, China; 3 Shanghai Skin Disease Hospital, School of Medicine, Tongji University, Shanghai, China; 4 Department of Dermatology, Huashan Hospital, Fudan University, Shanghai, China; 5 Emerging Infectious Diseases Program, Duke–NUS Graduate Medical School, Singapore, Singapore; 6 Singhealth Duke–NUS Global Health Institute, Singapore, Singapore; 7 Institute for Immunology and School of Medicine, Tsinghua University, Beijing, China; 8 Shanghai Immune Therapy Institute, Shanghai Jiaotong University School of Medicine-affiliated Renji Hospital, Shanghai, China

**Keywords:** Chronic inflammation, Inflammatory diseases

Correction to: *Nature Immunology* https://doi.org/10.1038/s41590-022-01339-3, published online 21 October 2022.

In the version of this article initially published, in Fig. 2, the representative flow cytometry panel in Fig. 2h and the GAPDH loading control in Fig. 2b were inadvertently duplicated. In addition, an incorrect total cell number on the *y*-axis of Fig. 2h was entered during figure assembly. In Fig. 3, the p-AKT panel in Fig. 3h was inadvertently duplicated from the p-p38 panel in Fig. 3g, and the SMAD2/3 panel in Fig. 3i was inadvertently duplicated from the AKT panel in Fig. 3g. In Extended Data Fig. 4a, an incorrect GAPDH loading control was included during figure preparation. Part of the Source Data for Extended Data Fig. 3a was duplicated during Source Data integration due to a copy-and-paste error. The figures and Source Data are now updated in the HTML and PDF versions of the article. For comparison, the original and revised figure panels are available as Supplementary Information alongside this amendment.

## Supplementary information

Supplementary Information Original and revised Figs. 2b, 2h, 3h, 3i, Extended Data Fig. 4a

