# Supplementary material for: IL-17D-induced inhibition of DDX5 expression in keratinocytes amplifies IL-36R-mediated skin inflammation
Source: Nat Immunol. 2022 Oct 21;23(11):1577–87. doi: 10.1038/s41590-022-01339-3 (PMC9663298; doi:10.1038/s41590-022-01339-3)
Supplement: Supplementary file 1 — Supplementary Figs. 1 and 2 and Tables 1–4. [file 41590_2022_1339_MOESM1_ESM.pdf]

nature immunology

Article https://doi.org/10.1038/s41590-022-01339-3

# IL-17D-induced inhibition of DDX5 expression in keratinocytes amplifies IL-36R-mediated skin inflammation

In the format provided by the authors and unedited

# Supplementary information

## Supplementary gating strategy

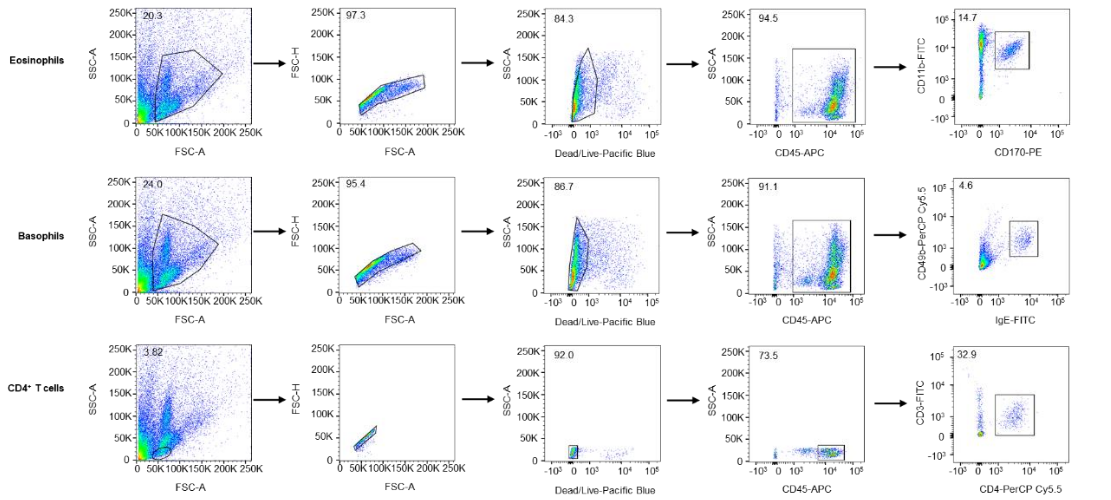


**Gating of eosinophils, basophils and CD4⁺ T cells in lesional skin from AD-like mice.**

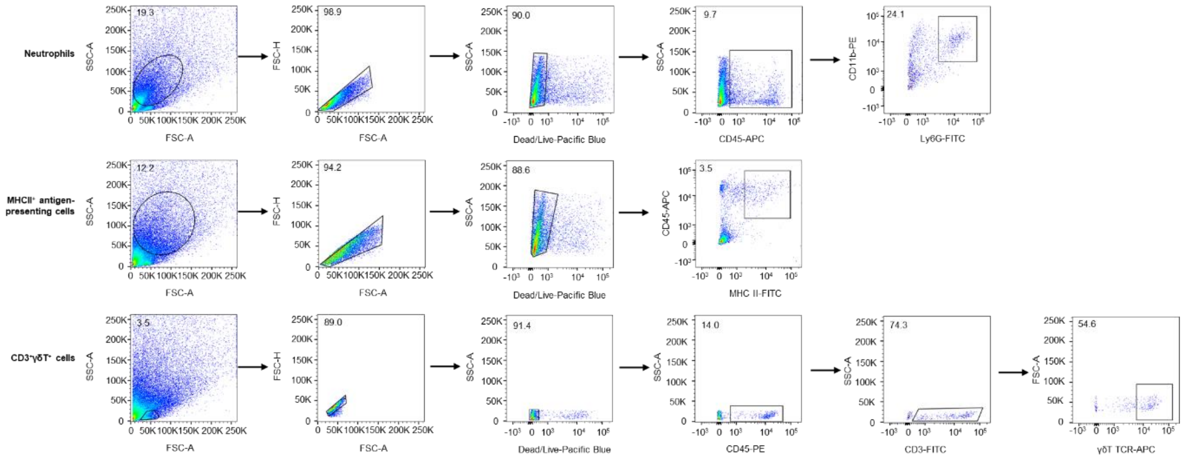


**Gating of neutrophils, MHCII⁺ APCs and γδT cells in lesional skin from psoriasis-like mice.**

**Table 1. The information of healthy controls and patients with AD, psoriasis, BCC and SCC**

| Sample ID | | Age | Sex | Comorbidities |
|---|---|---|---|---|
| Healthy control | 1 | 29 | female | no |
| | 2 | 63 | female | no |
| | 3 | 37 | female | no |
| | 4 | 27 | female | no |
| | 5 | 26 | male | no |
| | 6 | 63 | male | no |
| | 7 | 67 | male | no |
| | 8 | 64 | male | no |
| AD | 1 | 14 | female | no |
| | 2 | 41 | female | no |
| | 3 | 57 | female | no |
| | 4 | 54 | female | no |
| | 5 | 59 | male | no |
| | 6 | 34 | male | no |
| | 7 | 39 | male | no |
| Psoriasis | 1 | 73 | female | no |
| | 2 | 42 | female | no |
| | 3 | 68 | female | no |
| | 4 | 30 | female | no |
| | 5 | 67 | female | no |
| | 6 | 43 | female | no |
| | 7 | 59 | male | no |
| | 8 | 74 | male | no |
| | 9 | 52 | male | no |
| | 10 | 23 | male | no |
| Basal cell | 1 | 56 | male | no |

| | | | | |
|---|---|---|---|---|
| carcinoma | 2 | 73 | female | no |
| (BCC) | 3 | 79 | female | no |
| Squamous cell | 1 | 64 | male | no |
| carcinoma | 2 | 61 | male | no |
| (SCC) | 3 | 67 | female | no |

## Table 2. Guide RNAs and siRNAs

| Name | Sequence (5′→ 3′) | Purpose |
|---|---|---|
| Cas9-*DDX5*-gRNA | CACCGTATTCGAGTGACCGAGACCG<br>AAACCGGTCTCGGTCACTCGAATAC | Deletion |
| Cas9-*SF2*-gRNA | CACCGAACGATTGCCGCATCTACGT<br>AAACACGTAGATGCGGCAATCGTTC | Deletion |
| *siDDX5* | CCCGAAGCCAGUUCUAAAUTT<br>AUUUAGAACUGGCUUCGGGTT | Silencing |
| *siSF2* | GCUAUGAUUACGAUGGGUATT<br>UACCCAUCGUAAUCAUAGCTT | Silencing |
| *siIL36R* | GCAGGAGGCUGAUUGUCAUTT<br>AUGACAAUCAGCCUCCUGCTT | Silencing |
| *siCD93* | GUGUGCAAGUUCAGCUUCATT<br>UGAAGCUGAACUUGCACACTT | Silencing |
| *siDDX1* | GGAGUUAGCUGAACAAACUTT<br>AGUUUGUUCAGCUAACUCCTT | Silencing |
| *siDDX3X* | GCCUCAGAUUCGUAGAAUATT<br>UAUUCUACGAAUCUGAGGCTT | Silencing |
| *siDDX17* | CCUUCCAUCAUGCUAACUUTT<br>AAGUUAGCAUGAUGGAAGGTT | Silencing |
| *siDDX23* | GGAAACAGUUCCAAGACUUTT<br>AAGUCUUGGAACUGUUUCCTT | Silencing |
| *siDDX39* | GCAGCAGUACUACGUCAAATT<br>UUUGACGUAGUACUGCUGCTT | Silencing |
| *siDDX41* | CCAUUCAGAUCCAGGGCAUTT<br>AUGCCCUGGAUCUGAAUGGTT | Silencing |
| *siDDX42* | GCAGUGAUUGUGUGUCCUATT<br>UAGGACACACAAUCACUGCTT | Silencing |
| *siDDX46* | GCAACAAGUGAUUGUGAUUTT<br>AAUCACAAUCACUUGUUGCTT | Silencing |
| *siDDX48* | GCAAUCCAGCAACGAGCAATT<br>UUGCUCGUUGCUGGAUUGCTT | Silencing |

## Table 3. Primers for constructing plasmids or RT-PCR

| Name | Sequence (5′→ 3′) | Purpose |
|---|---|---|
| *mIl23a* | CCCGTATCCAGTGTGAAGATG<br>GGCTCCCCTTTGAAGATGTC | RT-PCR |
| *mIl17a* | GCTCCAGAAGGCCCTCAGA<br>CTTTCCCTCCGCATTGACA | RT-PCR |
| *mDdx5* | TGTGATTGCAAGGCAGAACTTT<br>TGGCCAGCCCTGAGCTT | RT-PCR |
| *mIl17d* | GAGGAGGACTTCCGCTTTCG<br>TGGTGATGTAGTGTTCGGCG | RT-PCR |
| *mIl36α* | GCAGACACATTCCTATTCAATCA<br>TGCTGCTCTTAGTTCTTTCTCCTTA | RT-PCR |
| *mIl36β* | ATGATGGCTTTCCCTCCAC<br>ATTCGGTTCCCACATTTGAA | RT-PCR |
| *mIl36γ* | GGACACCCTACTTTGCTGCTA<br>AACAGGAATGGCTTCATTGG | RT-PCR |
| *mCcl20* | GACTGTTGCCTCTCGT<br>TGACTCTTAGGCTGAGGA | RT-PCR |
| *mCxcl1* | CAGACCATGGCTGGGATTCA<br>GACTTCGGTTTGGGTGCAGT | RT-PCR |
| *mCxcl2* | GGCGGTCAAAAAGTTTGCCTT<br>ATGATTTTCTGAACCAGGGGG | RT-PCR |
| *mCxcl11* | AAACGACAAAGGTGCCTGGA<br>CTGCATTATGAGGCGAGCTT | RT-PCR |
| *mS100a9* | ATACTCTAGGAAGGAAGGACACC<br>TCCATGATGTCATTTATGAGGGC | RT-PCR |
| *mIl4* | ATCTACAGGACCCAGAGGATATTGC<br>CTGATGTGAGAAAGGAAAATGAGTCC | RT-PCR |
| *mIl13* | CGCCATGCACGGAGATG<br>CGAGCTCACTCTCTGTGGTGTT | RT-PCR |
| *mTslp* | AGGCTACCCTGAAACTGAG<br>GGAGATTGCATGAAGGAATACC | RT-PCR |
| *mCcl11* | CTCCACAGCGCTTCTATTCCT<br>TAAAGCAGCAGGAAGTTGGGA | RT-PCR |
| *mCcl17* | CAATGTAGGCCGAGAGTGCT<br>TGCCCTGGACAGTCAGAAAC | RT-PCR |
| *mCcl22* | TCTGGACCTCAAAATCCTGCC<br>GTCATCAGGTCCTCCTCCCTA | RT-PCR |
| *CDC5L* | AATCTCGTGAACATCTCCGTTTAGG<br>CATCCACATCAGCAGCATCTTC | RT-PCR |
| *PRPF18* | TGTGGCTACAAGATACAGCCAA<br>CGGCTCTCCTCTTTCTCTCAG | RT-PCR |

| Name | Sequence (5′→ 3′) | Purpose |
|---|---|---|
| *DHX15* | CACATTTAGAACGAACGGGGC<br>AAGCACCCATTCAGGCTTGT | RT-PCR |
| *HNRNPA1* | AGGGTTATGGAAACCAGGGC<br>TTCCACTACCACCGCCAAAG | RT-PCR |
| *SRSF1* | GCGTGAGGCAGGTGATGTAT<br>GTAGGCAGTTTCTCCCTCGT | RT-PCR |
| *mGapdh* | CTTAGCCCCCCTGGCCAAG<br>TGGTCATGAGCCCTTCCACA | RT-PCR |
| *mIl36R-368* | GGGAGACACTTCACCATTAG<br>ATGATGTAGGCTGCGGACAC | DNA-PAGE |
| *m18S* | ATCAGATACCGTCGTAGTTC<br>CCAGAGTCTCGTTCGTTAT | PCR |
| *hGAPDH* | TCCACTGGCGTCTTCACC<br>GGCAGAGATGATGACCCTTTT | RT-PCR |
| *hDDX5* | GCCATGTCGGGTTATTCG<br>GGTTTCCAAACTTCTTTCCAGA | RT-PCR |
| *hIL36α* | CATCTACCTGGGCCTGAATG<br>GGGTTGGTTGTACAAATCCATTA | RT-PCR |
| *hIL-36β* | GACCAATGGGGAATAGGAGTG<br>CCGCATGGATGAGAAATCTT | RT-PCR |
| *hIL36γ* | TGAGAGGCACTCCAGGAGAC<br>GTCCCAGTAATAGGTTTACACATTGA | RT-PCR |
| *hIL17D* | TGGGCCTACAGAATCTCCTACGACC<br>TGACGTAGGCCTCGGTGTAGACGG | RT-PCR |
| *hCCL20* | TTTATTGTGGGCTTCACACG<br>GATTTGCGCACACAGACAAC | RT-PCR |
| *hCXCL1* | CAATCCTGCATCCCCCATAGT<br>GGATTTGTCACTGTTCAGCATCTT | RT-PCR |
| *hCXCL2* | CTTGTCTCAACCCCGCATCG<br>CAGTTGGATTTGCCATTTTTCAGC | RT-PCR |
| *hCXCL3* | AGAAAGCTTGTCTCAACCCCG<br>GGTGCTCCCCTTGTTCAGTA | RT-PCR |
| *hCXCL6* | TGTTTACGCGTTACGCTGAG<br>GGCTACCACTTCCACCTTGG | RT-PCR |
| *hCXCL8* | TGGACCCCAAGGAAAACTGG<br>ATGAATTCTCAGCCCTCTTCAA | RT-PCR |
| *hCCL3* | CCAGTTCTCTGCATCACTTGC<br>CACTGGCTGCTCGTCTCAAA | RT-PCR |
| *hCCL11* | ACCCCTTCAGCGACTAGAGA<br>CCACTTCTTCTTGGGGTCGG | RT-PCR |
| *hCCL17* | CTTCAAGGGAGCCATTCCCC<br>CTCTTGTTGTTGGGGTCCGA | RT-PCR |

| Name | Sequence (5′→ 3′) | Purpose |
|---|---|---|
| *hCCL27* | TACAGCAGCATTCCTACTGCC<br>GTCTGAGAGTGGCTTTCGGTA | RT-PCR |
| *hCSF2* | GGGAGCATGTGAATGCCATC<br>CTACTCAGGTTCAGGAGACGC | RT-PCR |
| *hIL36R* | ATGTGGTCCTTGCTGCTC<br>GCATCCATCTGCTGTGAC | RT-PCR |
| *hIL36RN* | ACTCGGCATTGAAGGTGCTT<br>GGGACCACGCTGATCTCTTC | RT-PCR |
| *hIL38* | AGCCAGTACAGCTCACCAAG<br>AAAACTTGGTACGGGCTGAGG | RT-PCR |
| *hFOSL1* | AGCGCAGGCAGCCCAGCAG<br>CCAGCTCTAGGCGCTCCTTC | DNA-PAGE |
| *hPCCA* | TCTGCGGGGACAACAATGGC<br>GCCAACACAGACAGCCTCATC | DNA-PAGE |
| *hIL36G* | AGGAAGGGCCGTCTATCAATC<br>AGCTGCAATGTGGGCTGTTC | DNA-PAGE |
| *hTLE4* | AGGTCAGTCCCATCTTCC<br>ATCCGGGAGTAGAGTTACTGC | DNA-PAGE |
| *hIL17RC* | ACCCACTGCTCTCCGGTGA<br>CCTCATCTTCAGGCTCTTCC | DNA-PAGE |
| *hCD6* | AAGCTTACCTGTGGGACTGC<br>TTGTGCAAACTCCGGGAAGC | DNA-PAGE |
| *hFGFR2* | ACCACATTAGAGCCAGAAGA<br>GGCACAGCATGGAGCCGCT | DNA-PAGE |
| *hIL20RB* | ATTCCAGTGCACCTAGAAAC<br>CTATGAGATCCAGGCCCTGA | DNA-PAGE |
| *hIL17RA* | GCTGCCTCAATGACTGCCTCAG<br>CAGGAACTGGGCGAATTTCAGG | DNA-PAGE |
| *hIL36R-465* | ATTCACCAGGACGAGACTTG<br>AGGGACACTTCCTCCATATC | DNA-PAGE |
| *hIL7R* | GCTGCCTCAATGACTGCCTCAG<br>CAGGAACTGGGCGAATTTCAGG | DNA-PAGE |
| *hIL4R* | ACTTCCCGCTTGGGCGCCCGGACGGCGAAT<br>GCAAGACCTTCATGTTCCCAGAG | DNA-PAGE |
| *hTSLPR* | ATGGGGCGGCTGGTTCTGCTGTG<br>AAGATGGATTTCGGGTCTGGCAC | DNA-PAGE |
| *Ddx5-flox* | TCACATTGCCTTTGCGGCCC<br>GCTAAACCTGTTTCCCCAGT | Genotyping |
| *K14Cre* | ATTTGCCTGCATTACCGGTCG<br>CAGCATTGCTGTCACTTGGTC | Genotyping |
| *sIL36R-P1.* | GGGGCGTGCTGAGCCAGACCTCCAT | Genotyping |
| *sIL36R-P2.* | TCCCGACAAAACCGAAAATCTGTGG | Genotyping |

| Name | Sequence (5′→ 3′) | Purpose |
|---|---|---|
| *sIL36R-P3.* | TGCATCGCATTGTCTGAGTAGG | Genotyping |
| *EIIa Cre* | GCGCTGGAGTTTCAATACCG<br>TTTGACAGGAAACGCAACGG | Genotyping |
| *Il17d-P1* | GCAGCGCATCGCCTTCTATC<br>GTTCACAGTTTAACACCGGAG | Genotyping |
| *Il17d-P2* | CCGAACACTACATCACCATCC<br>GATGTGTGTGATGTGCTTGTATATC | Genotyping |
| *IL36R Exon 2* | GCTAGCAGATGGATGCAAGGACATTTTTATG<br>GGATCCCTTCTCAAGAGTTTTCTATGTTCGTTAAG | mingene |
| *IL36R Exon 3* | GGATCCGTGGATGCTTAGTGCTTTGTTAGAGGCAGAG<br>GATATCCTTTACCAATTCTGCCTTAAAAAATACAAC | mingene |
| *IL36R Exon 4* | GATATCGTCTTCCAGTTAGACTGTAAATTACCTAAATAC<br>CTCGAGTAATGCTCACAGTGATGCCATTTAAAACCTCG | mingene |
| *E4 mutant F1* | GGATCCGTGGATGCTTAGTGCTTTGTTAGAGGCAGAG | ESE deletion minigene |
| *E4 mutant R1* | CTCGAGTAATGCTCACAGTGATGCCATTTAAAACCTCG | ESE deletion minigene |
| *E4 mutant F2* | AATGTCTCGGAACTACGCGT | ESE deletion minigene |
| *E4 mutant R2* | ACGCGTAGTTCCGAGACATT | ESE deletion minigene |
| *SF2* | GCGAATTCATGTCGGGAGGTGGTGTGATTCG<br>GCCTCGAGTTACTTATCGTCGTCATCCTTGTAATCTGTAC<br>GAGAGCGAGATCTGCTATG | Clone |
| *HA-DDX5* | GGCTCGAGATGTCGGGTTATTCGAGTGACCG<br>CCGGATCCTTATTGGGAATATCCTGTTGGC | Clone |
| *4Flag-DDX5* | CCGGATCCATGTCGGGTTATTCGAGTGACCG<br>CCCTCGAGTTATTGGGAATATCCTGTTGGC | Clone |
| *hIL36R* | GGCTCGAGATGTGGTCCTTGCTGCTCTGCGG<br>CCGGATCCTTAGCCAGTCGTGAGAGTACAC | Clone |
| *hsIL36R* | GTGCTCGAGGTATGTGGTCCTTGCTGCTCTG<br>GCGGATCCAAAAGCCTGGTTTCCAAAACAGTG | Clone |
| *mIl36R* | CCGCTCGAGGGGGTTACATCTTTGC<br>CGCGCTAGCTCAGGGAGTTATGAGCCC | Clone |
| *msIl36R* | GCGGATCCATGTGGTCCTTGCTGCTCTGCGGGTTG<br>CCCTCGAGCCAAAAGCCTGGTTTCCAAAACAG | Clone |

## Table 4. *P* values and sample size for RT-PCR, ELISA and FACS

| | | | |
|---|---|---|---|
| **Fig. 1a** | | | |
| Normal vs AD | | $p=0.0021$ | Normal $n=38$, AD $n=27$ |
| Normal vs PSO | | $p<0.0001$ | Normal $n=38$, Psoriasis $n=28$ |
| **Fig. 1g** | | | |
| Keratinocytes | Healthy vs AD lesion | $p<0.0001$ | Healthy $n=51938$, AD lesion $n=11255$ |
| | Healthy vs AD no lesion | $p<0.0001$ | Healthy $n=51938$, AD no lesion $n=20238$ |
| | Healthy vs Pso lesion | $p<0.0001$ | Healthy $n=51938$, Pso lesion $n=12978$ |
| | Healthy vs Pso no lesion | $p<0.0001$ | Healthy $n=51938$, Pso no lesion $n=21064$ |
| Fibroblasts | Healthy vs AD lesion | $p<0.0001$ | Healthy $n=17312$, AD lesion $n=10028$ |
| | Healthy vs AD no lesion | $p<0.0001$ | Healthy $n=17312$, AD no lesion $n=7337$ |
| | Healthy vs Pso lesion | $p<0.0001$ | Healthy $n=17312$, Pso lesion $n=12029$ |
| | Healthy vs Pso no lesion | $p<0.0001$ | Healthy $n=17312$, Pso no lesion $n=14206$ |
| Lymphocytes | Healthy vs AD lesion | $p<0.0001$ | Healthy $n=41756$, AD lesion $n=18649$ |
| | Healthy vs AD no lesion | $p<0.0001$ | Healthy $n=41756$, AD no lesion $n=10732$ |
| | Healthy vs Pso lesion | $p<0.0001$ | Healthy $n=41756$, Pso lesion $n=17592$ |
| | Healthy vs Pso no lesion | $p= 0.0186$ | Healthy $n=41756$, Pso no lesion $n=5188$ |
| **Fig. 1l** | | | |
| *Il4* | | $p=0.0125$ | $Ddx5^{fl/fl}$ $n=5$, $Ddx5^{\Delta/kc}$ $n=5$ |
| *Il13* | | $p=0.0111$ | $Ddx5^{fl/fl}$ $n=5$, $Ddx5^{\Delta/kc}$ $n=5$ |
| *Tslp* | | $p=0.0605$ | $Ddx5^{fl/fl}$ $n=5$, $Ddx5^{\Delta/kc}$ $n=5$ |
| *Ccl11* | | $p=0.0012$ | $Ddx5^{fl/fl}$ $n=5$, $Ddx5^{\Delta/kc}$ $n=5$ |
| *Ccl17* | | $p=0.0073$ | $Ddx5^{fl/fl}$ $n=5$, $Ddx5^{\Delta/kc}$ $n=5$ |
| *Ccl22* | | $p=0.0019$ | $Ddx5^{fl/fl}$ $n=5$, $Ddx5^{\Delta/kc}$ $n=5$ |
| **Fig. 1m** | | | |
| $CD45^+$ cells in ear (%) | | $p=0.0002$ | $Ddx5^{fl/fl}$ $n=6$, $Ddx5^{\Delta/kc}$ $n=6$ |
| $CD45^+$ cells/5x$10^4$ cells (x$10^3$) | | $p=0.0003$ | $Ddx5^{fl/fl}$ $n=6$, $Ddx5^{\Delta/kc}$ $n=6$ |
| Eosinophils in ear (%) | | $p=0.0227$ | $Ddx5^{fl/fl}$ $n=6$, $Ddx5^{\Delta/kc}$ $n=6$ |
| Eosinophils/5x$10^4$ cells (x$10^3$) | | $p=0.0004$ | $Ddx5^{fl/fl}$ $n=6$, $Ddx5^{\Delta/kc}$ $n=6$ |

| | | |
|---|---|---|
| Basophils in ear (%) | $p$=0.0364 | $Ddx5^{fl/fl}$ $n$=6, $Ddx5^{\Delta/kc}$ $n$=6 |
| Basophils/5x$10^4$ cells (x$10^2$) | $p$=0.0003 | $Ddx5^{fl/fl}$ $n$=6, $Ddx5^{\Delta/kc}$ $n$=6 |
| CD4$^+$ T cells in ear (%) | $p$=0.0014 | $Ddx5^{fl/fl}$ $n$=6, $Ddx5^{\Delta/kc}$ $n$=6 |
| CD4$^+$T cells/5x$10^4$ cells (x$10^2$) | $p$=0.0005 | $Ddx5^{fl/fl}$ $n$=6, $Ddx5^{\Delta/kc}$ $n$=6 |
| **Fig. 2a** | | |
| *DDX5*-8h vs 0h | $p$=0.1599 | $n$=3 |
| *DDX5*-16h vs 0h | $p$=0.0074 | $n$=3 |
| *DDX5*-24h vs 0h | $p$=0.0035 | $n$=3 |
| *DDX5*-32h vs 0h | $p$=0.0321 | $n$=3 |
| *DDX5*-40h vs 0h | $p$=0.0280 | $n$=3 |
| **Fig. 2g** | | |
| *Il4* | $p$=0.0418 | $Ddx5^{fl/fl}$ $n$=5, $Ddx5^{\Delta/kc}$ $n$=5 |
| *Il13* | $p$=0.0220 | $Ddx5^{fl/fl}$ $n$=5, $Ddx5^{\Delta/kc}$ $n$=5 |
| *Tslp* | $p$=0.1448 | $Ddx5^{fl/fl}$ $n$=5, $Ddx5^{\Delta/kc}$ $n$=5 |
| *Ccl11* | $p$=0.0156 | $Ddx5^{fl/fl}$ $n$=5, $Ddx5^{\Delta/kc}$ $n$=5 |
| *Ccl17* | $p$=0.0435 | $Ddx5^{fl/fl}$ $n$=5, $Ddx5^{\Delta/kc}$ $n$=5 |
| *Ccl22* | $p$=0.0946 | $Ddx5^{fl/fl}$ $n$=5, $Ddx5^{\Delta/kc}$ $n$=5 |
| **Fig. 2h** | | |
| CD45$^+$ cells in ear (%) | $p$=0.0228 | WT $n$=5, $Il17d^{-/-}$ $n$=5 |
| CD45$^+$ cells/$10^5$ cells (x$10^4$) | $p$=0.0238 | WT $n$=5, $Il17d^{-/-}$ $n$=5 |
| Eosinophils in ear (%) | $p$=0.0267 | WT $n$=5, $Il17d^{-/-}$ $n$=5 |
| Eosinophils/$10^5$ cells (x$10^2$) | $p$=0.0187 | WT $n$=5, $Il17d^{-/-}$ $n$=5 |
| Basophils in ear (%) | $p$<0.0001 | WT $n$=5, $Il17d^{-/-}$ $n$=5 |
| Basophils/$10^5$ cells (x$10^2$) | $p$<0.0001 | WT $n$=5, $Il17d^{-/-}$ $n$=5 |
| CD4$^+$ T cells in ear (%) | $p$=0.0001 | WT $n$=5, $Il17d^{-/-}$ $n$=5 |
| CD4$^+$T cells/$10^5$ cells (x$10^2$) | $p$=0.0050 | WT $n$=5, $Il17d^{-/-}$ $n$=5 |
| **Fig. 3b** | | |
| *Il4* | $p$=0.044 | WT $n$=7, $Cd93^{-/-}$ $n$=7 |
| *Il13* | $p$=0.0086 | WT $n$=7, $Cd93^{-/-}$ $n$=7 |
| *Tslp* | $p$=0.3549 | WT $n$=7, $Cd93^{-/-}$ $n$=7 |
| *Ccl11* | $p$=0.0141 | WT $n$=7, $Cd93^{-/-}$ $n$=7 |
| *Ccl17* | $p$=0.0041 | WT $n$=7, $Cd93^{-/-}$ $n$=7 |
| *Ccl22* | $p$=0.0080 | WT $n$=7, $Cd93^{-/-}$ $n$=7 |
| **Fig. 3e** | | |
| *DDX5* | | |
| DMSO vs iSTAT3 | $p$=0.9854 | $n$=3 |
| DMSO vs iP38 | $p$=0.5576 | $n$=3 |
| DMSO vs iPI3K | $p$<0.0001 | $n$=3 |
| DMSO vs iMEK | $p$=0.0019 | $n$=3 |
| DMSO vs iJNK | $p$=0.0002 | $n$=3 |
| DMSO vs iPPARγ | $p$<0.0001 | $n$=3 |
| DMSO vs iNFκB | $p$=0.1665 | $n$=3 |
| DMSO vs iSMAD2/3 | $p$=0.1370 | $n$=3 |

| | | |
|---|---|---|
| DMSO vs iAKT1/2 | *p*<0.0001 | *n*=3 |
| DMSO vs IL-17D | *p*<0.0001 | *n*=3 |
| IL-17D vs IL-17D+iSTAT3 | *p*>0.9999 | *n*=3 |
| IL-17D vs IL-17D+iP38 | *p*<0.0001 | *n*=3 |
| IL-17D vs IL-17D+iPI3K | *p*>0.9999 | *n*=3 |
| IL-17D vs IL-17D+iMEK | *p*=0.4616 | *n*=3 |
| IL-17D vs IL-17D+iJNK | *p*<0.0001 | *n*=3 |
| IL-17D vs IL-17D+iPPARγ | *p*=0.4568 | *n*=3 |
| IL-17D vs IL-17D+iNFκB | *p*=0.3549 | *n*=3 |
| IL-17D vs IL-17D+iSMAD2/3 | *p*=0.0292 | *n*=3 |
| IL-17D vs IL-17D+iAKT1/2 | *p*=0.0003 | n=3 |

**Fig. 4c**

| | | | |
|---|---|---|---|
| *CCL11* | 0h:WT vs 0h:*DDX5*$^{-/-}$ | *p*=0.0435 | *n*=3 |
| | 0h:*DDX5*$^{-/-}$ vs 6h:*DDX5*$^{-/-}$ | *p*=0.0197 | *n*=3 |
| | 6h:WT vs 6h:*DDX5*$^{-/-}$ | *p*=0.0002 | *n*=3 |
| | 6h:*DDX5*$^{-/-}$vs 6h:*DDX5*$^{-/-}$siIL-36R | *p*=0.0003 | *n*=3 |
| *CCL17* | 0h:WT vs 0h:*DDX5*$^{-/-}$ | *p*=0.8089 | *n*=3 |
| | 0h:*DDX5*$^{-/-}$ vs 6h:*DDX5*$^{-/-}$ | *p*=0.0004 | *n*=3 |
| | 6h:WT vs 6h:*DDX5*$^{-/-}$ | *p*=0.0023 | *n*=3 |
| | 6h:*DDX5*$^{-/-}$vs 6h:*DDX5*$^{-/-}$+siIL-36R | *p*=0.0156 | *n*=3 |
| *CCL22* | 0h:WT vs 0h:*DDX5*$^{-/-}$ | *p*=0.9492 | *n*=3 |
| | 0h:*DDX5*$^{-/-}$ vs 6h:*DDX5*$^{-/-}$ | *p*=0.0013 | *n*=3 |
| | 6h:WT vs 6h:*DDX5*$^{-/-}$ | *p*=0.0159 | *n*=3 |
| | 6h:*DDX5*$^{-/-}$vs 6h:*DDX5*$^{-/-}$+siIL-36R | *p*=0.0072 | *n*=3 |

**Fig. 4d**

| | | | |
|---|---|---|---|
| *CCL20* | 0h:WT vs 2h:WT | *p*< 0.0001 | *n*=3 |
| | 0h:*DDX5*$^{-/-}$vs 2h:*DDX5*$^{-/-}$ | *p*< 0.0001 | *n*=3 |
| | 6h:WT vs 2h:*DDX5*$^{-/-}$ | *p*=0.0001 | *n*=3 |
| | 6h:*DDX5*$^{-/-}$vs 2h:*DDX5*$^{-/-}$siIL-36R | *p*< 0.0001 | *n*=3 |
| *CXCL1* | 0h:WT vs 2h:WT | *p*< 0.0001 | *n*=3 |
| | 0h:*DDX5*$^{-/-}$vs 2h:*DDX5*$^{-/-}$ | *p*< 0.0001 | *n*=3 |
| | 2h:WT vs 2h:*DDX5*$^{-/-}$ | *p*< 0.0001 | *n*=3 |
| | 2h:*DDX5*$^{-/-}$vs 2h:*DDX5*$^{-/-}$siIL-36R | *p*< 0.0001 | *n*=3 |
| *CXCL2* | 0h:WT vs 2h:WT | *p*< 0.0001 | *n*=3 |
| | 0h:*DDX5*$^{-/-}$ vs 2h:*DDX5*$^{-/-}$ | *p*< 0.0001 | *n*=3 |
| | 2h:WT vs 2h:*DDX5*$^{-/-}$ | *p*< 0.0001 | *n*=3 |
| | 2h:*DDX5*$^{-/-}$vs 2h:*DDX5*$^{-/-}$siIL-36R | *p*< 0.0001 | *n*=3 |

**Fig. 4e**

| | | | |
|---|---|---|---|
| *hIL36R* | WT vs *DDX5*$^{-/-}$ | *p*< 0.0001 | *n*=9 |

**Fig. 4f**

| | | | |
|---|---|---|---|
| *mIl36R* | WT vs *Ddx5*$^{-/-}$ | *p*=0.0012 | *n*=3 |

**Fig. 7c**

| | | | |
|---|---|---|---|
| *CCL20* | Blank:EV vs. IL-36γ:EV | *p*<0.0001 | *n*=3 |

| | | | |
|---|---|---|---|
| | Blank:sIL-36R vs. IL-36γ:sIL-36R | $p=0.0001$ | $n=3$ |
| | IL-36γ:EV vs. IL-36γ:sIL-36R | $p<0.0001$ | $n=3$ |
| *CXCL1* | Blank:EV vs. IL-36γ:EV | $p<0.0001$ | $n=3$ |
| | Blank:sIL-36R vs. IL-36γ:sIL-36R | $p<0.0001$ | $n=3$ |
| | IL-36γ:EV vs. IL-36γ:sIL-36R | $p<0.0001$ | $n=3$ |
| *CXCL2* | Blank:EV vs. IL-36γ:EV | $p<0.0001$ | $n=3$ |
| | Blank:sIL-36R vs. IL-36γ:sIL-36R | $p=0.0010$ | $n=3$ |
| | IL-36γ:EV vs. IL-36γ:sIL-36R | $p<0.0001$ | $n=3$ |
| *CCL3* | Blank:EV vs. IL-36γ:EV | $p<0.0001$ | $n=3$ |
| | Blank:sIL-36R vs. IL-36γ:sIL-36R | $p=0.0011$ | $n=3$ |
| | IL-36γ:EV vs. IL-36γ:sIL-36R | $p<0.0001$ | $n=3$ |
| *CCL17* | Blank:EV vs. IL-36γ:EV | $p<0.0001$ | $n=3$ |
| | Blank:sIL-36R vs. IL-36γ:sIL-36R | $p<0.0001$ | $n=3$ |
| | IL-36γ:EV vs. IL-36γ:sIL-36R | $p<0.0001$ | $n=3$ |
| *CCL27* | Blank:EV vs. IL-36γ:EV | $p=0.0007$ | $n=3$ |
| | Blank:sIL-36R vs. IL-36γ:sIL-36R | $p=0.0607$ | $n=3$ |
| | IL-36γ:EV vs. IL-36γ:sIL-36R | $p=0.0071$ | $n=3$ |

| | | |
|---|---|---|
| **Fig. 7d** | | |
| PBS *vs* sIL-36R | $p<0.0001$ | PBS $n=5$, sIL-36R $n=5$ |
| **Fig. 7e** | | |
| *Il4* | $p=0.0189$ | PBS $n=5$, sIL-36R $n=5$ |
| *Il13* | $p=0.0010$ | PBS $n=5$, sIL-36R $n=5$ |
| *Tslp* | $p=0.0022$ | PBS $n=5$, sIL-36R $n=5$ |
| *Ccl11* | $p=0.0114$ | PBS $n=5$, sIL-36R $n=5$ |
| *Ccl17* | $p=0.0110$ | PBS $n=5$, sIL-36R $n=5$ |
| **Fig. 7f** | | |
| CD45$^+$ cells in ear (%) | $p=0.0376$ | PBS $n=5$, sIL-36R $n=5$ |
| CD45$^+$ cells/$10^5$ cells (x$10^3$) | $p=0.0010$ | PBS $n=5$, sIL-36R $n=5$ |
| Eosinophils in ear (%) | $p<0.0001$ | PBS $n=5$, sIL-36R $n=5$ |
| Eosinophils/$10^5$ cells (x$10^2$) | $p=0.0001$ | PBS $n=5$, sIL-36R $n=5$ |
| Basophils in ear (%) | $p=0.5654$ | PBS $n=5$, sIL-36R $n=5$ |
| Basophils/$10^5$ cells (x$10^2$) | $p<0.0001$ | PBS $n=5$, sIL-36R $n=5$ |
| CD4$^+$ T cells in ear (%) | $p=0.0147$ | PBS $n=5$, sIL-36R $n=5$ |
| CD4$^+$T cells/$10^5$ cells (x$10^3$) | $p=0.0088$ | PBS $n=5$, sIL-36R $n=5$ |
| **Fig. 8a** | | |
| PBS *vs* sIL-36R | $p<0.0001$ | PBS $n=4$, sIL-36R $n=4$ |
| **Fig. 8b** | | |
| PBS *vs* sIL-36R | $p=0.0005$ | PBS $n=10$, sIL-36R $n=10$ |
| **Fig. 8c** | | |
| IL-4 | $p=0.0029$ | PBS $n=4$, sIL-36R $n=4$ |
| IL-13 | $p=0.0005$ | PBS $n=4$, sIL-36R $n=4$ |
| TSLP | $p=0.0014$ | PBS $n=4$, sIL-36R $n=4$ |
| **Fig. 8d** | | |

| | | | |
|---|---|---|---|
| *Ccl11* | | $p=0.0255$ | PBS $n=4$, sIL-36R $n=4$ |
| *Ccl17* | | $p=0.0243$ | PBS $n=4$, sIL-36R $n=4$ |
| *Ccl17* | | $p=0.0215$ | PBS $n=4$, sIL-36R $n=4$ |
| **Fig. 8e** | | | |
| CCL20 | | $p<0.0001$ | PBS $n=7$, sIL-36R $n=7$ |
| CXCL1 | | $p<0.0001$ | PBS $n=7$, sIL-36R $n=7$ |
| IL-23 | | $p<0.0001$ | PBS $n=7$, sIL-36R $n=7$ |
| IL-17A | | $p<0.0001$ | PBS $n=7$, sIL-36R $n=7$ |
| IL-17F | | $p=0.0004$ | PBS $n=7$, sIL-36R $n=7$ |
| TNF | | $p=0.0005$ | PBS $n=7$, sIL-36R $n=7$ |
| **Fig. 8f** | | | |
| *Ddx5*$^{fl/fl}$ vs *Ddx5*$^{\Delta/kc}$ | | $p<0.0001$ | *Ddx5*$^{fl/fl}$ $n=8$, *Ddx5*$^{\Delta/kc}$ $n=8$ |
| *Ddx5*$^{\Delta/kc}$ vs *Ddx5*$^{\Delta/kc}$*sIL36R*$^{Tg/kc}$ | | $p<0.0001$ | *Ddx5*$^{\Delta/kc}$ $n=8$, *Ddx5*$^{\Delta/kc}$*sIL36R*$^{Tg/kc}$ $n=8$ |
| *Ddx5*$^{fl/fl}$ vs *Ddx5*$^{\Delta/kc}$*sIL36R*$^{Tg/kc}$ | | $p=0.9444$ | *Ddx5*$^{fl/fl}$ $n=8$, *Ddx5*$^{\Delta/kc}$*sIL36R*$^{Tg/kc}$ $n=8$ |
| **Fig. 8g** | | | |
| IL-4 | *Ddx5*$^{fl/fl}$ vs *Ddx5*$^{\Delta/kc}$ | $p=0.0005$ | *Ddx5*$^{fl/fl}$ $n=4$, *Ddx5*$^{\Delta/kc}$ $n=4$ |
| | *Ddx5*$^{\Delta/kc}$ vs *Ddx5*$^{\Delta/kc}$*sIL36R*$^{Tg/kc}$ | $p=0.0004$ | *Ddx5*$^{\Delta/kc}$ $n=4$, *Ddx5*$^{\Delta/kc}$*sIL36R*$^{Tg/kc}$ $n=4$ |
| IL-13 | *Ddx5*$^{fl/fl}$ vs *Ddx5*$^{\Delta/kc}$ | $p=0.0071$ | *Ddx5*$^{fl/fl}$ $n=4$, *Ddx5*$^{\Delta/kc}$ $n=4$ |
| | *Ddx5*$^{\Delta/kc}$ vs *Ddx5*$^{\Delta/kc}$*sIL36R*$^{Tg/kc}$ | $p=0.0002$ | *Ddx5*$^{\Delta/kc}$,$n=4$, *Ddx5*$^{\Delta/kc}$*sIL36R*$^{Tg/kc}$ $n=4$ |
| TSLP | *Ddx5*$^{fl/fl}$ vs *Ddx5*$^{\Delta/kc}$ | $p<0.0001$ | *Ddx5*$^{fl/fl}$ $n=4$, *Ddx5*$^{\Delta/kc}$ $n=4$ |
| | *Ddx5*$^{\Delta/kc}$ vs *Ddx5*$^{\Delta/kc}$*sIL36R*$^{Tg/kc}$ | $p<0.0001$ | *Ddx5*$^{\Delta/kc}$ $n=4$, *Ddx5*$^{\Delta/kc}$*sIL36R*$^{Tg/kc}$ $n=4$ |
| **Fig. 8h** | | | |
| *Ccl11* | *Ddx5*$^{fl/fl}$ vs *Ddx5*$^{\Delta/kc}$ | $p=0.0079$ | *Ddx5*$^{fl/fl}$ $n=4$, *Ddx5*$^{\Delta/kc}$ $n=4$ |
| | *Ddx5*$^{\Delta/kc}$ vs *Ddx5*$^{\Delta/kc}$*sIL36R*$^{Tg/kc}$ | $p=0.0076$ | *Ddx5*$^{\Delta/kc}$ $n=4$, *Ddx5*$^{\Delta/kc}$*sIL36R*$^{Tg/kc}$ $n=4$ |
| *Ccl17* | *Ddx5*$^{fl/fl}$ vs *Ddx5*$^{\Delta/kc}$ | $p=0.0002$ | *Ddx5*$^{fl/fl}$ $n=4$, *Ddx5*$^{\Delta/kc}$ $n=4$ |
| | *Ddx5*$^{\Delta/kc}$ vs *Ddx5*$^{\Delta/kc}$*sIL36R*$^{Tg/kc}$ | $p=0.0001$ | *Ddx5*$^{\Delta/kc}$ $n=4$, *Ddx5*$^{\Delta/kc}$*sIL36R*$^{Tg/kc}$ $n=4$ |
| *Ccl22* | *Ddx5*$^{fl/fl}$ vs *Ddx5*$^{\Delta/kc}$ | $p=0.0006$ | *Ddx5*$^{fl/fl}$ $n=4$, *Ddx5*$^{\Delta/kc}$ $n=4$ |
| | *Ddx5*$^{\Delta/kc}$ vs *Ddx5*$^{\Delta/kc}$*sIL36R*$^{Tg/kc}$ | $p=0.0340$ | *Ddx5*$^{\Delta/kc}$,$n=4$, *Ddx5*$^{\Delta/kc}$*sIL36R*$^{Tg/kc}$ $n=4$ |
| **Fig. 8i** | | | |
| *Ddx5*$^{fl/fl}$ vs *Ddx5*$^{\Delta/kc}$ | | $p<0.0001$ | *Ddx5*$^{fl/fl}$ $n=8$, *Ddx5*$^{\Delta/kc}$ $n=8$ |
| *Ddx5*$^{\Delta/kc}$ vs *Ddx5*$^{\Delta/kc}$*sIL36R*$^{Tg/kc}$ | | $p<0.0001$ | *Ddx5*$^{fl/fl}$ $n=8$, *Ddx5*$^{\Delta/kc}$ $n=8$ |
| *Ddx5*$^{fl/fl}$ vs *Ddx5*$^{\Delta/kc}$*sIL36R*$^{Tg/kc}$ | | $p<0.0001$ | *Ddx5*$^{fl/fl}$ $n=8$, *Ddx5*$^{\Delta/kc}$ $n=8$ |
| **Fig. 8j** | | | |

| | | | |
|---|---|---|---|
| CCL20 | $Ddx5^{fl/fl}$ vs $Ddx5^{\Delta/kc}$ | $p$<0.0001 | $Ddx5^{fl/fl}$ $n$=4, $Ddx5^{\Delta/kc}$ $n$=4 |
| | $Ddx5^{\Delta/kc}$ vs $Ddx5^{\Delta/kc}sIL36R^{Tg/kc}$ | $p$=0.0004 | $Ddx5^{\Delta/kc}$ $n$=4, $Ddx5^{\Delta/kc}sIL36R^{Tg/kc}$ $n$=4 |
| CXCL1 | $Ddx5^{fl/fl}$ vs $Ddx5^{\Delta/kc}$ | $p$<0.0001 | $Ddx5^{fl/fl}$, $n$=4, $Ddx5^{\Delta/kc}$, $n$=4 |
| | $Ddx5^{\Delta/kc}$ vs $Ddx5^{\Delta/kc}sIL36R^{Tg/kc}$ | $p$=0.0006 | $Ddx5^{\Delta/kc}$ $n$=4, $Ddx5^{\Delta/kc}sIL36R^{Tg/kc}$ $n$=4 |
| IL-23 | $Ddx5^{fl/fl}$ vs $Ddx5^{\Delta/kc}$ | $p$=0.0002 | $Ddx5^{fl/fl}$ $n$=4, $Ddx5^{\Delta/kc}$ $n$=4 |
| | $Ddx5^{\Delta/kc}$ vs $Ddx5^{\Delta/kc}sIL36R^{Tg/kc}$ | $p$=0.0002 | $Ddx5^{\Delta/kc}$ $n$=4, $Ddx5^{\Delta/kc}sIL36R^{Tg/kc}$ $n$=4 |
| IL-17A | $Ddx5^{fl/fl}$ vs $Ddx5^{\Delta/kc}$ | $p$<0.0001 | $Ddx5^{fl/fl}$ $n$=4, $Ddx5^{\Delta/kc}$ $n$=4 |
| | $Ddx5^{\Delta/kc}$ vs $Ddx5^{\Delta/kc}sIL36R^{Tg/kc}$ | $p$<0.0001 | $Ddx5^{\Delta/kc}$ $n$=4, $Ddx5^{\Delta/kc}sIL36R^{Tg/kc}$ $n$=4 |
| **Extended Data Fig.1b** | | | |
| *CDC5L* | PSO Ctrl vs PSO Lesion | $p$=0.2822 | PSO Ctrl $n$=4, PSO Lesion $n$=4 |
| | AD Ctrl vs AD Lesion | $p$=0.5343 | AD Ctrl $n$=4, AD Lesion $n$=4 |
| *PRPF18* | PSO Ctrl vs PSO Lesion | $p$=0.3837 | PSO Ctrl $n$=4, PSO Lesion $n$=4 |
| | AD Ctrl vs AD Lesion | $p$=0.9103 | AD Ctrl $n$=4, AD Lesion $n$=4 |
| *DHX8* | PSO Ctrl vs PSO Lesion | $p$=0.0620 | PSO Ctrl $n$=4, PSO Lesion $n$=4 |
| | AD Ctrl vs AD Lesion | $p$=0.9387 | AD Ctrl $n$=4, AD Lesion $n$=4 |
| *DHX15* | PSO Ctrl vs PSO Lesion | $p$=0.5744 | PSO Ctrl $n$=4, PSO Lesion $n$=4 |
| | AD Ctrl vs AD Lesion | $p$=0.0391 | AD Ctrl $n$=4, AD Lesion $n$=4 |
| *HNRNPa1* | PSO Ctrl vs PSO Lesion | $p$=0.9292 | PSO Ctrl $n$=4, PSO Lesion $n$=4 |
| | AD Ctrl vs AD Lesion | $p$=0.8885 | AD Ctrl $n$=4, AD Lesion $n$=4 |
| *SRSF1* | PSO Ctrl vs PSO Lesion | $p$> 0.9999 | PSO Ctrl $n$=4, PSO Lesion $n$=4 |
| | AD Ctrl vs AD Lesion | $p$=0.0001 | AD Ctrl $n$=4, AD Lesion $n$=4 |
| **Extended Data Fig.1d** | | | |
| *Ddx5* | Day0 vs Day4 | $p$=0.0012 | Day0 $n$=4, Day4 $n$=3 |
| | Day0 vs Day8 | $p$=0.0169 | Day0 $n$=4, Day8 $n$=3 |
| | Day0 vs Day12 | $p$=0.0023 | Day0 $n$=4, Day12 $n$=3 |
| | Day0 vs Day16 | $p$=0.0214 | Day0 $n$=4, Day16 $n$=3 |
| **Extended Data Fig.1e** | | | |
| *Ddx5* | Day0 vs Day1 | $p$=0.0272 | Day0 $n$=3, Day1 $n$=3 |
| | Day0 vs Day2 | $p$=0.0277 | Day0 $n$=3, Day2 $n$=3 |
| | Day0 vs Day3 | $p$=0.0084 | Day0 $n$=3, Day3 $n$=3 |
| | Day0 vs Day4 | $p$=0.0177 | Day0 $n$=3, Day4 $n$=3 |
| | Day0 vs Day5 | $p$=0.0034 | Day0 $n$=3, Day5 $n$=3 |

| | | |
|---|---|---|
| **Extended Data Fig. 2b** | | |
| $Ddx5^{fl/fl}$ vs $Ddx5^{\Delta/kc}$ | $p$=0.0019 | $Ddx5^{fl/fl}$ $n$=5, $Ddx5^{\Delta/kc}$ $n$=5 |
| **Extended Data Fig. 2d** | | |
| $Ddx5^{fl/fl}$ vs $Ddx5^{\Delta/kc}$ | $p$=0.0004 | $Ddx5^{fl/fl}$ $n$=5, $Ddx5^{\Delta/kc}$ $n$=5 |
| **Extended Data Fig. 2e** | | |
| *Il23* | $p$=0.0117 | $Ddx5^{fl/fl}$ $n$=5, $Ddx5^{\Delta/kc}$ $n$=5 |
| *Il17a* | $p$=0.0020 | $Ddx5^{fl/fl}$ $n$=5, $Ddx5^{\Delta/kc}$ $n$=5 |
| *Ccl20* | $p$=0.0425 | $Ddx5^{fl/f}$, $n$=5, $Ddx5^{\Delta/kc}$ $n$=5 |
| *Cxcl1* | $p$=0.0378 | $Ddx5^{fl/fl}$ $n$=5, $Ddx5^{\Delta/kc}$ $n$=5 |
| *Cxcl2* | $p$=0.0246 | $Ddx5^{fl/fl}$ $n$=5, $Ddx5^{\Delta/kc}$ $n$=5 |
| *S100a7* | $p$=0.0027 | $Ddx5^{fl/fl}$ $n$=5, $Ddx5^{\Delta/kc}$ $n$=5 |
| **Extended Data Fig. 2f** | | |
| $CD45^+$ cells in skin (%) | $p$<0.0001 | $Ddx5^{fl/fl}$ $n$=5, $Ddx5^{\Delta/kc}$ $n$=5 |
| $CD45^+$ cells/$10^5$ cells (x$10^4$) | $p$=0.0003 | $Ddx5^{fl/fl}$ $n$=5, $Ddx5^{\Delta/kc}$ $n$=5 |
| Neutrophils in skin (%) | $p$=0.0485 | $Ddx5^{fl/fl}$ $n$=5, $Ddx5^{\Delta/kc}$ $n$=5 |
| Neutrophils/$10^5$ cells (x$10^2$) | $p$=0.0024 | $Ddx5^{fl/fl}$ $n$=5, $Ddx5^{\Delta/kc}$ $n$=5 |
| $MHCII^+$ antigen-presenting cell in skin (%) | $p$=0.0006 | $Ddx5^{fl/fl}$ $n$=5, $Ddx5^{\Delta/kc}$ $n$=5 |
| $MHCII^+$ antigen-presenting cell/$10^5$ cells (x$10^2$) | $p$=0.0003 | $Ddx5^{fl/fl}$ $n$=5, $Ddx5^{\Delta/kc}$ $n$=5 |
| $CD3^+\gamma\delta T^+$ cells in skin (%) | $p$=0.0130 | $Ddx5^{fl/fl}$ $n$=5, $Ddx5^{\Delta/kc}$ $n$=5 |
| $CD3^+\gamma\delta T^+$ cells/$10^5$ cells (x$10^2$) | $p$=0.0016 | $Ddx5^{fl/fl}$ $n$=5, $Ddx5^{\Delta/kc}$ $n$=5 |
| **Extended Data Fig. 3a** | | |
| TNF | | |
| 0ng/mL vs 20ng/mL | $p$=0.0509 | $n$=2 |
| 0ng/mL vs 40ng/mL | $p$=0.0779 | $n$=2 |
| 0ng/mL vs 60ng/mL | $p$=0.4187 | $n$=2 |
| 0ng/mL vs 80ng/mL | $p$=0.4027 | $n$=2 |
| 0ng/mL vs 100ng/mL | $p$=0.1596 | $n$=2 |
| IL-1β | | |
| 0ng/mL vs 20ng/mL | $p$>0.9999 | $n$=3 |
| 0ng/mL vs 40ng/mL | $p$=0.3841 | $n$=3 |
| 0ng/mL vs 60ng/mL | $p$=0.7999 | $n$=3 |
| 0ng/mL vs 80ng/mL | $p$=0.3296 | $n$=3 |
| 0ng/mL vs 100ng/mL | $p$=0.6662 | $n$=3 |
| IL-36γ | | |
| 0ng/mL vs 20ng/mL | $p$=0.8200 | $n$=3 |
| 0ng/mL vs 40ng/mL | $p$=0.5913 | $n$=3 |
| 0ng/mL vs 60ng/mL | $p$=0.9722 | $n$=3 |
| 0ng/mL vs 80ng/mL | $p$=0.9402 | $n$=3 |
| 0ng/mL vs 100ng/mL | $p$=0.8876 | $n$=3 |
| IL-4 | | |
| 0ng/mL vs 20ng/mL | $p$=0.9955 | $n$=3 |

| | | |
|---|---|---|
| 0ng/mL vs 40ng/mL | *p*=0.6971 | *n*=3 |
| 0ng/mL vs 60ng/mL | *p*=0.3423 | *n*=3 |
| 0ng/mL vs 80ng/mL | *p*=0.6748 | *n*=3 |
| 0ng/mL vs 100ng/mL | *p*=0.9802 | *n*=3 |
| IL-13 | | |
| 0ng/mL vs 20ng/mL | *p*=0.2427 | *n*=3 |
| 0ng/mL vs 40ng/mL | *p*=0.2895 | *n*=3 |
| 0ng/mL vs 60ng/mL | *p*=0.0489 | *n*=3 |
| 0ng/mL vs 80ng/mL | *p*=0.7662 | *n*=3 |
| 0ng/mL vs 100ng/mL | *p*=0.9821 | *n*=3 |
| IL-17A | | |
| 0nM vs 0.46nM | *p*=0.9998 | *n*=3 |
| 0nM vs 0.91nM | *p*=0.8805 | *n*=3 |
| 0nM vs 1.38nM | *p*=0.4236 | *n*=3 |
| 0nM vs 1.84nM | *p*=0.9536 | *n*=3 |
| 0nM vs 2.30nM | *p*=0.4558 | *n*=3 |
| IL-17B | | |
| 0nM vs 0.46nM | *p*=0.0037 | *n*=3 |
| 0nM vs 0.91nM | *p*=0.0385 | *n*=3 |
| 0nM vs 1.38nM | *p*=0.0811 | *n*=3 |
| 0nM vs 1.84nM | *p*=0.3786 | *n*=3 |
| 0nM vs 2.30nM | *p*=0.0095 | *n*=3 |
| IL-17C | | |
| 0nM vs 0.46nM | *p*=0.9961 | *n*=3 |
| 0nM vs 0.91nM | *p*=0.8707 | *n*=3 |
| 0nM vs 1.38nM | *p*=0.9623 | *n*=3 |
| 0nM vs 1.84nM | *p*=0.4608 | *n*=3 |
| 0nM vs 2.30nM | *p*=0.9980 | *n*=3 |
| IL-17E | | |
| 0nM vs 0.46nM | *p*=0.1900 | *n*=3 |
| 0nM vs 0.91nM | *p*=0.0014 | *n*=3 |
| 0nM vs 1.38nM | *p*=0.1833 | *n*=3 |
| 0nM vs 1.84nM | *p*=0.0207 | *n*=3 |
| 0nM vs 2.30nM | *p*=0.0021 | *n*=3 |
| IL-17F | | |
| 0nM vs 0.46nM | *p*=0.9432 | *n*=3 |
| 0nM vs 0.91nM | *p*=0.9999 | *n*=3 |
| 0nM vs 1.38nM | *p*=0.8687 | *n*=3 |
| 0nM vs 1.84nM | *p*=0.6466 | *n*=3 |
| 0nM vs 2.30nM | *p*=0.6246 | *n*=3 |
| **Extended Data Fig.4e** | | |
| $CD45^+$ cells in ear (%) | *p*=0.0022 | WT *n*=5, *Il17d*$^{-/-}$ *n*=5 |
| $CD45^+$ cells/$10^5$ cells (x$10^3$) | *p*=0.0006 | WT *n*=5, *Il17d*$^{-/-}$ *n*=5 |

| | | |
|---|---|---|
| **Extended Data Fig.5c** | | |
| *Cxcl1* | *p*=0.0613 | WT *n*=5, *Cd93*$^{-/-}$ *n*=4 |
| *Cxcl2* | *p*=0.0003 | WT *n*=5, *Cd93*$^{-/-}$ *n*=4 |
| *Ccl20* | *p*=0.0019 | WT *n*=5, *Cd93*$^{-/-}$ *n*=4 |
| *Il17a* | *p*=0.0978 | WT *n*=5, *Cd93*$^{-/-}$ *n*=4 |
| *Il23* | *p*=0.0195 | WT *n*=5, *Cd93*$^{-/-}$ *n*=4 |
| *Il36g* | *p*=0.0008 | WT *n*=5, *Cd93*$^{-/-}$ *n*=4 |
| **Extended Data Fig.5d** | | |
| NC vs siCD93 | *p*=0.7207 | NC *n*=3, siCD93 *n*=3 |
| Con vs IL-17D | *p*=0.0075 | Con *n*=3, IL-17D *n*=3 |
| IL-17D vs IL-17D+siCD93 | *p*=0.0136 | IL-17D *n*=3, IL-17D+siCD93 *n*=3 |
| siCD93 vs IL-17D+siCD93 | *p*= 0.9292 | siCD93 *n*=3, IL-17D+siCD93 *n*=3 |
| **Extended Data Fig.6a** | | |
| *CCL20* | | |
| IL-17A 0h | *p*=0.9738 | WT *n*=3, *DDX5*$^{-/-}$ *n*=3 |
| IL-17A 2h | *p*=0.0197 | WT *n*=3, *DDX5*$^{-/-}$ *n*=3 |
| IL-17A 4h | *p*>0.9999 | WT *n*=3, *DDX5*$^{-/-}$ *n*=3 |
| IL-17A 6h | *p*=0.2933 | WT *n*=3, *DDX5*$^{-/-}$ *n*=3 |
| IL-17A 8h | *p*=0.2269 | WT *n*=3, *DDX5*$^{-/-}$ *n*=3 |
| IL-17A 10h | *p*>0.9999 | WT *n*=3, *DDX5*$^{-/-}$ *n*=3 |
| *CXCL1* | | |
| IL-17A 0h | *p*=0.9982 | WT *n*=3, *DDX5*$^{-/-}$ *n*=3 |
| IL-17A 2h | *p*<0.0001 | WT *n*=3, *DDX5*$^{-/-}$ *n*=3 |
| IL-17A 4h | *p*=0.1554 | WT *n*=3, *DDX5*$^{-/-}$ *n*=3 |
| IL-17A 6h | *p*=0.5333 | WT *n*=3, *DDX5*$^{-/-}$ *n*=3 |
| IL-17A 8h | *p*=0.8002 | WT *n*=3, *DDX5*$^{-/-}$ *n*=3 |
| IL-17A 10h | *p*=0.9785 | WT *n*=3, *DDX5*$^{-/-}$ *n*=3 |
| *CXCL2* | | |
| IL-17A 0h | *p*=0.5146 | WT *n*=3, *DDX5*$^{-/-}$ *n*=3 |
| IL-17A 2h | *p*<0.0001 | WT *n*=3, *DDX5*$^{-/-}$ *n*=3 |
| IL-17A 4h | *p*<0.0001 | WT *n*=3, *DDX5*$^{-/-}$ *n*=3 |
| IL-17A 6h | *p*>0.9999 | WT *n*=3, *DDX5*$^{-/-}$ *n*=3 |
| IL-17A 8h | *p*=0.9952 | WT *n*=3, *DDX5*$^{-/-}$ *n*=3 |
| IL-17A 10h | *p*=0.7198 | WT *n*=3, *DDX5*$^{-/-}$ *n*=3 |
| *CXCL6* | | |
| IL-17A 0h | *p*= 0.5895 | WT *n*=3, *DDX5*$^{-/-}$ *n*=3 |
| IL-17A 2h | *p*=0.9998 | WT *n*=3, *DDX5*$^{-/-}$ *n*=3 |
| IL-17A 4h | *p*=0.9358 | WT *n*=3, *DDX5*$^{-/-}$ *n*=3 |
| IL-17A 6h | *p*=0.3523 | WT *n*=3, *DDX5*$^{-/-}$ *n*=3 |
| IL-17A 8h | *p*=0.9831 | WT *n*=3, *DDX5*$^{-/-}$ *n*=3 |
| IL-17A 10h | *p*<0.0001 | WT *n*=3, *DDX5*$^{-/-}$ *n*=3 |

| | | |
|---|---|---|
| *CCL3* | | |
| IL-17A 0h | *p*=0.0773 | WT *n*=3, *DDX5*$^{-/-}$ *n*=3 |
| IL-17A 2h | *p*=0.1303 | WT *n*=3, *DDX5*$^{-/-}$ *n*=3 |
| IL-17A 4h | *p*=0.9993 | WT *n*=3, *DDX5*$^{-/-}$ *n*=3 |
| IL-17A 6h | *p*>0.9999 | WT *n*=3, *DDX5*$^{-/-}$ *n*=3 |
| IL-17A 8h | *p*=0.0231 | WT *n*=3, *DDX5*$^{-/-}$ *n*=3 |
| IL-17A 10h | *p*=0.1852 | WT *n*=3, *DDX5*$^{-/-}$ *n*=3 |
| *CCL17* | | |
| IL-17A 0h | *p*=0.0001 | WT *n*=3, *DDX5*$^{-/-}$ *n*=3 |
| IL-17A 2h | *p*<0.0001 | WT *n*=3, *DDX5*$^{-/-}$ *n*=3 |
| IL-17A 4h | *p*<0.0001 | WT *n*=3, *DDX5*$^{-/-}$ *n*=3 |
| IL-17A 6h | *p*<0.0001 | WT *n*=3, *DDX5*$^{-/-}$ *n*=3 |
| IL-17A 8h | *p*<0.0001 | WT *n*=3, *DDX5*$^{-/-}$ *n*=3 |
| IL-17A 10h | *p*<0.0001 | WT *n*=3, *DDX5*$^{-/-}$ *n*=3 |
| *CCL22* | | |
| IL-17A 0h | *p*=0.0077 | WT n=3, *DDX5*$^{-/-}$ n=3 |
| IL-17A 2h | *p*<0.0001 | WT n=3, *DDX5*$^{-/-}$ n=3 |
| IL-17A 4h | *p*=0.7368 | WT n=3, *DDX5*$^{-/-}$ n=3 |
| IL-17A 6h | *p*=0.4774 | WT n=3, *DDX5*$^{-/-}$ n=3 |
| IL-17A 8h | *p*=0.4204 | WT n=3, *DDX5*$^{-/-}$ n=3 |
| IL-17A 10h | *p*=0.0036 | WT n=3, *DDX5*$^{-/-}$ n=3 |
| **Extended Data Fig.6b** | | |
| *CCL20* | | |
| IL-36γ 0h | *p*=0.1678 | WT *n*=3, *DDX5*$^{-/-}$ *n*=3 |
| IL-36γ 2h | *p*<0.0001 | WT *n*=3, *DDX5*$^{-/-}$ *n*=3 |
| IL-36γ 4h | *p*<0.0001 | WT *n*=3, *DDX5*$^{-/-}$ *n*=3 |
| IL-36γ 6h | *p*<0.0001 | WT *n*=3, *DDX5*$^{-/-}$ *n*=3 |
| IL-36γ 8h | *p*<0.0001 | WT *n*=3, *DDX5*$^{-/-}$ *n*=3 |
| IL-36γ 10h | *p*<0.0001 | WT *n*=3, *DDX5*$^{-/-}$ *n*=3 |
| *CXCL1* | | |
| IL-36γ 0h | *p*=0.2393 | WT *n*=3, *DDX5*$^{-/-}$ *n*=3 |
| IL-36γ 2h | *p*<0.0001 | WT *n*=3, *DDX5*$^{-/-}$ *n*=3 |
| IL-36γ 4h | *p*<0.0001 | WT *n*=3, *DDX5*$^{-/-}$ *n*=3 |
| IL-36γ 6h | *p*<0.0001 | WT *n*=3, *DDX5*$^{-/-}$ *n*=3 |
| IL-36γ 8h | *p*<0.0001 | WT *n*=3, *DDX5*$^{-/-}$ *n*=3 |
| IL-36γ 10h | *p*=0.0010 | WT *n*=3, *DDX5*$^{-/-}$ *n*=3 |
| *CXCL2* | | |
| IL-36γ 0h | *p*=0.0288 | WT *n*=3, *DDX5*$^{-/-}$ *n*=3 |
| IL-36γ 2h | *p*<0.0001 | WT *n*=3, *DDX5*$^{-/-}$ *n*=3 |
| IL-36γ 4h | *p*<0.0001 | WT *n*=3, *DDX5*$^{-/-}$ *n*=3 |
| IL-36γ 6h | *p*<0.0001 | WT *n*=3, *DDX5*$^{-/-}$ *n*=3 |
| IL-36γ 8h | *p*<0.0001 | WT *n*=3, *DDX5*$^{-/-}$ *n*=3 |
| IL-36γ 10h | *p*<0.0001 | WT *n*=3, *DDX5*$^{-/-}$ *n*=3 |

| | | |
|---|---|---|
| *CXCL6* | | |
| IL-36γ 0h | $p<0.0001$ | WT $n$=3, $DDX5^{-/-}$ $n$=3 |
| IL-36γ 2h | $p<0.0001$ | WT $n$=3, $DDX5^{-/-}$ $n$=3 |
| IL-36γ 4h | $p<0.0001$ | WT $n$=3, $DDX5^{-/-}$ $n$=3 |
| IL-36γ 6h | $p<0.0001$ | WT $n$=3, $DDX5^{-/-}$ $n$=3 |
| IL-36γ 8h | $p<0.0001$ | WT $n$=3, $DDX5^{-/-}$ $n$=3 |
| IL-36γ 10h | $p<0.0001$ | WT $n$=3, $DDX5^{-/-}$ $n$=3 |
| *CCL3* | | |
| IL-36γ 0h | $p<0.0001$ | WT $n$=3, $DDX5^{-/-}$ $n$=3 |
| IL-36γ 2h | $p<0.0001$ | WT $n$=3, $DDX5^{-/-}$ $n$=3 |
| IL-36γ 4h | $p<0.0001$ | WT $n$=3, $DDX5^{-/-}$ $n$=3 |
| IL-36γ 6h | $p<0.0001$ | WT $n$=3, $DDX5^{-/-}$ $n$=3 |
| IL-36γ 8h | $p<0.0001$ | WT $n$=3, $DDX5^{-/-}$ $n$=3 |
| IL-36γ 10h | $p<0.0001$ | WT $n$=3, $DDX5^{-/-}$ $n$=3 |
| *CCL17* | | |
| IL-36γ 0h | $p=0.0001$ | WT $n$=3, $DDX5^{-/-}$ $n$=3 |
| IL-36γ 2h | $p<0.0001$ | WT $n$=3, $DDX5^{-/-}$ $n$=3 |
| IL-36γ 4h | $p<0.0001$ | WT $n$=3, $DDX5^{-/-}$ $n$=3 |
| IL-36γ 6h | $p<0.0001$ | WT $n$=3, $DDX5^{-/-}$ $n$=3 |
| IL-36γ 8h | $p=0.0005$ | WT $n$=3, $DDX5^{-/-}$ $n$=3 |
| IL-36γ 10h | $p<0.0001$ | WT $n$=3, $DDX5^{-/-}$ $n$=3 |
| *CCL22* | | |
| IL-36γ 0h | $p<0.0001$ | WT $n$=3, $DDX5^{-/-}$ $n$=3 |
| IL-36γ 2h | $p<0.0001$ | WT $n$=3, $DDX5^{-/-}$ $n$=3 |
| IL-36γ 4h | $p<0.0001$ | WT $n$=3, $DDX5^{-/-}$ $n$=3 |
| IL-36γ 6h | $p<0.0001$ | WT $n$=3, $DDX5^{-/-}$ $n$=3 |
| IL-36γ 8h | $p=0.1585$ | WT $n$=3, $DDX5^{-/-}$ $n$=3 |
| IL-36γ 10h | $p<0.0001$ | WT $n$=3, $DDX5^{-/-}$ $n$=3 |
| **Extended Data Fig.6c** | | |
| *CCL20* | | |
| TNF 0h | $p$=>0.9999 | WT $n$=3, $DDX5^{-/-}$ $n$=3 |
| TNF 2h | $p<0.0001$ | WT $n$=3, $DDX5^{-/-}$ $n$=3 |
| TNF 4h | $p=0.6848$ | WT $n$=3, $DDX5^{-/-}$ $n$=3 |
| TNF 6h | $p=0.9913$ | WT $n$=3, $DDX5^{-/-}$ $n$=3 |
| TNF 8h | $p=0.9997$ | WT $n$=3, $DDX5^{-/-}$ $n$=3 |
| TNF 10h | $p=0.9915$ | WT $n$=3, $DDX5^{-/-}$ $n$=3 |
| *CXCL1* | | |
| TNF 0h | $p>0.9999$ | WT $n$=3, $DDX5^{-/-}$ $n$=3 |
| TNF 2h | $p=0.9342$ | WT $n$=3, $DDX5^{-/-}$ $n$=3 |
| TNF 4h | $p=0.0029$ | WT $n$=3, $DDX5^{-/-}$ $n$=3 |
| TNF 6h | $p=0.0640$ | WT $n$=3, $DDX5^{-/-}$ $n$=3 |
| TNF 8h | $p=0.0078$ | WT $n$=3, $DDX5^{-/-}$ $n$=3 |
| TNF 10h | $p=0.2654$ | WT $n$=3, $DDX5^{-/-}$ $n$=3 |

| | | |
|---|---|---|
| *CXCL2* | | |
| TNF 0h | *p*=0.9476 | WT *n*=3, *DDX5*$^{-/-}$ *n*=3 |
| TNF 2h | *p*<0.0001 | WT *n*=3, *DDX5*$^{-/-}$ *n*=3 |
| TNF 4h | *p*=0.0023 | WT *n*=3, *DDX5*$^{-/-}$ *n*=3 |
| TNF 6h | *p*=0.0399 | WT *n*=3, *DDX5*$^{-/-}$ *n*=3 |
| TNF 8h | *p*=0.2822 | WT *n*=3, *DDX5*$^{-/-}$ *n*=3 |
| TNF 10h | *p*=0.0480 | WT *n*=3, *DDX5*$^{-/-}$ *n*=3 |
| *CXCL6* | | |
| TNF 0h | *p*=0.1220 | WT *n*=3, *DDX5*$^{-/-}$ *n*=3 |
| TNF 2h | *p*<0.0001 | WT *n*=3, *DDX5*$^{-/-}$ *n*=3 |
| TNF 4h | *p*<0.0001 | WT *n*=3, *DDX5*$^{-/-}$ *n*=3 |
| TNF 6h | *p*<0.0001 | WT *n*=3, *DDX5*$^{-/-}$ *n*=3 |
| TNF 8h | *p*<0.0001 | WT *n*=3, *DDX5*$^{-/-}$ *n*=3 |
| TNF 10h | *p*=0.0002 | WT *n*=3, *DDX5*$^{-/-}$ *n*=3 |
| *CCL3* | | |
| TNF 0h | *p*<0.0001 | WT *n*=3, *DDX5*$^{-/-}$ *n*=3 |
| TNF 2h | *p*<0.0001 | WT *n*=3, *DDX5*$^{-/-}$ *n*=3 |
| TNF 4h | *p*<0.0001 | WT *n*=3, *DDX5*$^{-/-}$ *n*=3 |
| TNF 6h | *p*<0.0001 | WT *n*=3, *DDX5*$^{-/-}$ *n*=3 |
| TNF 8h | *p*<0.0001 | WT *n*=3, *DDX5*$^{-/-}$ *n*=3 |
| TNF 10h | *p*<0.0001 | WT *n*=3, *DDX5*$^{-/-}$ *n*=3 |
| *CCL17* | | |
| TNF 0h | *p*=0.2165 | WT *n*=3, *DDX5*$^{-/-}$ *n*=3 |
| TNF 2h | *p*<0.0001 | WT *n*=3, *DDX5*$^{-/-}$ *n*=3 |
| TNF 4h | *p*<0.0001 | WT *n*=3, *DDX5*$^{-/-}$ *n*=3 |
| TNF 6h | *p*<0.0001 | WT *n*=3, *DDX5*$^{-/-}$ *n*=3 |
| TNF 8h | *p*<0.0001 | WT *n*=3, *DDX5*$^{-/-}$ *n*=3 |
| TNF 10h | *P*=0.0416 | WT *n*=3, *DDX5*$^{-/-}$ *n*=3 |
| *CCL22* | | |
| TNF 0h | *p*=0.0911 | WT *n*=3, *DDX5*$^{-/-}$ *n*=3 |
| TNF 2h | *p*<0.0001 | WT *n*=3, *DDX5*$^{-/-}$ *n*=3 |
| TNF 4h | *p*<0.0001 | WT *n*=3, *DDX5*$^{-/-}$ *n*=3 |
| TNF 6h | *p*<0.0001 | WT *n*=3, *DDX5*$^{-/-}$ *n*=3 |
| TNF 8h | *p*<0.0001 | WT *n*=3, *DDX5*$^{-/-}$ *n*=3 |
| TNF 10h | *p*=0.0009 | WT *n*=3, *DDX5*$^{-/-}$ *n*=3 |
| **Extended Data Fig.6d** | | |
| *CCL20* | | |
| IL-25 0h | *p*<0.0001 | WT *n*=3, *DDX5*$^{-/-}$ *n*=3 |
| IL-25 2h | *p*<0.0001 | WT *n*=3, *DDX5*$^{-/-}$ *n*=3 |
| IL-25 4h | *p*<0.0001 | WT *n*=3, *DDX5*$^{-/-}$ *n*=3 |
| IL-25 6h | *p*<0.0001 | WT *n*=3, *DDX5*$^{-/-}$ *n*=3 |
| IL-25 8h | *p*<0.0001 | WT *n*=3, *DDX5*$^{-/-}$ *n*=3 |
| IL-25 10h | *p*<0.0001 | WT *n*=3, *DDX5*$^{-/-}$ *n*=3 |

| | | |
|---|---|---|
| *CXCL1* | | |
| IL-25 0h | $p<0.0001$ | WT $n=3$, $DDX5^{-/-}$ $n=3$ |
| IL-25 2h | $p<0.0001$ | WT $n=3$, $DDX5^{-/-}$ $n=3$ |
| IL-25 4h | $p<0.0001$ | WT $n=3$, $DDX5^{-/-}$ $n=3$ |
| IL-25 6h | $p<0.0001$ | WT $n=3$, $DDX5^{-/-}$ $n=3$ |
| IL-25 8h | $p<0.0001$ | WT $n=3$, $DDX5^{-/-}$ $n=3$ |
| IL-25 10h | $p<0.0001$ | WT $n=3$, $DDX5^{-/-}$ $n=3$ |
| *CXCL2* | | |
| IL-25 0h | $P=0.0067$ | WT $n=3$, $DDX5^{-/-}$ $n=3$ |
| IL-25 2h | $p<0.0001$ | WT $n=3$, $DDX5^{-/-}$ $n=3$ |
| IL-25 4h | $p<0.0001$ | WT $n=3$, $DDX5^{-/-}$ $n=3$ |
| IL-25 6h | $p<0.0001$ | WT $n=3$, $DDX5^{-/-}$ $n=3$ |
| IL-25 8h | $p<0.0001$ | WT $n=3$, $DDX5^{-/-}$ $n=3$ |
| IL-25 10h | $p<0.0001$ | WT $n=3$, $DDX5^{-/-}$ $n=3$ |
| *CXCL6* | | |
| IL-25 0h | $P=0.0067$ | WT $n=3$, $DDX5^{-/-}$ $n=3$ |
| IL-25 2h | $p<0.0001$ | WT $n=3$, $DDX5^{-/-}$ $n=3$ |
| IL-25 4h | $p<0.0001$ | WT $n=3$, $DDX5^{-/-}$ $n=3$ |
| IL-25 6h | $P=0.0133$ | WT $n=3$, $DDX5^{-/-}$ $n=3$ |
| IL-25 8h | $p<0.0001$ | WT $n=3$, $DDX5^{-/-}$ $n=3$ |
| IL-25 10h | $P=0.0004$ | WT $n=3$, $DDX5^{-/-}$ $n=3$ |
| *CCL3* | | |
| IL-25 0h | $p=<0.0001$ | WT $n=3$, $DDX5^{-/-}$ $n=3$ |
| IL-25 2h | $p=0.0004$ | WT $n=3$, $DDX5^{-/-}$ $n=3$ |
| IL-25 4h | $p=0.0765$ | WT $n=3$, $DDX5^{-/-}$ $n=3$ |
| IL-25 6h | $p=0.9980$ | WT $n=3$, $DDX5^{-/-}$ $n=3$ |
| IL-25 8h | $p=0.9988$ | WT $n=3$, $DDX5^{-/-}$ $n=3$ |
| IL-25 10h | $p=0.0014$ | WT $n=3$, $DDX5^{-/-}$ $n=3$ |
| *CCL17* | | |
| IL-25 0h | $p<0.0001$ | WT $n=3$, $DDX5^{-/-}$ $n=3$ |
| IL-25 2h | $p=0.9382$ | WT $n=3$, $DDX5^{-/-}$ $n=3$ |
| IL-25 4h | $p=0.0015$ | WT $n=3$, $DDX5^{-/-}$ $n=3$ |
| IL-25 6h | $p<0.0001$ | WT $n=3$, $DDX5^{-/-}$ $n=3$ |
| IL-25 8h | $p=0.0033$ | WT $n=3$, $DDX5^{-/-}$ $n=3$ |
| IL-25 10h | $p<0.0001$ | WT $n=3$, $DDX5^{-/-}$ $n=3$ |
| *CCL22* | | |
| IL-25 0h | $p=0.9998$ | WT $n=3$, $DDX5^{-/-}$ $n=3$ |
| IL-25 2h | $p=0.1486$ | WT $n=3$, $DDX5^{-/-}$ $n=3$ |
| IL-25 4h | $p=0.5403$ | WT $n=3$, $DDX5^{-/-}$ $n=3$ |
| IL-25 6h | $p=0.1091$ | WT $n=3$, $DDX5^{-/-}$ $n=3$ |
| IL-25 8h | $p=0.8866$ | WT $n=3$, $DDX5^{-/-}$ $n=3$ |
| IL-25 10h | $p=0.5127$ | WT $n=3$, $DDX5^{-/-}$ $n=3$ |
| **Extended Data Fig.6e** | | |

| | | |
|---|---|---|
| *CCL20* | | |
| IL-4 0h | $p$<0.0001 | WT $n$=3, $DDX5^{-/-}$ $n$=3 |
| IL-4 2h | $p$<0.0001 | WT $n$=3, $DDX5^{-/-}$ $n$=3 |
| IL-4 4h | $p$<0.0001 | WT $n$=3, $DDX5^{-/-}$ $n$=3 |
| IL-4 6h | $p$<0.0001 | WT $n$=3, $DDX5^{-/-}$ $n$=3 |
| IL-4 8h | $p$<0.0001 | WT $n$=3, $DDX5^{-/-}$ $n$=3 |
| IL-4 10h | $p$<0.0001 | WT $n$=3, $DDX5^{-/-}$ $n$=3 |
| *CXCL1* | | |
| IL-4 0h | $p$=0.0013 | WT $n$=3, $DDX5^{-/-}$ $n$=3 |
| IL-4 2h | $p$=0.0778 | WT $n$=3, $DDX5^{-/-}$ $n$=3 |
| IL-4 4h | $p$=0.6361 | WT $n$=3, $DDX5^{-/-}$ $n$=3 |
| IL-4 6h | $p$=0.0794 | WT $n$=3, $DDX5^{-/-}$ $n$=3 |
| IL-4 8h | $p$=0.0048 | WT $n$=3, $DDX5^{-/-}$ $n$=3 |
| IL-4 10h | $p$=0.0299 | WT $n$=3, $DDX5^{-/-}$ $n$=3 |
| *CXCL2* | | |
| IL-4 0h | $p$=0.8912 | WT $n$=3, $DDX5^{-/-}$ $n$=3 |
| IL-4 2h | $p$=0.7548 | WT $n$=3, $DDX5^{-/-}$ $n$=3 |
| IL-4 4h | $p$=0.7548 | WT $n$=3, $DDX5^{-/-}$ $n$=3 |
| IL-4 6h | $p$=0.0014 | WT $n$=3, $DDX5^{-/-}$ $n$=3 |
| IL-4 8h | $p$<0.0001 | WT $n$=3, $DDX5^{-/-}$ $n$=3 |
| IL-4 10h | $p$=0.0187 | WT $n$=3, $DDX5^{-/-}$ $n$=3 |
| *CXCL6* | | |
| IL-4 0h | $p$=0.9992 | WT $n$=3, $DDX5^{-/-}$ $n$=3 |
| IL-4 2h | $p$=0.5598 | WT $n$=3, $DDX5^{-/-}$ $n$=3 |
| IL-4 4h | $p$=0.1821 | WT $n$=3, $DDX5^{-/-}$ $n$=3 |
| IL-4 6h | $p$=0.0035 | WT $n$=3, $DDX5^{-/-}$ $n$=3 |
| IL-4 8h | $p$=0.4617 | WT $n$=3, $DDX5^{-/-}$ $n$=3 |
| IL-4 10h | $p$=0.0181 | WT $n$=3, $DDX5^{-/-}$ $n$=3 |
| *CCL3* | | |
| IL-4 0h | $p$=<0.0001 | WT $n$=3, $DDX5^{-/-}$ $n$=3 |
| IL-4 2h | $p$=0.0875 | WT $n$=3, $DDX5^{-/-}$ $n$=3 |
| IL-4 4h | $p$=0.0744 | WT $n$=3, $DDX5^{-/-}$ $n$=3 |
| IL-4 6h | $p$=0.0485 | WT $n$=3, $DDX5^{-/-}$ $n$=3 |
| IL-4 8h | $p$=0.9992 | WT $n$=3, $DDX5^{-/-}$ $n$=3 |
| IL-4 10h | $p$=0.0015 | WT $n$=3, $DDX5^{-/-}$ $n$=3 |
| *CCL17* | | |
| IL-4 0h | $p$<0.0001 | WT $n$=3, $DDX5^{-/-}$ $n$=3 |
| IL-4 2h | $p$<0.0001 | WT $n$=3, $DDX5^{-/-}$ $n$=3 |
| IL-4 4h | $p$<0.0001 | WT $n$=3, $DDX5^{-/-}$ $n$=3 |
| IL-4 6h | $p$<0.0001 | WT $n$=3, $DDX5^{-/-}$ $n$=3 |
| IL-4 8h | $p$<0.0001 | WT $n$=3, $DDX5^{-/-}$ $n$=3 |
| IL-4 10h | $p$<0.0001 | WT $n$=3, $DDX5^{-/-}$ $n$=3 |
| *CCL22* | | |

| | | | |
|---|---|---|---|
| IL-4 0h | | $p<0.0001$ | WT $n=3$, *DDX5*$^{-/-}$ $n=3$ |
| IL-4 2h | | $p<0.0001$ | WT $n=3$, *DDX5*$^{-/-}$ $n=3$ |
| IL-4 4h | | $p<0.0001$ | WT $n=3$, *DDX5*$^{-/-}$ $n=3$ |
| IL-4 6h | | $p<0.0001$ | WT $n=3$, *DDX5*$^{-/-}$ $n=3$ |
| IL-4 8h | | $p<0.0001$ | WT $n=3$, *DDX5*$^{-/-}$ $n=3$ |
| IL-4 10h | | $p=0.0244$ | WT $n=3$, *DDX5*$^{-/-}$ $n=3$ |
| **Extended Data Fig. 6h** | | | |
| *IL36RN* | WT vs *DDX5*$^{-/-}$ | $p=0.1555$ | $n=3$ |
| *IL38* | WT vs *DDX5*$^{-/-}$ | $p=0.9181$ | $n=3$ |
| **Extended Data Fig. 6i** | | | |
| *IL36RN* | NC vs siDDX5 | $p=0.1910$ | $n=3$ |
| *IL38* | NC vs siDDX5 | $p=0.4461$ | $n=3$ |
| **Extended Data Fig. 7j** | | | |
| *DDX1* | NC vs siDDX1 | $p=0.0991$ | $n=3$ |
| *DDX3* | NC vs siDDX3 | $p=0.2332$ | $n=3$ |
| *DDX5* | NC vs siDDX5 | $p=0.1648$ | $n=3$ |
| *DDX17* | NC vs siDDX17 | $p=0.0013$ | $n=3$ |
| *DDX23* | NC vs siDDX23 | $p=0.1056$ | $n=3$ |
| *DDX39* | NC vs siDDX39 | $p=0.2526$ | $n=3$ |
| *DDX41* | NC vs siDDX41 | $p=0.4116$ | $n=3$ |
| *DDX42* | NC vs siDDX42 | $p=0.4508$ | $n=3$ |
| *DDX46* | NC vs siDDX46 | $p=0.0048$ | $n=3$ |
| *DDX48* | NC vs siDDX48 | $p=0.4021$ | $n=3$ |
| **Extended Data Fig. 8d** | | | |
| *CCL20* | 0h:NC vs 0h:siSF2 | $p>0.9999$ | $n=3$ |
| | 1h:NC vs 1h:siSF2 | $p=0.4536$ | $n=3$ |
| | 3h:NC vs 3h:siSF2 | $p<0.0001$ | $n=3$ |
| | 5h:NC vs 5h:siSF2 | $p<0.0001$ | $n=3$ |
| | 7h:NC vs 7h:siSF2 | $p<0.0001$ | $n=3$ |
| *CXCL1* | 0h:NC vs 0h:siSF2 | $p>0.9999$ | $n=3$ |
| | 1h:NC vs 1h:siSF2 | $p=0.9485$ | $n=3$ |
| | 3h:NC vs 3h:siSF2 | $p=0.0002$ | $n=3$ |
| | 5h:NC vs 5h:siSF2 | $p<0.0001$ | $n=3$ |
| | 7h:NC vs 7h:siSF2 | $p=0.2714$ | $n=3$ |
| *CXCL6* | 0h:NC vs 0h:siSF2 | $p>0.9999$ | $n=3$ |
| | 1h:NC vs 1h:siSF2 | $p>0.9999$ | $n=3$ |
| | 3h:NC vs 3h:siSF2 | $p=0.0662$ | $n=3$ |
| | 5h:NC vs 5h:siSF2 | $p<0.0001$ | $n=3$ |
| | 7h:NC vs 7h:siSF2 | $p<0.0001$ | $n=3$ |
| *CCL17* | 0h:NC vs 0h:siSF2 | $p=0.8831$ | $n=3$ |
| | 1h:NC vs 1h:siSF2 | $p<0.0001$ | $n=3$ |
| | 3h:NC vs 3h:siSF2 | $p=0.0011$ | $n=3$ |
| | 5h:NC vs 5h:siSF2 | $p<0.0001$ | $n=3$ |

| | | | |
|---|---|---|---|
| | 7h:NC vs 7h:siSF2 | *p*=0.0002 | *n*=3 |
| *CCL3* | 0h:NC vs 0h:siSF2 | *p*=0.1007 | *n*=3 |
| | 1h:NC vs 1h:siSF2 | *p*<0.0001 | *n*=3 |
| | 3h:NC vs 3h:siSF2 | *p*<0.0001 | *n*=3 |
| | 5h:NC vs 5h:siSF2 | *p*<0.0001 | *n*=3 |
| | 7h:NC vs 7h:siSF2 | *p*<0.0001 | *n*=3 |
| *CCL11* | 0h:NC vs 0h:siSF2 | *p*=0.9995 | *n*=3 |
| | 1h:NC vs 1h:siSF2 | *p*=0.9750 | *n*=3 |
| | 3h:NC vs 3h:siSF2 | *p*=0.0002 | *n*=3 |
| | 5h:NC vs 5h:siSF2 | *p*=0.0212 | *n*=3 |
| | 7h:NC vs 7h:siSF2 | *p*> 0.9999 | *n*=3 |
| *CCL27* | 0h:NC vs 0h:siSF2 | *p*=0.9998 | *n*=3 |
| | 1h:NC vs 1h:siSF2 | *p*=0.3194 | *n*=3 |
| | 3h:NC vs 3h:siSF2 | *p*=0.1597 | *n*=3 |
| | 5h:NC vs 5h:siSF2 | *p*=0.0631 | *n*=3 |
| | 7h:NC vs 7h:siSF2 | *p*=0.0483 | *n*=3 |

| | | |
|---|---|---|
| **Extended Data Fig. 9a** | | |
| PBS *vs* sIL-36R | *p*=0.0004 | PBS *n*=6, sIL-36R *n*=6 |
| **Extended Data Fig. 9b** | | |
| *CCL20* | | |
| pcmv:NC vs pcmv:siDDX5 | *p*=0.0796 | NC *n*=3, siDDX5 *n*=3 |
| pcmv:NC vs sIL36R:NC | *p* >0.9999 | NC *n*=3, siDDX5 *n*=3 |
| pcmv:NC vs sIL36R:siDDX5 | *p*=0.0071 | NC *n*=3, siDDX5 *n*=3 |
| pcmv:NC vs sIL36R+IL36γ:NC | *p*<0.0001 | NC *n*=3, siDDX5 *n*=3 |
| pcmv:NC vs pcmv+IL36γ:siDDX5 | *p*=0.9397 | NC *n*=3, siDDX5 *n*=3 |
| pcmv:NC vs sIL36R+ IL36γ:NC | *p*=0.0023 | NC *n*=3, siDDX5 *n*=3 |
| pcmv:NC vs sIL36R + IL36γ:siDDX5 | *p*=0.0475 | NC *n*=3, siDDX5 *n*=3 |
| pcmv:siDDX5 vs sIL36R:NC | *p*=0.8916 | NC *n*=3, siDDX5 *n*=3 |
| pcmv:siDDX5vs sIL36R:siDDX5 | *p*=0.9001 | NC *n*=3, siDDX5 *n*=3 |
| pcmv:siDDX5 vs pcmv+ IL36γ:NC | *p*<0.0001 | NC *n*=3, siDDX5 *n*=3 |
| pcmv:siDDX5 vs pcmv+ IL36γ:siDDX5 | *p*=0.4666 | NC *n*=3, siDDX5 *n*=3 |
| pcmv:siDDX5 vs sIL-36R+ IL36γ:NC | *p*=0.6027 | NC n=3, siDDX5 n=3 |
| pcmv:siDDX5 vs IL36γ:siDDX5 | *p*=0.3982 | NC *n*=3, siDDX5 *n*=3 |
| sIL36R:NC vs sIL36R:siDDX5 | *p*=0.0041 | NC *n*=3, siDDX5 *n*=3 |
| sIL36R:NC vs pcmv+IL36γ:NC | *p*<0.0001 | NC *n*=3, siDDX5 *n*=3 |
| sIL36R:NC vs pcmv+ IL36γ:siDDX5 | *p*=0.8393 | NC *n*=3, siDDX5 *n*=3 |
| sIL36R:NC vs sIL36R+IL36γ:NC | *p*=0.0013 | NC *n*=3, siDDX5 *n*=3 |
| sIL36R:NC vs sIL36R + IL36γ:siDDX5 | *p*=0.2441 | NC *n*=3, siDDX5 *n*=3 |
| sIL36R:siDDX5 vs pcmv+IL36γ:NC | *p*<0.0001 | NC *n*=3, siDDX5 *n*=3 |
| sIL36R:siDDX5 vs pcmv+ IL36γ:siDDX5 | *p*=0.9912 | NC *n*=3, siDDX5 *n*=3 |

| | | |
|---|---|---|
| sIL36R:siDDX5 vs sIL36R+IL36g:NC | *p*=0.0921 | NC *n*=3, siDDX5 *n*=3 |
| sIL36R:siDDX5 vs sIL36R+ IL36γ:siDDX5 | *p*<0.0001 | NC *n*=3, siDDX5 *n*=3 |
| pcmv+IL36γ:NC vs pcmv+ IL36γ:siDDX5 | *p*=<0.0001 | NC *n*=3, siDDX5 *n*=3 |
| pcmv+ IL36γ:NC vs sIL36R + IL36γ:NC | *p*=0.0628 | NC *n*=3, siDDX5 *n*=3 |
| pcmv+IL36γ:NC vssIL36R+IL36γ:siDDX5 | *p*=0.9987 | NC *n*=3, siDDX5 *n*=3 |
| pcmv+IL36γ:siDDX5 vs sIL36R +IL36γ:NC | *p*<0.0001 | NC n=3, siDDX5 n=3 |
| pcmv+IL36γ:siDDX5 vs sIL36R+ IL36γ:siDDX5 | *p*<0.0001 | NC *n*=3, siDDX5 *n*=3 |
| sIL36R+IL36γ:NC vs sIL36R+ IL36γ:siDDX5 | *p*=0.0208 | NC *n*=3, siDDX5 *n*=3 |
| *CXCL1* | | |
| pcmv:NC vs pcmv:siDDX5 | *p* <0.0001 | NC *n*=3, siDDX5 *n*=3 |
| pcmv:NC vs sIL36R:NC | *p* <0.0001 | NC *n*=3, siDDX5 *n*=3 |
| pcmv:NC vs sIL36R:siDDX5 | *p* >0.9999 | NC *n*=3, siDDX5 *n*=3 |
| pcmv:NC vs sIL36R+IL36γ:NC | *p*=0.0020 | NC *n*=3, siDDX5 *n*=3 |
| pcmv:NC vs pcmv+IL36γ:siDDX5 | *p*=0.2535 | NC *n*=3, siDDX5 *n*=3 |
| pcmv:NC vs sIL36R+IL36γ:NC | *p*<0.0001 | NC *n*=3, siDDX5 *n*=3 |
| pcmv:NC vs sIL36R +IL36γ:siDDX5 | *p*=0.6037 | NC *n*=3, siDDX5 *n*=3 |
| pcmv:siDDX5 vs sIL36R:NC | *p*<0.0001 | NC *n*=3, siDDX5 *n*=3 |
| pcmv:siDDX5vs sIL36R:siDDX5 | *p*<0.0001 | NC *n*=3, siDDX5 *n*=3 |
| pcmv:siDDX5 vs pcmv+IL36γ:NC | *p*=0.3056 | NC *n*=3, siDDX5 *n*=3 |
| pcmv:siDDX5 vs pcmv+IL-36γ:siDDX5 | *p*<0.0001 | NC *n*=3, siDDX5 *n*=3 |
| pcmv:siDDX5 vs sIL-36R+IL36γ:NC | *p*=0.0007 | NC *n*=3, siDDX5 *n*=3 |
| pcmv:siDDX5 vs IL-36γ:siDDX5 | *p*=0.3090 | NC *n*=3, siDDX5 *n*=3 |
| sIL36R:NC vs sIL36R:siDDX5 | *p*=0.0022 | NC *n*=3, siDDX5 *n*=3 |
| sIL36R:NC vs pcmv+IL36γ:NC | *p*=0.2759 | NC *n*=3, siDDX5 *n*=3 |
| sIL36R:NC vs pcmv+IL36γ:siDDX5 | *p*<0.0001 | NC *n*=3, siDDX5 *n*=3 |
| sIL36R:NC vs sIL36R+IL36γ:NC | *p*=0.6372 | NC *n*=3, siDDX5 *n*=3 |
| sIL36R:NC vs sIL36R +IL36γ:siDDX5 | *p*<0.0001 | NC *n*=3, siDDX5 *n*=3 |
| sIL36R:siDDX5 vs pcmv+IL36γ:NC | *p*=0.2229 | NC *n*=3, siDDX5 *n*=3 |
| sIL36R:siDDX5 vs pcmv+ IL36γ: siDDX5 | *p*<0.0001 | NC *n*=3, siDDX5 *n*=3 |
| sIL36R:siDDX5 vs sIL36R+IL36γ:NC | *p*=0.0692 | NC *n*=3, siDDX5 *n*=3 |
| sIL36R:siDDX5 vs sIL36R+IL36γ:siDDX5 | *p*=0.0039 | NC *n*=3, siDDX5 *n*=3 |
| pcmv+IL36γ:NC vs pcmv+ IL36γ:siDDX5 | *p*<0.0001 | NC n=3, siDDX5 n=3 |

| | | |
|---|---|---|
| pcmv+ IL36γ:NC vs sIL36R + IL36γ:NC | *p*=0.9964 | NC *n*=3, siDDX5 *n*=3 |
| pcmv+IL36γ:NC vs sIL36R+ IL36γ:siDDX5 | *p*<0.0001 | NC *n*=3, siDDX5 *n*=3 |
| pcmv+IL36γ:siDDX5 vs sIL36R+IL36γ:NC | *p*<0.0001 | NC *n*=3, siDDX5 *n*=3 |
| pcmv+IL36γ:siDDX5 vs sIL36R+ IL36γ:siDDX5 | *p*<0.0001 | NC *n*=3, siDDX5 *n*=3 |
| sIL36R+IL36γ:NC vs sIL36R+ IL36γ:siDDX5 | *p*<0.0001 | NC *n*=3, siDDX5 *n*=3 |
| *CXCL2* | | |
| pcmv:NC vs pcmv:siDDX5 | *p*= 0.6941 | NC *n*=3, siDDX5 *n*=3 |
| pcmv:NC vs sIL36R:NC | *p*=>0.9999 | NC *n*=3, siDDX5 *n*=3 |
| pcmv:NC vs sIL36R:siDDX5 | *p*=0.9832 | NC *n*=3, siDDX5 *n*=3 |
| pcmv:NC vs sIL36R+IL36γ:NC | *p*=0.0068 | NC *n*=3, siDDX5 *n*=3 |
| pcmv:NC vs pcmv+IL36γ:siDDX5 | *p*<0.0001 | NC *n*=3, siDDX5 *n*=3 |
| pcmv:NC vs sIL36R+IL36γ:NC | *p*<0.0001 | NC *n*=3, siDDX5 *n*=3 |
| pcmv:NC vs sIL36R +IL36γ:siDDX5 | *p*>0.9999 | NC *n*=3, siDDX5 *n*=3 |
| pcmv:siDDX5 vs sIL36R:NC | *p*=0.9771 | NC *n*=3, siDDX5 *n*=3 |
| pcmv:siDDX5vs sIL36R:siDDX5 | *p*=0.8415 | NC *n*=3, siDDX5 *n*=3 |
| pcmv:siDDX5 vs pcmv+IL36γ:NC | *p*=0.9905 | NC *n*=3, siDDX5 *n*=3 |
| pcmv:siDDX5 vs pcmv+IL-36γ:siDDX5 | *p*=0.1604 | NC *n*=3, siDDX5 *n*=3 |
| pcmv:siDDX5 vs sIL-36R+IL36γ:NC | *p*=0.0002 | NC *n*=3, siDDX5 *n*=3 |
| pcmv:siDDX5 vs IL-36γ:siDDX5 | *p*=0.8120 | NC n=3, siDDX5 n=3 |
| sIL36R:NC vs sIL36R:siDDX5 | *p*=0.9936 | NC *n*=3, siDDX5 *n*=3 |
| sIL36R:NC vs pcmv+IL36γ:NC | *p*= 0.0118 | NC *n*=3, siDDX5 *n*=3 |
| sIL36R:NC vs pcmv+IL36γ:siDDX5 | *p*<0.0001 | NC *n*=3, siDDX5 *n*=3 |
| sIL36R:NC vs sIL36R+IL36γ:NC | *p*=>0.9999 | NC *n*=3, siDDX5 *n*=3 |
| sIL36R:NC vs sIL36R +IL36γ:siDDX5 | *p*=0.9971 | NC *n*=3, siDDX5 *n*=3 |
| sIL36R:siDDX5 vs pcmv+IL36γ:NC | *p*=0.0378 | NC *n*=3, siDDX5 *n*=3 |
| sIL36R:siDDX5 vs pcmv+IL36γ:siDDX5 | *p*<0.0001 | NC *n*=3, siDDX5 *n*=3 |
| sIL36R:siDDX5 vs sIL36R+IL36γ:NC | *p*=0.9968 | NC *n*=3, siDDX5 *n*=3 |
| sIL36R:siDDX5 vs sIL36R+IL36γ:siDDX5 | *p*>0.9999 | NC *n*=3, siDDX5 *n*=3 |
| pcmv+IL36γ:NC vs pcmv+IL36γ:siDDX5 | *p*=0.0413 | NC *n*=3, siDDX5 *n*=3 |
| pcmv+ IL36γ:NC vs sIL36R + IL36γ:NC | *p*=0.0104 | NC *n*=3, siDDX5 *n*=3 |
| pcmv+IL36γ:NC vs sIL36R+ IL36γ:siDDX5 | *p*=0.0417 | NC *n*=3, siDDX5 *n*=3 |
| pcmv+IL36γ:siDDX5 vs | *p*<0.0001 | NC *n*=3, siDDX5 *n*=3 |

| | | |
|---|---|---|
| sIL36R+IL36γ:NC | | |
| pcmv+IL36γ:siDDX5 vs sIL36R+ IL36γ:siDDX5 | *p*<0.0001 | NC *n*=3, siDDX5 *n*=3 |
| sIL36R+IL36γ:NC vs sIL36R+ IL36γ:siDDX5 | *p*=0.9950 | NC *n*=3, siDDX5 *n*=3 |
| *CXCL3* | | NC *n*=3, siDDX5 *n*=3 |
| pcmv:NC vs pcmv:siDDX5 | *p*=0.4013 | NC *n*=3, siDDX5 *n*=3 |
| pcmv:NC vs sIL36R:NC | *p*=>0.9999 | NC *n*=3, siDDX5 *n*=3 |
| pcmv:NC vs sIL36R:siDDX5 | *p*=0.9192 | NC n=3, siDDX5 n=3 |
| pcmv:NC vs sIL36R+IL36γ:NC | *p*<0.0001 | NC *n*=3, siDDX5 *n*=3 |
| pcmv:NC vs pcmv+IL36γ:siDDX5 | *p*<0.0001 | NC *n*=3, siDDX5 *n*=3 |
| pcmv:NC vs sIL36R+IL36γ:NC | *p*>0.9999 | NC *n*=3, siDDX5 *n*=3 |
| pcmv:NC vs sIL36R +IL36γ:siDDX5 | *p*=0.7218 | NC *n*=3, siDDX5 *n*=3 |
| pcmv:siDDX5 vs sIL36R:NC | *p*=0.4752 | NC *n*=3, siDDX5 *n*=3 |
| pcmv:siDDX5vs sIL36R:siDDX5 | *p*=0.9689 | NC n=3, siDDX5 n=3 |
| pcmv:siDDX5 vs pcmv+IL36γ:NC | *p*=0.0003 | NC *n*=3, siDDX5 *n*=3 |
| pcmv:siDDX5 vs pcmv+IL-36γ:siDDX5 | *p*<0.0001 | NC *n*=3, siDDX5 *n*=3 |
| pcmv:siDDX5 vs sIL-36R+IL36γ:NC | *p*=0.6190 | NC *n*=3, siDDX5 *n*=3 |
| pcmv:siDDX5 vs IL-36γ:siDDX5 | *p*=0.9990 | NC *n*=3, siDDX5 *n*=3 |
| sIL36R:NC vs sIL36R:siDDX5 | *p*=0.9548 | NC *n*=3, siDDX5 *n*=3 |
| sIL36R:NC vs pcmv+IL36γ:NC | *p*<0.0001 | NC *n*=3, siDDX5 *n*=3 |
| sIL36R:NC vs pcmv+IL36γ:siDDX5 | *p*<0.0001 | NC *n*=3, siDDX5 *n*=3 |
| sIL36R:NC vs sIL36R+IL36γ:NC | *p*>0.9999 | NC n=3, siDDX5 n=3 |
| sIL36R:NC vs sIL36R +IL36γ:siDDX5 | *p*=0.7948 | NC *n*=3, siDDX5 *n*=3 |
| sIL36R:siDDX5 vs pcmv+IL36γ:NC | *p*<0.0001 | NC *n*=3, siDDX5 *n*=3 |
| sIL36R:siDDX5 vs pcmv+ IL36γ:siDDX5 | *p*=<0.0001 | NC *n*=3, siDDX5 *n*=3 |
| sIL36R:siDDX5 vs sIL36R+IL36γ:NC | *p*=0.9890 | NC *n*=3, siDDX5 *n*=3 |
| sIL36R:siDDX5 vs sIL36R+ IL36γ:siDDX5 | *p*=0.9998 | NC *n*=3, siDDX5 *n*=3 |
| pcmv+IL36γ:NC vs pcmv+ IL36γ:siDDX5 | *p*=0.7905 | NC *n*=3, siDDX5 *n*=3 |
| pcmv+ IL36γ:NC vs sIL36R + IL36γ:NC | *p*<0.0001 | NC *n*=3, siDDX5 *n*=3 |
| pcmv+IL36γ:NC vs sIL36R+ IL36γ:siDDX5 | *p*=0.0001 | NC *n*=3, siDDX5 *n*=3 |
| pcmv+IL36g:siDDX5 vs sIL36R +IL36γ:NC | *p*<0.0001 | NC *n*=3, siDDX5 *n*=3 |
| pcmv+IL36g:siDDX5 vs sIL36R+ IL36γ:siDDX5 | *p*<0.0001 | NC *n*=3, siDDX5 *n*=3 |
| sIL36R+IL36γ:NC vs sIL36R+ IL36γ:siDDX5 | *p*=0.9013 | NC *n*=3, siDDX5 *n*=3 |

| | | |
|---|---|---|
| *CCL3* | | |
| pcmv:NC vs pcmv:siDDX5 | *p*=0.5376 | NC *n*=3, siDDX5 *n*=3 |
| pcmv:NC vs sIL36R:NC | *p*=0.9605 | NC *n*=3, siDDX5 *n*=3 |
| pcmv:NC vs sIL36R:siDDX5 | *p*=0.7233 | NC *n*=3, siDDX5 *n*=3 |
| pcmv:NC vs sIL36R+IL36γ:NC | *p*<0.0001 | NC *n*=3, siDDX5 *n*=3 |
| pcmv:NC vs pcmv+IL36γ:siDDX5 | *p*=0.0002 | NC *n*=3, siDDX5 *n*=3 |
| pcmv:NC vs sIL36R+IL36γ:NC | *p*=0.2209 | NC *n*=3, siDDX5 *n*=3 |
| pcmv:NC vs sIL36R +IL36γ:siDDX5 | *p*=0.9802 | NC *n*=3, siDDX5 *n*=3 |
| pcmv:siDDX5 vs sIL36R:NC | *p*=0.0163 | NC *n*=3, siDDX5 *n*=3 |
| pcmv:siDDX5 vs sIL36R:siDDX5 | *p*>0.9999 | NC *n*=3, siDDX5 *n*=3 |
| pcmv:siDDX5 vs pcmv+IL36γ:NC | *p*=0.0068 | NC *n*=3, siDDX5 *n*=3 |
| pcmv:siDDX5 vs pcmv+IL-36γ:siDDX5 | *p*<0.0001 | NC *n*=3, siDDX5 *n*=3 |
| pcmv:siDDX5 vs sIL-36R+IL36γ:NC | *p*=0.9971 | NC *n*=3, siDDX5 *n*=3 |
| pcmv:siDDX5 vs IL-36γ:siDDX5 | *p*=0.4576 | NC n=3, siDDX5 n=3 |
| sIL36R:NC vs sIL36R:siDDX5 | *p*=0.9984 | NC *n*=3, siDDX5 *n*=3 |
| sIL36R:NC vs pcmv+IL36γ:NC | *p*=0.0012 | NC *n*=3, siDDX5 *n*=3 |
| sIL36R:NC vs pcmv+IL36γ:siDDX5 | *p*=0.9719 | NC *n*=3, siDDX5 *n*=3 |
| sIL36R:NC vs sIL36R+IL36γ:NC | *p*= <0.0001 | NC *n*=3, siDDX5 *n*=3 |
| sIL36R:NC vs sIL36R +IL36γ:siDDX5 | *p*=0.7596 | NC *n*=3, siDDX5 *n*=3 |
| sIL36R:siDDX5 vs pcmv+IL36γ:NC | *p*=0.1142 | NC *n*=3, siDDX5 *n*=3 |
| sIL36R:siDDX5 vs pcmv+IL36γ:siDDX5 | *p*=0.0037 | NC *n*=3, siDDX5 *n*=3 |
| sIL36R:siDDX5 vs sIL36R+IL36γ:NC | *p*<0.0001 | NC *n*=3, siDDX5 *n*=3 |
| sIL36R:siDDX5 vs sIL36R+IL36γ:siDDX5 | *p*=0.2997 | NC *n*=3, siDDX5 *n*=3 |
| pcmv+IL36γ:NC vs pcmv+IL36γ:siDDX5 | *p*=0.2322 | NC *n*=3, siDDX5 *n*=3 |
| pcmv+ IL36γ:NC vs sIL36R + IL36γ:NC | *p*=0.0244 | NC *n*=3, siDDX5 *n*=3 |
| pcmv+IL36γ:NC vs sIL36R+ IL36γ:siDDX5 | *p*=0.3030 | NC *n*=3, siDDX5 *n*=3 |
| pcmv+IL36γ:siDDX5 vs sIL36R+IL36γ:NC | *p*=0.0002 | NC *n*=3, siDDX5 *n*=3 |
| pcmv+IL36γ:siDDX5 vs sIL36R+ IL36γ:siDDX5 | *p*=0.0026 | NC *n*=3, siDDX5 *n*=3 |
| sIL36R+IL36γ:NC vs sIL36R+ IL36γ:siDDX5 | *p*=0.8283 | NC *n*=3, siDDX5 *n*=3 |
| *CCL11* | | NC *n*=3, siDDX5 *n*=3 |
| pcmv:NC vs pcmv:siDDX5 | *p*= 0.0073 | NC *n*=3, siDDX5 *n*=3 |
| pcmv:NC vs sIL36R:NC | *p*>0.9999 | NC *n*=3, siDDX5 *n*=3 |
| pcmv:NC vs sIL36R:siDDX5 | *p*=0.0838 | NC n=3, siDDX5 n=3 |
| pcmv:NC vs sIL36R+IL36γ:NC | *p*=0.0002 | NC *n*=3, siDDX5 *n*=3 |

| | | |
|---|---|---|
| pcmv:NC vs pcmv+IL36γ:siDDX5 | *p*<0.0001 | NC *n*=3, siDDX5 *n*=3 |
| pcmv:NC vs sIL36R+IL36γ:NC | *p*=0.0094 | NC *n*=3, siDDX5 *n*=3 |
| pcmv:NC vs sIL36R +IL36γ:siDDX5 | *p*<0.0001 | NC *n*=3, siDDX5 *n*=3 |
| pcmv:siDDX5 vs sIL36R:NC | *p*=0.0061 | NC *n*=3, siDDX5 *n*=3 |
| pcmv:siDDX5 vs sIL36R:siDDX5 | *p*=0.8944 | NC n=3, siDDX5 n=3 |
| pcmv:siDDX5 vs pcmv+IL36γ:NC | *p*=0.6356 | NC *n*=3, siDDX5 *n*=3 |
| pcmv:siDDX5 vs pcmv+IL-36γ:siDDX5 | *p*<0.0001 | NC *n*=3, siDDX5 *n*=3 |
| pcmv:siDDX5 vs sIL-36R+IL36γ:NC | *p*>0.9999 | NC *n*=3, siDDX5 *n*=3 |
| pcmv:siDDX5 vs IL-36γ:siDDX5 | *p*<0.0001 | NC *n*=3, siDDX5 *n*=3 |
| sIL36R:NC vs sIL36R:siDDX5 | *p*=0.0708 | NC *n*=3, siDDX5 *n*=3 |
| sIL36R:NC vs pcmv+IL36γ:NC | *p*=0.0002 | NC *n*=3, siDDX5 *n*=3 |
| sIL36R:NC vs pcmv+IL36γ:siDDX5 | *p*<0.0001 | NC *n*=3, siDDX5 *n*=3 |
| sIL36R:NC vs sIL36R+IL36γ:NC | *p*=0.0079 | NC n=3, siDDX5 n=3 |
| sIL36R:NC vs sIL36R +IL36γ:siDDX5 | *p*<0.0001 | NC *n*=3, siDDX5 *n*=3 |
| sIL36R:siDDX5 vs pcmv+IL36γ:NC | *p*=0.1030 | NC *n*=3, siDDX5 *n*=3 |
| sIL36R:siDDX5 vs pcmv+IL36γ:siDDX5 | *p*<0.0001 | NC *n*=3, siDDX5 *n*=3 |
| sIL36R:siDDX5 vs sIL36R+IL36γ:NC | *p*=0.9360 | NC *n*=3, siDDX5 *n*=3 |
| sIL36R:siDDX5 vs sIL36R+IL36γ:siDDX5 | *p*<0.0001 | NC *n*=3, siDDX5 *n*=3 |
| pcmv+IL36γ:NC vs pcmv+IL36γ:siDDX5 | *p*<0.0001 | NC *n*=3, siDDX5 *n*=3 |
| pcmv+ IL36γ:NC vs sIL36R + IL36γ:NC | *p*=0.5576 | NC *n*=3, siDDX5 *n*=3 |
| pcmv+IL36γ:NC vs sIL36R+ IL36γ:siDDX5 | *p*=0.0004 | NC *n*=3, siDDX5 *n*=3 |
| pcmv+IL36γ:siDDX5 vs sIL36R+IL36γ:NC | *p*<0.0001 | NC *n*=3, siDDX5 *n*=3 |
| pcmv+IL36γ:siDDX5 vs sIL36R+ IL36γ:siDDX5 | *p*<0.0001 | NC *n*=3, siDDX5 *n*=3 |
| sIL36R+IL36γ:NC vs sIL36R+ IL36γ:siDDX5 | *p*<0.0001 | NC *n*=3, siDDX5 *n*=3 |
| *CCL17* | | |
| pcmv:NC vs pcmv:siDDX5 | *p*=0.1056 | NC *n*=3, siDDX5 *n*=3 |
| pcmv:NC vs sIL36R:NC | *p*=0.9989 | NC *n*=3, siDDX5 *n*=3 |
| pcmv:NC vs sIL36R:siDDX5 | *p*=0.8081 | NC *n*=3, siDDX5 *n*=3 |
| pcmv:NC vs sIL36R+IL36γ:NC | *p*=0.0023 | NC *n*=3, siDDX5 *n*=3 |
| pcmv:NC vs pcmv+IL36γ:siDDX5 | *p*<0.0001 | NC *n*=3, siDDX5 *n*=3 |
| pcmv:NC vs sIL36R+IL36γ:NC | *p*=0.1205 | NC *n*=3, siDDX5 *n*=3 |
| pcmv:NC vs sIL36R +IL36γ:siDDX5 | *p*=0.3929 | NC *n*=3, siDDX5 *n*=3 |
| pcmv:siDDX5 vs sIL36R:NC | *p*=0.0374 | NC *n*=3, siDDX5 *n*=3 |
| pcmv:siDDX5 vs sIL36R:siDDX5 | *p*=0.7582 | NC *n*=3, siDDX5 *n*=3 |

| | | |
|---|---|---|
| pcmv:siDDX5 vs pcmv+IL36γ:NC | *p*=0.5038 | NC *n*=3, siDDX5 *n*=3 |
| pcmv:siDDX5 vs pcmv+IL-36γ:siDDX5 | *p*<0.0001 | NC *n*=3, siDDX5 *n*=3 |
| pcmv:siDDX5 vs sIL-36R+IL36γ:NC | *p*>0.9999 | NC *n*=3, siDDX5 *n*=3 |
| pcmv:siDDX5 vs IL-36γ:siDDX5 | *p*=0.9878 | NC n=3, siDDX5 n=3 |
| sIL36R:NC vs sIL36R:siDDX5 | *p*=0.4863 | NC *n*=3, siDDX5 *n*=3 |
| sIL36R:NC vs pcmv+IL36γ:NC | *p*=0.0008 | NC *n*=3, siDDX5 *n*=3 |
| sIL36R:NC vs pcmv+IL36γ:siDDX5 | *p*<0.0001 | NC *n*=3, siDDX5 *n*=3 |
| sIL36R:NC vs sIL36R+IL36γ:NC | *p*=0.0431 | NC *n*=3, siDDX5 *n*=3 |
| sIL36R:NC vs sIL36R +IL36γ:siDDX5 | *p*=0.1686 | NC *n*=3, siDDX5 *n*=3 |
| sIL36R:siDDX5 vs pcmv+IL36γ:NC | *p*=0.0396 | NC *n*=3, siDDX5 *n*=3 |
| sIL36R:siDDX5 vs pcmv+IL36γ:siDDX5 | *p*<0.0001 | NC *n*=3, siDDX5 *n*=3 |
| sIL36R:siDDX5 vs sIL36R+IL36γ:NC | *p*=0.7976 | NC *n*=3, siDDX5 *n*=3 |
| sIL36R:siDDX5 vs sIL36R+IL36γ:siDDX5 | *p*=0.9943 | NC *n*=3, siDDX5 *n*=3 |
| pcmv+IL36γ:NC vs pcmv+IL36γ:siDDX5 | *p*<0.0001 | NC *n*=3, siDDX5 *n*=3 |
| pcmv+ IL36γ:NC vs sIL36R + IL36γ:NC | *p*=0.4611 | NC *n*=3, siDDX5 *n*=3 |
| pcmv+IL36γ:NC vs sIL36R+ IL36γ:siDDX5 | *p*=0.1498 | NC *n*=3, siDDX5 *n*=3 |
| pcmv+IL36γ:siDDX5 vs sIL36R+IL36γ:NC | *p*<0.0001 | NC *n*=3, siDDX5 *n*=3 |
| pcmv+IL36γ:siDDX5 vs sIL36R+ IL36γ:siDDX5 | *p*<0.0001 | NC *n*=3, siDDX5 *n*=3 |
| sIL36R+IL36γ:NC vs sIL36R+ IL36γ:siDDX5 | *p*=0.9930 | NC *n*=3, siDDX5 *n*=3 |
| *CCL27* | | NC *n*=3, siDDX5 *n*=3 |
| pcmv:NC vs pcmv:siDDX5 | *p*=0.9626 | NC *n*=3, siDDX5 *n*=3 |
| pcmv:NC vs sIL36R:NC | *p*>0.9999 | NC *n*=3, siDDX5 *n*=3 |
| pcmv:NC vs sIL36R:siDDX5 | *p*=0.9841 | NC n=3, siDDX5 n=3 |
| pcmv:NC vs sIL36R+IL36γ:NC | *p*=0.6596 | NC *n*=3, siDDX5 *n*=3 |
| pcmv:NC vs pcmv+IL36γ:siDDX5 | *p*=0.0064 | NC *n*=3, siDDX5 *n*=3 |
| pcmv:NC vs sIL36R+IL36γ:NC | *p*=0.9208 | NC *n*=3, siDDX5 *n*=3 |
| pcmv:NC vs sIL36R +IL36γ:siDDX5 | *p*=0.0049 | NC *n*=3, siDDX5 *n*=3 |
| pcmv:siDDX5 vs sIL36R:NC | *p*=0.9720 | NC *n*=3, siDDX5 *n*=3 |
| pcmv:siDDX5 vs sIL36R:siDDX5 | *p*>0.9999 | NC n=3, siDDX5 n=3 |
| pcmv:siDDX5 vs pcmv+IL36γ:NC | *p*=0.9950 | NC *n*=3, siDDX5 *n*=3 |
| pcmv:siDDX5 vs pcmv+IL-36γ:siDDX5 | *p*=0.0468 | NC *n*=3, siDDX5 *n*=3 |
| pcmv:siDDX5 vs sIL-36R+IL36γ:NC | *p*>0.9999 | NC *n*=3, siDDX5 *n*=3 |
| pcmv:siDDX5 vs IL-36γ:siDDX5 | *p*=0.0363 | NC *n*=3, siDDX5 *n*=3 |

| | | |
|---|---|---|
| sIL36R:NC vs sIL36R:siDDX5 | *p*=0.9890 | NC *n*=3, siDDX5 *n*=3 |
| sIL36R:NC vs pcmv+IL36γ:NC | *p*=0.6916 | NC *n*=3, siDDX5 *n*=3 |
| sIL36R:NC vs pcmv+IL36γ:siDDX5 | *p*=0.0071 | NC *n*=3, siDDX5 *n*=3 |
| sIL36R:NC vs sIL36R+IL36γ:NC | *p*=0.9366 | NC n=3, siDDX5 n=3 |
| sIL36R:NC vs sIL36R +IL36γ:siDDX5 | *p*=0.0055 | NC *n*=3, siDDX5 *n*=3 |
| sIL36R:siDDX5 vs pcmv+IL36γ:NC | *p*=0.9849 | NC *n*=3, siDDX5 *n*=3 |
| sIL36R:siDDX5 vs pcmv+IL36γ:siDDX5 | *p*=0.0352 | NC *n*=3, siDDX5 *n*=3 |
| sIL36R:siDDX5 vs sIL36R+IL36γ:NC | *p*>0.9999 | NC *n*=3, siDDX5 *n*=3 |
| sIL36R:siDDX5 vs IL36R+ IL36γ:siDDX5 | *p*=0.0272 | NC *n*=3, siDDX5 *n*=3 |
| pcmv+IL36γ:NC vs pcmv+ IL36γ:siDDX5 | *p*=0.1687 | NC *n*=3, siDDX5 *n*=3 |
| pcmv+ IL36γ:NC vs sIL36R + IL36γ:NC | *p*=0.9991 | NC *n*=3, siDDX5 *n*=3 |
| pcmv+IL36γ:NC vs sIL36R+ IL36γ:siDDX5 | *p*=0.1345 | NC *n*=3, siDDX5 *n*=3 |
| pcmv+IL36γ:siDDX5 vs sIL36R +IL36γ:NC | *p*=0.0642 | NC *n*=3, siDDX5 *n*=3 |
| pcmv+IL36γ:siDDX5 vs sIL36R+ IL36γ:siDDX5 | *p*>0.9999 | NC *n*=3, siDDX5 *n*=3 |
| sIL36R+IL36γ:NC vs sIL36R+ IL36γ:siDDX5 | *p*=0.0500 | NC *n*=3, siDDX5 *n*=3 |
| **Extended Data Fig. 9d** | | |
| *Ccl20* | *p*=0.0060 | PBS *n*=5, sIL-36R *n*=5 |
| *Cxcl1* | *p*=0.0032 | PBS *n*=5, sIL-36R *n*=5 |
| *Il23* | *p*=0.0022 | PBS *n*=5, sIL-36R *n*=5 |
| *Il17a* | *p*=0.0002 | PBS *n*=5, sIL-36R *n*=5 |
| **Extended Data Fig. 9e** | | |
| PBS *vs* sIL-36R | *p*=0.0043 | PBS *n*=6, sIL-36R *n*=6 |
| **Extended Data Fig. 9f** | | |
| $CD45^+$ cells in skin (%) | *p*=0.0010 | PBS *n*=6, sIL-36R *n*=6 |
| Neutrophils in skin (%) | *p*=0.0158 | PBS *n*=6, sIL-36R *n*=6 |
| $MHCII^+$ antigen-presenting cell in skin (%) | *p*=0.0178 | PBS *n*=6, sIL-36R *n*=6 |
| $CD3^+\gamma\delta T^+$ cells in skin (%) | *p*=0.0122 | PBS *n*=6, sIL-36R *n*=6 |
