# Supplementary material for: IL-17D-induced inhibition of DDX5 expression in keratinocytes amplifies IL-36R-mediated skin inflammation
Source: Nat Immunol. 2022 Oct 21;23(11):1577–87. doi: 10.1038/s41590-022-01339-3 (PMC9663298; doi:10.1038/s41590-022-01339-3)
Supplement: Supplementary file 2 — Reporting Summary [file 41590_2022_1339_MOESM2_ESM.pdf]

nature portfolio

Corresponding author(s): Yuping Lai

Last updated by author(s): Sep 10, 2022

Reporting Summary

Nature Portfolio wishes to improve the reproducibility of the work that we publish. This form provides structure for consistency and transparency in reporting. For further information on Nature Portfolio policies, see our Editorial Policies and the Editorial Policy Checklist.

Statistics

For all statistical analyses, confirm that the following items are present in the figure legend, table legend, main text, or Methods section.

n/a	Confirmed	
☐	☒	The exact sample size (*n*) for each experimental group/condition, given as a discrete number and unit of measurement
☐	☒	A statement on whether measurements were taken from distinct samples or whether the same sample was measured repeatedly
☐	☒	The statistical test(s) used AND whether they are one- or two-sided<br>*Only common tests should be described solely by name; describe more complex techniques in the Methods section.*
☐	☒	A description of all covariates tested
☐	☒	A description of any assumptions or corrections, such as tests of normality and adjustment for multiple comparisons
☐	☒	A full description of the statistical parameters including central tendency (e.g. means) or other basic estimates (e.g. regression coefficient) AND variation (e.g. standard deviation) or associated estimates of uncertainty (e.g. confidence intervals)
☐	☒	For null hypothesis testing, the test statistic (e.g. *F*, *t*, *r*) with confidence intervals, effect sizes, degrees of freedom and *P* value noted<br>*Give P values as exact values whenever suitable.*
☒	☐	For Bayesian analysis, information on the choice of priors and Markov chain Monte Carlo settings
☒	☐	For hierarchical and complex designs, identification of the appropriate level for tests and full reporting of outcomes
☒	☐	Estimates of effect sizes (e.g. Cohen's *d*, Pearson's *r*), indicating how they were calculated

Our web collection on statistics for biologists contains articles on many of the points above.

Software and code

Policy information about availability of computer code

Data collection

qRT-PCR was performed on a StepOnePlus™ Real-Time PCR System (Applied Biosystem).
RNA sequencing was performed on Hiseq (Illumina).
The processed RNA-Seq datasets for atopic dermatitis and psoriasis were downloaded from GEO database (GSE121212).
The processed scRNA-seq data was downloaded from doi: 10.5281/zenodo.4310074.
ELISA data were recorded with SPECTROstar Nano.
Flow cytometry data were collected on Fortessa (BD Biosciences).
The mass spectrometry resulting peptides were analyzed on the HPLC liquid system Dionex Ultimate 3000 (Thermo Scientific) coupled to a Dionex Trap column (100μm X 2cm X 5μm) with in-house packed C18 column (75μm X 15cm X 3μm) .

Data analysis

GraphPad Prism v9.0 was used for analyzing qRT-PCR, ELISA data and all the statistical analyses.
FastQC (version 0.11.9) was used for assessing the overall quality of raw reads and Trimmomatic (version 0.39) was applied for raw reads quality control to cut adapters and remove low-quality reads. And then all the clean reads were mapped onto human hg38 genome using Hisat2 (version 2.1.0). Samtools (version: 1.7) was used to convert SAM format files to BAM format files and sorted the BAM files. Gene expression levels were quantified by FeatureCounts (version 1.6.3). Differential expression analysis was performed by R package DESeq2 (Version 1.34.0). Gene ontology (GO) and KEGG pathway enrichment analyses were performed in ClusterProfiler (version 4.2.0). Differential alternative splicing events were detected by rMATS (version 3.1.0).
The processed scRNA-seq data was converted to Seurat object using SeuratDisk (https://mojaveazure.github.io/seurat-disk/).Followed by the typical Seurat workflow (http://satijalab.org/seurat/), 2000 highly variable genes were normalized, scaled and identified by using the NormalizeData, ScaleData and FindVariableGenes function from Seurat. The number of positive cells was determined by visual inspection of the ElbowPlot. Uniform Manifold Approximation and Projection (UMAP) with a resolution of 0.5 was used to determine cell clusters. The

annotation information was reserved to define clusters. Cell subsets were combined and the expression level was visualized by VlnPlot from Seurat.
Flow cytometry data were analyzed by FLowJo v10.0 software (TreeStar) .
The mass spectrometry resulting peptides was analyzed by maXis HDTM-UHR-TOF mass spectrometer (Bruker). The spectra from mass spectrometry were automatically used for searching against the nonredundant International Protein Index human protein database (version 3.72) with the Bioworks browser (rev.3.1).
ESEfinder program (http://exon.cshl.edu/ESE) was used for evaluated the potential binding ESEs.

For manuscripts utilizing custom algorithms or software that are central to the research but not yet described in published literature, software must be made available to editors and reviewers. We strongly encourage code deposition in a community repository (e.g. GitHub). See the Nature Portfolio guidelines for submitting code & software for further information.

Data

Policy information about availability of data

All manuscripts must include a data availability statement. This statement should provide the following information, where applicable:

- Accession codes, unique identifiers, or web links for publicly available datasets
- A description of any restrictions on data availability
- For clinical datasets or third party data, please ensure that the statement adheres to our policy

The raw sequence data reported in this paper have been deposited in Gene Expression Omnibus (GEO) Database under accession numbers GSE208666, GSE208669 and GSE208671. The mass spectrometry proteomics data have been deposited to the ProteomeXchange Consortium (http://www.proteomexchange.org) under the accession number PXD021379. All other data supporting the findings of this study are available within the paper or from the corresponding author upon request.

Field-specific reporting

Please select the one below that is the best fit for your research. If you are not sure, read the appropriate sections before making your selection.

☒ Life sciences ☐ Behavioural & social sciences ☐ Ecological, evolutionary & environmental sciences

For a reference copy of the document with all sections, see nature.com/documents/nr-reporting-summary-flat.pdf

Life sciences study design

All studies must disclose on these points even when the disclosure is negative.

Sample size	For all animal studies, we performed preliminary experiments to determine requirements for sample size, and at least twice independent experiments were performed to ensure reproducibility. For all in vitro experiments, at least 3 independent biological replicates were used and three independent experiments were performed , with few exceptions in which experiments were repeated twice.
Data exclusions	In ELISA,the samples with low yield of protein were pre-determined and excluded.
Replication	All in vivo experiments were repeated at least twice, and in vitro experiments were conducted with three times with few exceptions in which experiments were repeated twice. Each replicated assay obtained the similar results. In each individual experiment, each technical replicate was measured once.
Randomization	Mice were grouped according to genotype and littermates were used for each animal experiment.
Blinding	All in vivo experiments were not performed in a blinded manner, because mice were grouped based on and compared across different genotypes, and different genotypes have distinct disease manifestation. However,we kept all experiments as unbiased as possible. Each psoriatic and AD model and measurement were performed by the same researcher to ensure reproducibility. Proper internal controls and normalization methods were included in each study forinternal bias.

Reporting for specific materials, systems and methods

We require information from authors about some types of materials, experimental systems and methods used in many studies. Here, indicate whether each material, system or method listed is relevant to your study. If you are not sure if a list item applies to your research, read the appropriate section before selecting a response.

Materials & experimental systems

n/a	Involved in the study
☐	☒ Antibodies
☐	☒ Eukaryotic cell lines
☒	☐ Palaeontology and archaeology
☐	☒ Animals and other organisms
☐	☒ Human research participants
☒	☐ Clinical data
☒	☐ Dual use research of concern

Methods

n/a	Involved in the study
☒	☐ ChIP-seq
☐	☒ Flow cytometry
☒	☐ MRI-based neuroimaging

Antibodies

Antibodies used

Antibodies for Western blot, Immunofluorescent staining and immunoprecipitation:
Anti-DDDDK-tag: MBL, Cat #M185-3L, Clone # FLA-1, Lot#006,1:10000
Anti-HA-tag: MBL, Cat #M180-3S, Clone #TANA2, Lot#007,1:10000
Anti-His-tag: MBL, Cat #D291-3S, Clone #OGHIS, Lot#008,1:5000
Anti-β-Actin: Sigma, Cat #A5541, Clone #AC-15, Lot# 122M4782,1:5000
Anti-GAPDH: Proteintech, Cat #60004-1-Ig, Clone #Ag0766, Lot# 10013030 ,1:5000
Anti-DDX5: Abcam, Cat #ab126730, Clone #EPR7239, Lot# GR3273719-4,1:1000 for Immunoblotting,1:100 for Immunofluorescence
Anti-IL-17D: Thermo Fisher, Cat#MA5-24033, Clone# 312724, Lot#312724,1:500
Anti-SF2: Abcam, Cat #ab133689, Clone #EPR8240, Lot# GR97894-10,1:1000
Phospho-p38 MAPK (Thr180/ Tyr182) Antibody: CST, Cat #9211, Clone #D3F9, Lot#0022,1:1000
p38 MAPK Antibody: CST, Cat # 9212S, Clone #D3F9, Lot# 0023,1:1000
Phospho-AKT(Ser473): CST, Cat#4060, Clone #DE9, Lot#0012,1:1000
AKT(Pan) antibody: CST, Cat#4691, Clone #C67E7, Lot#0011,1:1000
Phospho-SMAD2(Ser465/467)/SMAD3(Ser423/425): CST, Cat#8828S, Clone #D27F4, Lot#008,1:1000
SMAD2/3 Antibody：CST, Cat#8685S, Clone #D7G7, Lot#007,1:1000
Phospho-NF-κB p65 (Ser536) (93H1) Rabbit Mab: CST, Cat#3033S, Clnoe#93H1, Lot#0017,1:1000
NF-κB p65 Rabbit mAB: CST, Cat #4764S, Clnoe#C22B4, Lot#0016,1:1000
Phospho-SAPK/JNK (Thr183/Tyr185)(81E11) Rabbit mAB: CST, Cat#4668S, Clone#81E11, Lot#0022,1:1000
Recombinant Anti-JNK2 Antibody, Rabbit monoclonal: Sino Biological, Cat#10745-R004, Clone#011, Lot#HB06SE1405,1:1000
Human IL-36 gamma/IL-1F9 Antibody: R&D, Cat#AF2320, Clone #Q9NZH8, Lot# UNN0112091,1:500
IL36γ Rabbit pAb: ABclonal, Cat # A10165, Clone #Q82460, Lot#Q2062,1:1000
anti-IL-36R antibody N-terminal mAb: Abcam,Cat#ab210933,Clone#ABM47A2, Lot#GR318274-1,1:1000
Mouse IL-1Rrp2/IL-1R6 Antibody: R&D, Cat#AF2354-SP, Clone# Q9ERS7, Lot# WVV0217081,1:1000
anti-Rabbit IgG: Abmart, Cat #B30011M, Lot#294670,1:20000
anti-Mouse IgG: Abmart, Cat #B30010M, Lot#294656,1:20000
Goat anti-Rabbit IgG (H+L) Cross-Adsorbed Secondary Antibody Alexa Fluor 488: Invitrogen, Cat #A-11008, Lot#1672238,1:1000
Goat anti-Mouse IgG (H+L) Cross-Adsorbed Secondary Antibody Alexa Fluor 488: Invitrogen, Cat#A-11011, Lot#2318440,1:1000
AffiniPure Donkey Anti-Mouse IgG (H+L): Jackson ImmunoResearch, Cat #715-005-151, Clone#AB_2340759, Lot#147635,1:10000
AffiniPure Goat Anti-Rabbit IgG (H+L): Jackson ImmunoResearch, Cat #111-005-003, Clone# AB_2337913, Lot#152677,1:10000
AffiniPure Donkey Anti-Goat IgG (H+L): Jackson ImmunoResearch, Cat#705-065-147, Clone#AB_2340385, Lot#119956,1:10000
Antibodies for FACS:
PE Anti-His: BioLegend, Cat #362603, Clone#J095G46, Lot#B226473,0.25μg/10^6 cell
Zombie Violet Dye: BioLegend, Cat #423113, Lot#B281932,1:1000
PE Anti-Mouse CD45: BioLegend, Cat #103105, Clone#30-F11, Lot#B294742,0.25μg/10^6 cell
APC Anti-Mouse CD45: BioLegend, Cat #103111, Clone#30-F11, Lot#B308253,0.25μg/10^6 cell
FITC Anti-Mouse CD45: BioLegend, Cat #103108, Clone#30-F11, Lot#B246762,0.25μg/10^6 cell
FITC Anti-Mouse CD3: BioLegend, Cat #100203, Clone#17A2, Lot#B313076,0.25μg/10^6 cell
APC Anti-Mouse γδTCR: BioLegend, Cat #118116, Clone#GL3, Lot#B228498,0.25μg/10^6 cell
PE Anti-Mouse CD11b: BioLegend, Cat #101207, Clone#M1/70, Lot#B253921,0.25μg/10^6 cell
FITC Anti-Mouse CD11b: BioLegend, Cat #101205, Clone#M1/70, Lot#B324795,0.25μg/10^6 cell
FITC Anti-Mouse Ly6G: BioLegend, Cat #108405, Clone#RB6-8C5, Lot#B281018,0.25μg/10^6 cell
APC Anti-Mouse CD11c: BioLegend, Cat #117309, Clone#N418, Lot#B297573,0.25μg/10^6 cell
PE Anti-Mouse CD11c: BioLegend, Cat #117308, Clone#N418, Lot#B234524,0.25μg/10^6 cell
PerCP/Cy5.5 Anti-Mouse IA-IE: BioLegend, Cat #107625, Clone#M5/114.15.2, Lot#B261237,0.25μg/10^6 cell
PE Anti-Mouse CD170: BioLegend, Cat #155505, Clone#S17007L, Lot#B301118,0.25μg/10^6 cell
PerCP/Cyanine5.5 Anti-Mouse CD49b: BioLegend, Cat #103519, Clone#HMα2, Lot#B294947,0.25μg/10^6 cell
FITC Anti-Mouse IgE: BioLegend, Cat #406905, Clone#RME-1, Lot#B316961,0.25μg/10^6 cell
PerCP/Cyanine5.5 Anti-Mouse CD4: BioLegend, Cat #100433, Clone#GK1.5, Lot#B248433,0.25μg/10^6 cell
Antibodies for ELISA:
Mouse Il13 ELISA Ready-SET-Go: eBioscience, Cat#88-7137-88, Lot#E09414
Mouse Tslp ELISA Kit: MULTI SCIENCES, Cat#EK265/2-96, Lot#A26590752
Mouse Il4 ELISA Kit: MULTI SCIENCES, Cat#EK204/2-96, Lot#A20400253
DuoSet mouse Ccl20: R&D, Cat#DY760, Lot#P167295
DuoSet mouse Cxcl1: R&D, Cat#DY453-05, Lot#P179973
DuoSet mouse Il23: R&D, Cat#DY1887-05, Lot#P190436
DuoSet mouse Il17a: R&D, Cat#DY421-05, Lot#P328895
Mouse Tnfα: BD Pharmingen, Cat#51-9004717, Lot#10211
Mouse I17f: BD Pharmingen, Cat#562174, Lot#8162567

	Other antibodies:<br>Anti-Mouse sIL36R &Anti-Human sIL36R made by Sirtomics Biotechnology Company who was entrusted by our laboratory.
Validation	All above commercial antibodies are well validated by the manufacturer. Specificity and validation were provided by manufacturer's technical datasheets and confirmed in literature.Please refer to the spec sheets on the respective vendors' websites for technical information and detail by searching with the catalog numbers provided. For Anti-Mouse sIL36R &Anti-Human sIL36R antibody, we did western blotting to test their specificity.

Eukaryotic cell lines

Policy information about cell lines

Cell line source(s)	HEK293T cells and HeLa cells were provided by Prof. Wong from East China Normal University who purchased these cell lines from ATCC, HaCaT cells were purchased from Cobioer.
Authentication	HEK293T cell line and HeLa cells were not tested and authenticated by our laboratory. HaCaT cell line has been tested and authenticated, using morphology, karyotyping and PCR based approaches to confirm the identity by the vendor.
Mycoplasma contamination	All cell lines were tested for mycoplasma contamination and were confirmed negative.
Commonly misidentified lines<br>(See ICLAC register)	No commonly misidentified cell lines were used.

Animals and other organisms

Policy information about studies involving animals; ARRIVE guidelines recommended for reporting animal research

Laboratory animals	Mice with BABL/c or C57BL/6 background were bred in the specific-pathogen-free animal facility at East China Normal University.Il17d–/– and CD93–/– mice were bred in the specific-pathogen-free animal facility at Tsinghua University. DDX5fl/fl and sIL36R fl/fl mice were generated by Shanghai Model Organisms Center, Inc. The K14Cre transgenic mice were obtained from Shanghai Model Organisms Center, Inc, while the K5Cre transgenic mice were obtained from Xiao Yang lab in the Academy of Military Medical Sciences in China. DDX5fl/fl mice were crossed with the K14Cre transgenic mice to generate Ddx5ΔKC mice, sIL36Rfl/fl mice were crossed with the K5Cre mice to generate sIL36RTg/KC mice, and Ddx5ΔKC mice were crossed with sIL36RTg/KC mice to generate Ddx5ΔKCsIL36RTg/KC mice. Both male and female mice aged at 7-8 weeks old were used in all experiments.
Wild animals	No wild animals were used in this study.
Field-collected samples	No field-collected samples were used in this study.
Ethics oversight	All the animal experiments were performed with the use of the protocols (Protocol No.: m20210233 for AD, m20200316 for psoriasis) approved by the Animal Care and Use Committee at East China Normal University.

Note that full information on the approval of the study protocol must also be provided in the manuscript.

Human research participants

Policy information about studies involving human research participants

Population characteristics	Patients with atopic dermatitis: moderate to severe, 3 men and 4 women; patients with psoriasis: mild plaque-type psoriasis, 4 men and 6 women; patients with basal cell carcinoma:1 man and 2women; patients with squamous cell carcinoma:2 men and 1woman; normal patients: 4 men and 4 women. Patients' age are from 14-80 years old.
Recruitment	Before enrolling in the study, all patients were volunteers and aware of the subjects of the study and signed the informed consent. All patients are from Shanghai, China. The only criteria for inclusion were healthy, AD, psoriasis, SCC or BCC.
Ethics oversight	The Ethics Review Committees of Huashan Hospital or Shanghai Tenth People's Hospital approved the protocols (KY2020732 for AD, SHSY-IEC-KY-4.0/18-13/01 for psoriasis)used in this study.

Note that full information on the approval of the study protocol must also be provided in the manuscript.

Flow Cytometry

Plots

Confirm that:

- [x] The axis labels state the marker and fluorochrome used (e.g. CD4-FITC).
- [x] The axis scales are clearly visible. Include numbers along axes only for bottom left plot of group (a 'group' is an analysis of identical markers).
- [x] All plots are contour plots with outliers or pseudocolor plots.
- [x] A numerical value for number of cells or percentage (with statistics) is provided.

Methodology

Sample preparation

For the skin samples, the epidermis and dermis were separated using dispase II (Sigma; 5mg/mL in HBSS; 37°C for 90 min). The dermal cells were separated by collagenase (Roche) and hyaluronidase (Sigma) digestion [10mM Hepes, collagenase D (2.5mg/mL), hyaluronidase (100U/mL), and deoxyribonuclease (50μg/mL) in DMEM (GIBICO); 37°C for 45 min]. Isolated cells were stained with different cell surface markers [CD45 for leukocytes; CD45+, CD49b+,IgE+ for basophils; CD45+, CD11b+, SiglecF+ for eosinophils; CD45+, CD3+, CD4+ for CD4+ T cells; CD45+, CD11b+, Ly6G+ for neutrophils; CD45+, CD11c+ or MHCII+ for antigen presenting cells; CD45+, CD3+, γδT cell receptor (TCR+) for γδT cells]. The cells were then fixed and the relevant isotype control mAbs were used.

Instrument

BD LSR Fortessa

Software

FlowJo v10

Cell population abundance

All frequencies of cells are stated in the representative graphs and quantifications.

Gating strategy

Cells were first gated based on FSC-A and SSC-A to exclude debris, and then gated by live-dead dye to exclude dead cells. Specific cell populations were determined by markers listed as follows: CD45 for leukocytes; CD45+ CD11b+Ly6G+ for neutrophils; CD45+CD11c+ or MHCII+ for dendritic cells; CD45+CD3+γδT cell receptor (TCR+) for γδT cells; CD45+ ,CD49b+, IgE+ for basophils; CD45+CD11b+ SiglecF+ for eosinophils; CD45+ CD3+CD4+ for CD4+ T cells.

☒ Tick this box to confirm that a figure exemplifying the gating strategy is provided in the Supplementary Information.
