# Supplementary figures and images for: IL-17D-induced inhibition of DDX5 expression in keratinocytes amplifies IL-36R-mediated skin inflammation
Source: Nat Immunol. 2022 Oct 21;23(11):1577–87. doi: 10.1038/s41590-022-01339-3 (PMC9663298; doi:10.1038/s41590-022-01339-3)

Source Data Figure 1 – Unprocessed Immunoblots

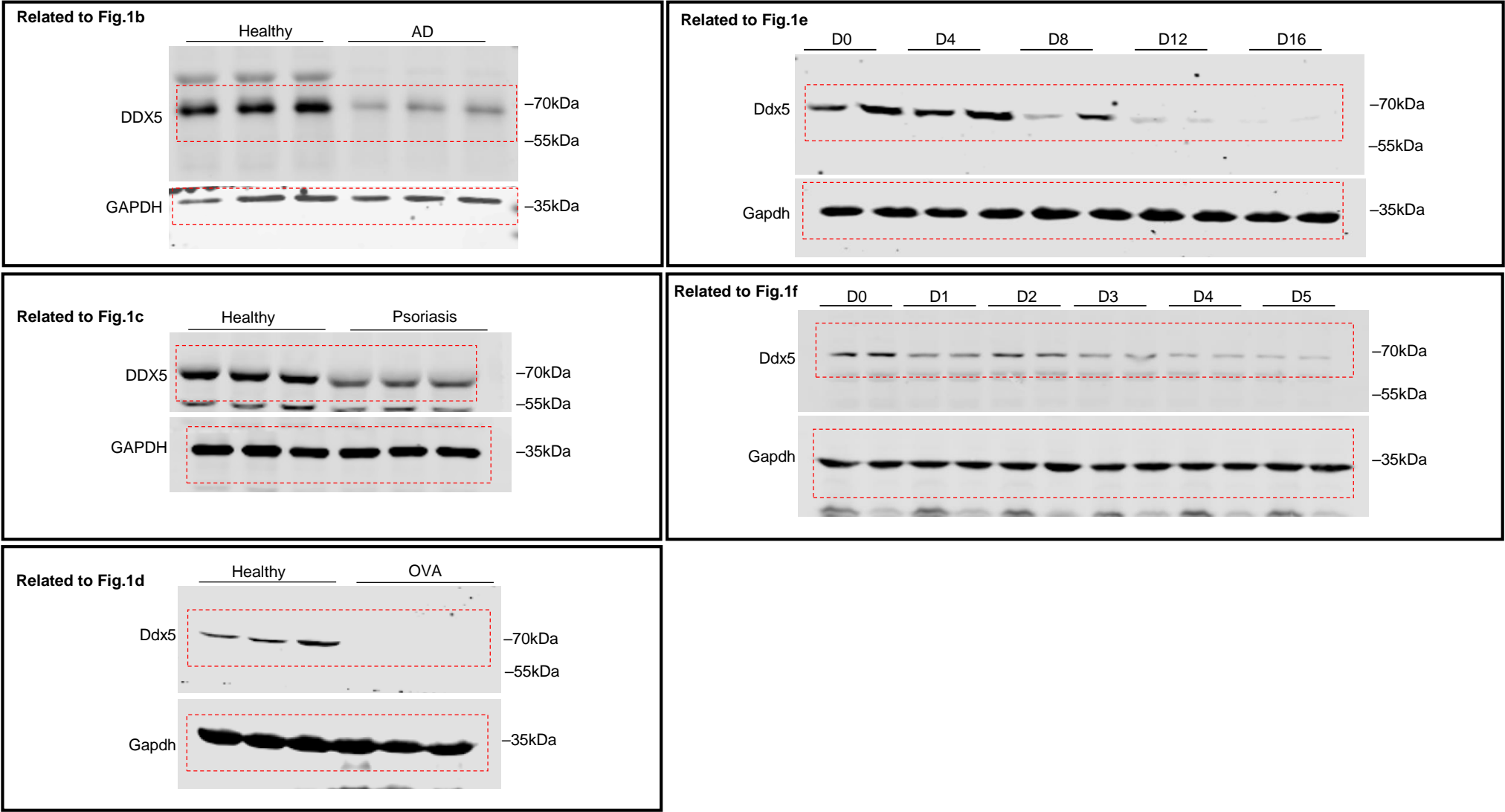

Supplement: Source Data Fig. 1 — Unprocessed immunoblots. [file 41590_2022_1339_MOESM5_ESM.pdf]

Source Data Figure 2 – Unprocessed Immunoblots

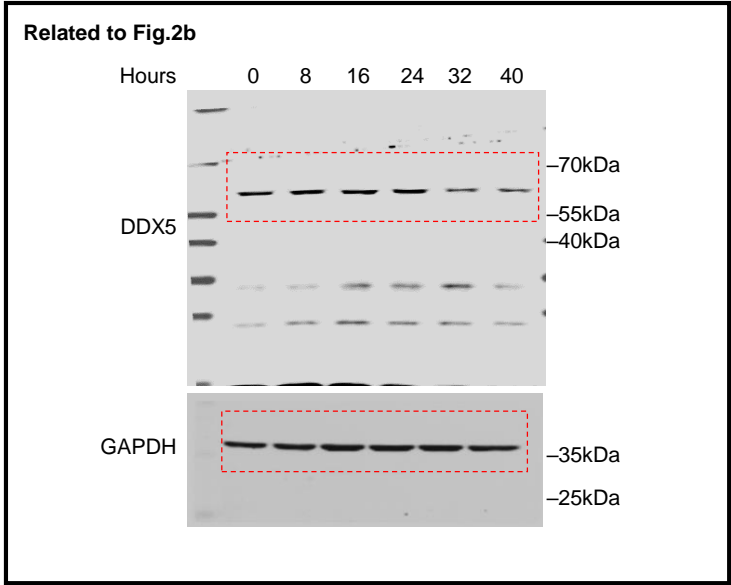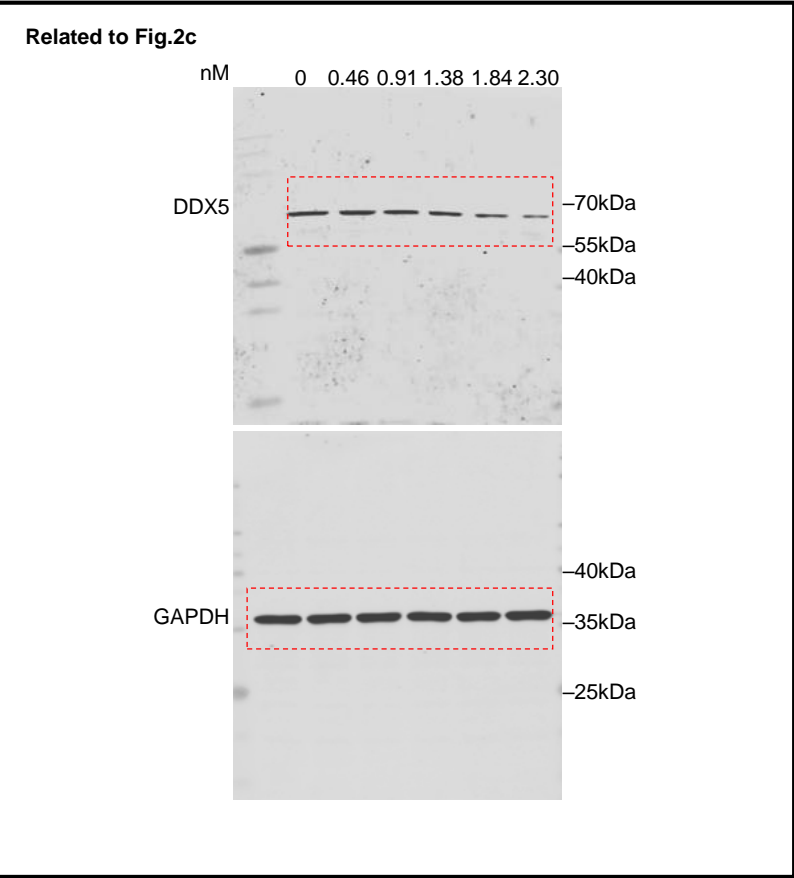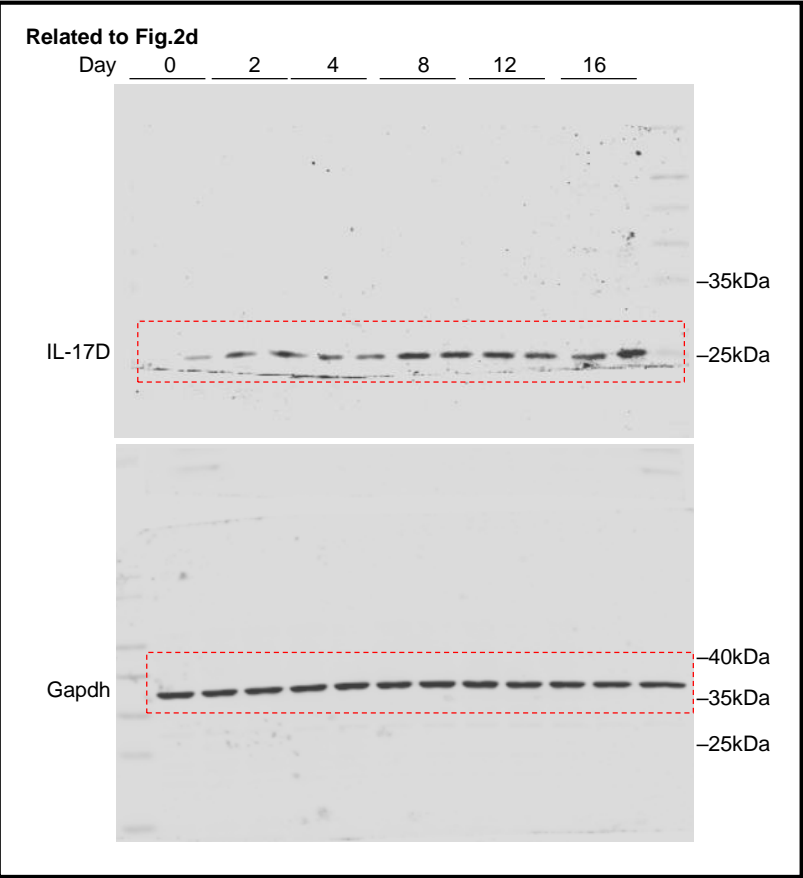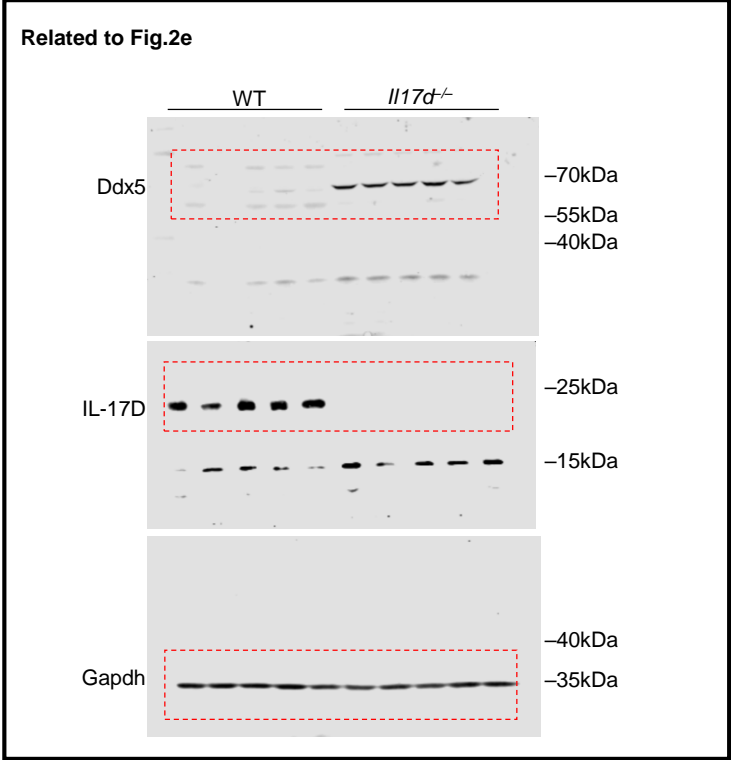

Supplement: Source Data Fig. 2 — Unprocessed immunoblots. [file 41590_2022_1339_MOESM7_ESM.pdf]

Source Data Figure 3 – Unprocessed Immunoblots

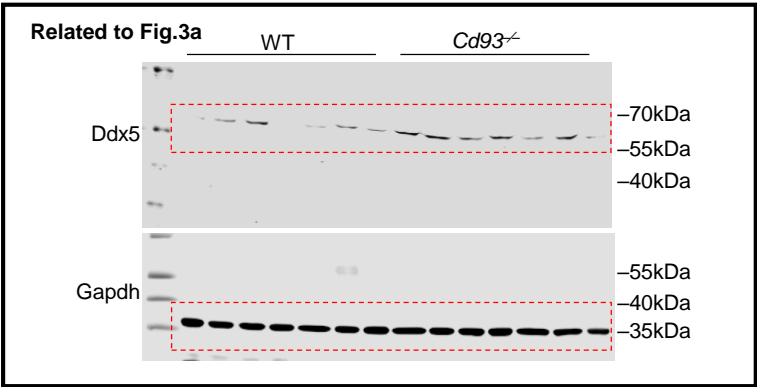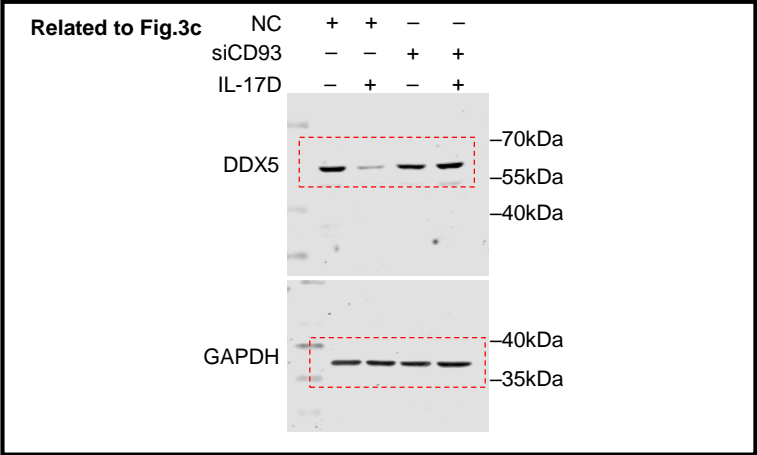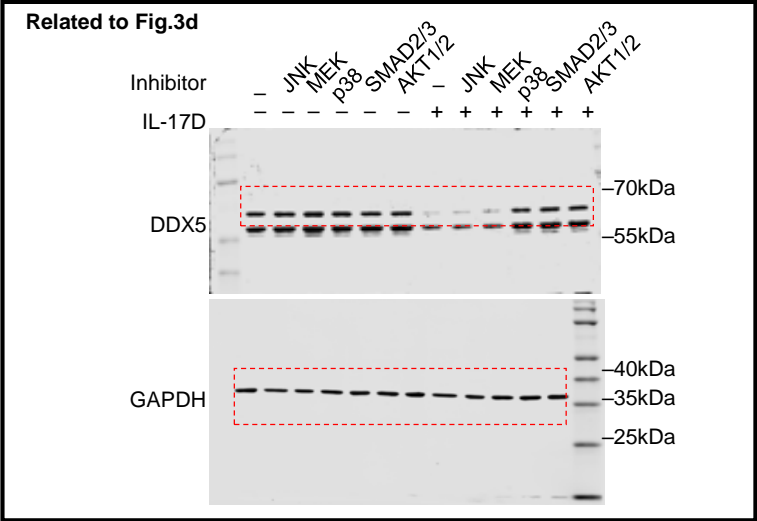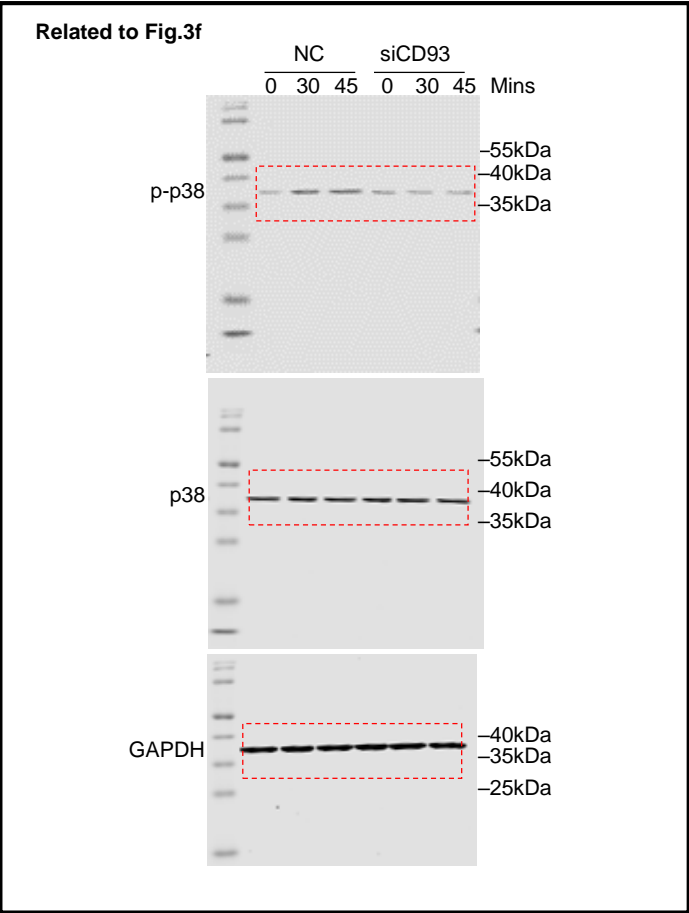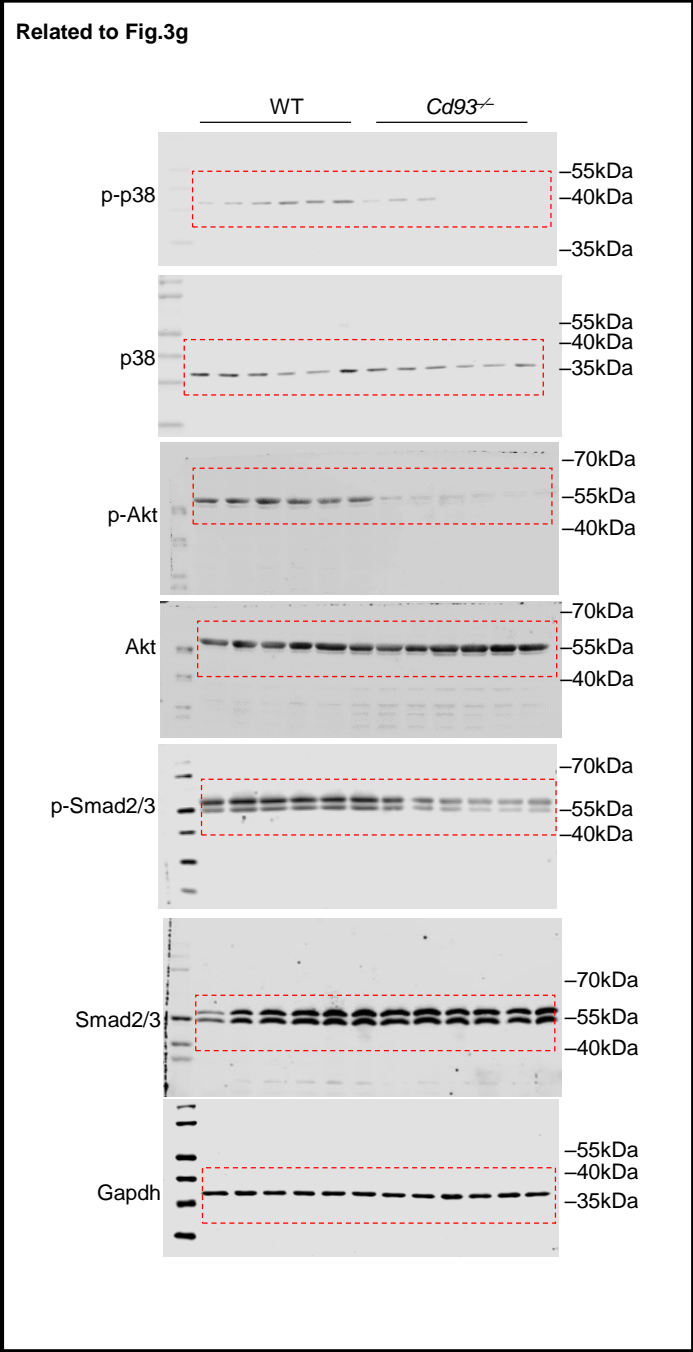

Source Data Figure 3 – Unprocessed Immunoblots

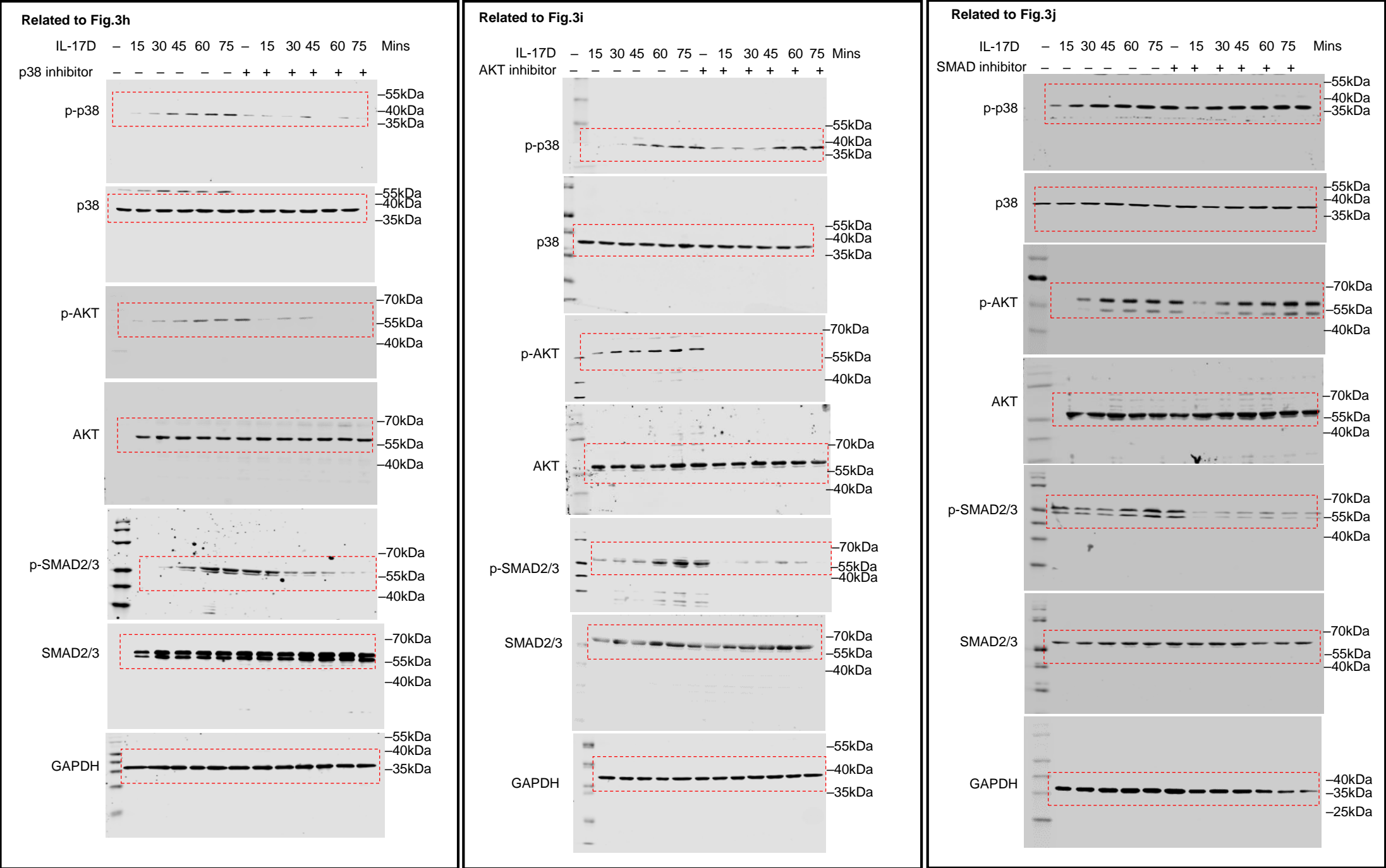

Supplement: Source Data Fig. 3 — Unprocessed immunoblots. [file 41590_2022_1339_MOESM9_ESM.pdf]

Source Data Figure 5 – Unprocessed Immunoblots

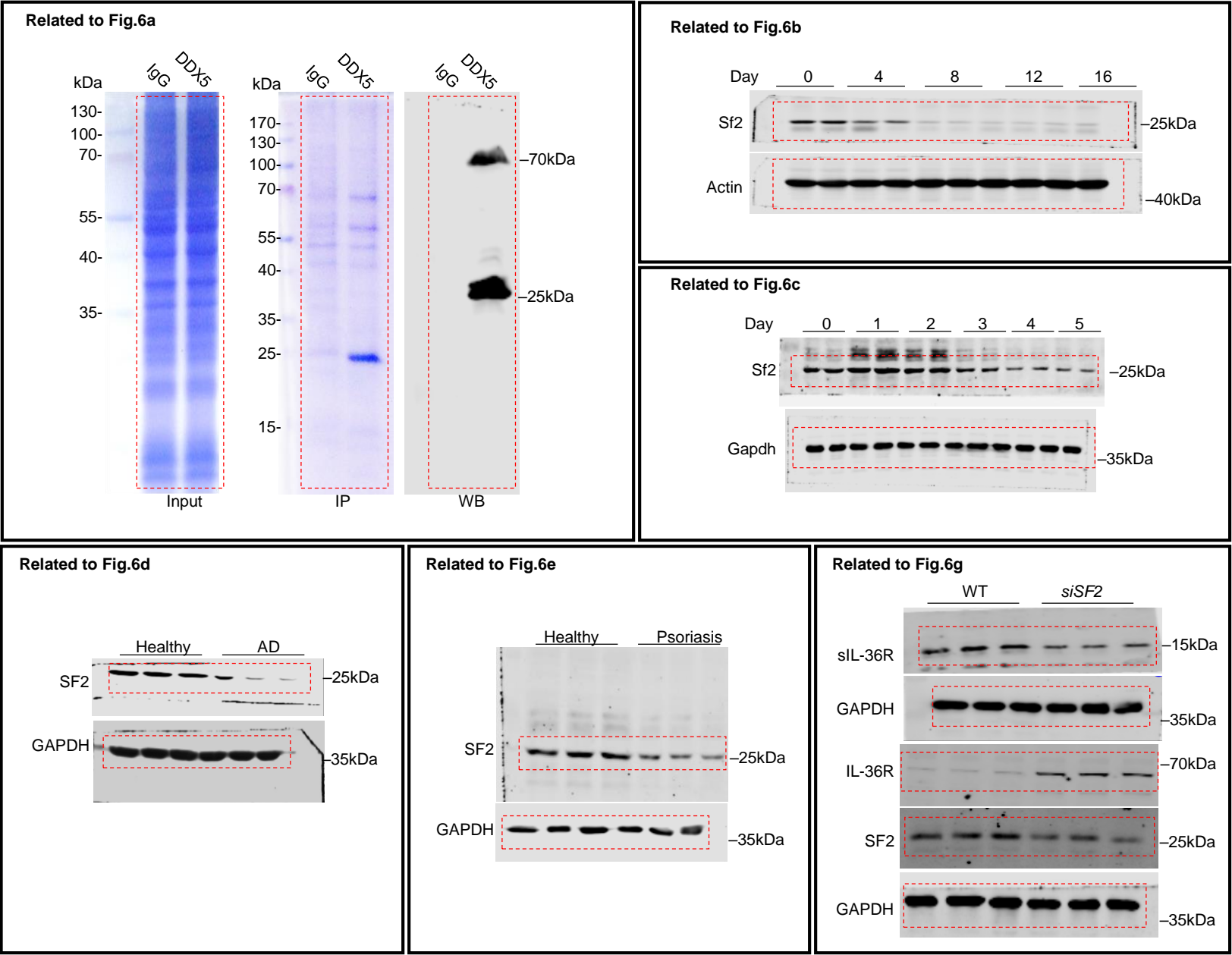

### Source Data Figure 6 – Unprocessed Gels

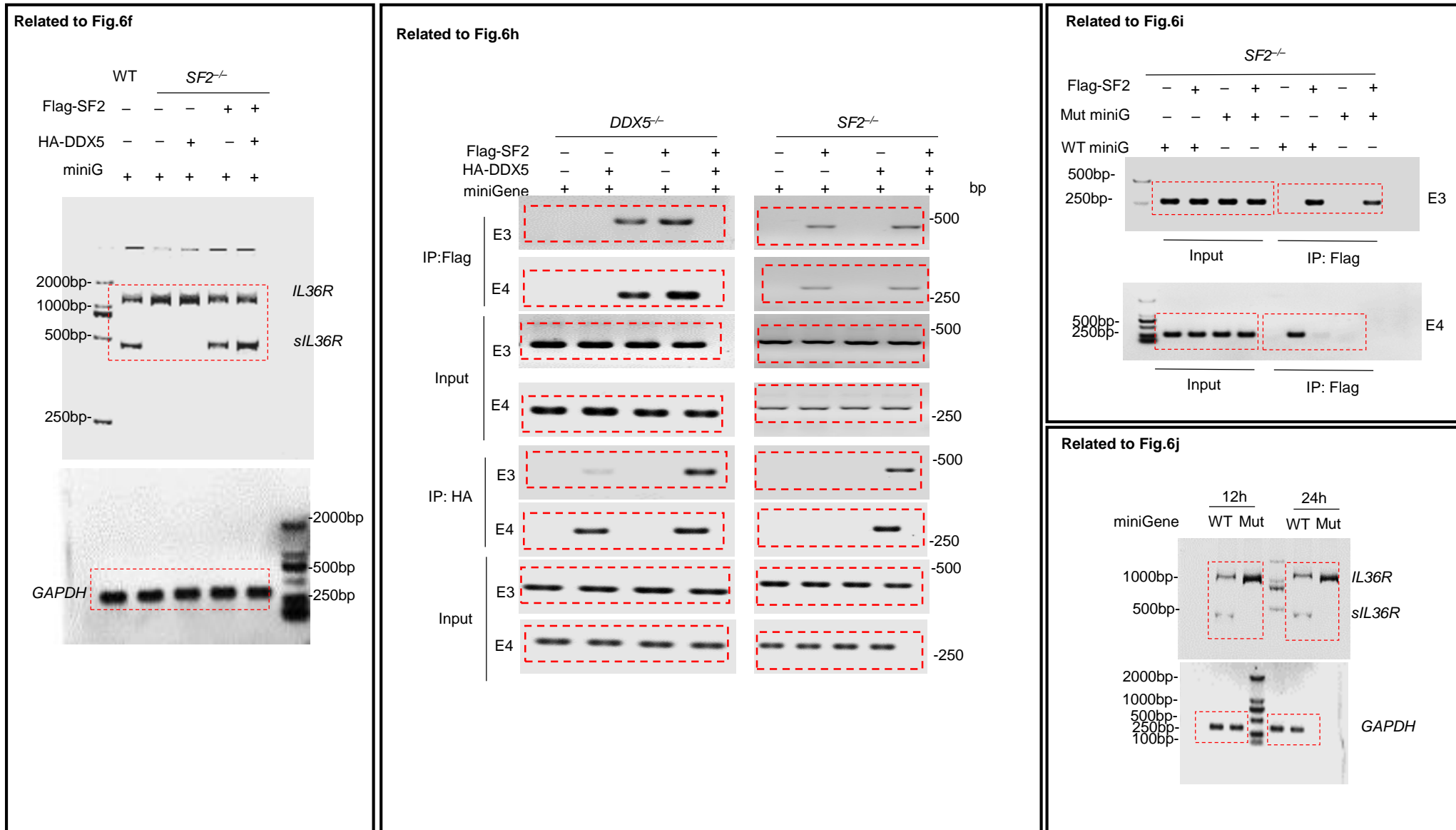

Supplement: Source Data Fig. 6 — Unprocessed immunoblots and gels. [file 41590_2022_1339_MOESM13_ESM.pdf]

Source Data Figure 7 – Unprocessed Immunoblots

Related to Fig.7a

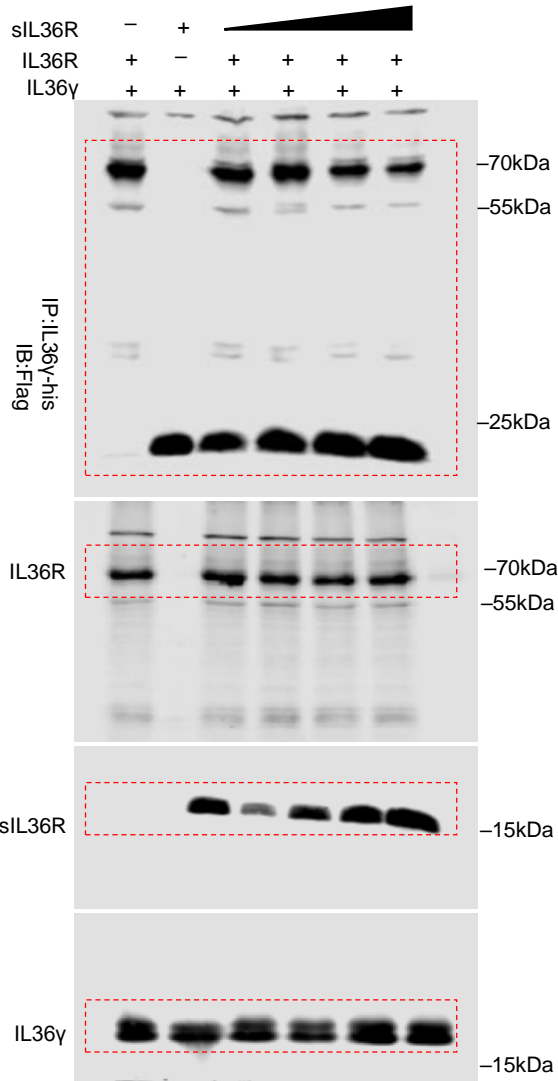

Related to Fig.7b

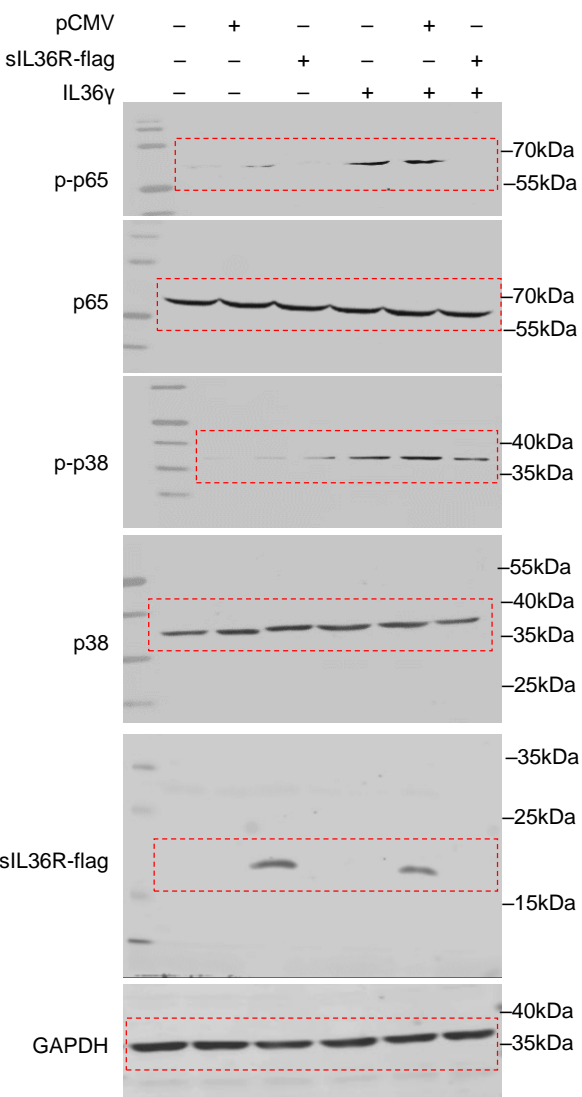

Supplement: Source Data Fig. 7 — Unprocessed immunoblots. [file 41590_2022_1339_MOESM15_ESM.pdf]

Source Data Extended Data Figure 3 – Unprocessed Immunoblots

Related to Extended Data Fig.3b

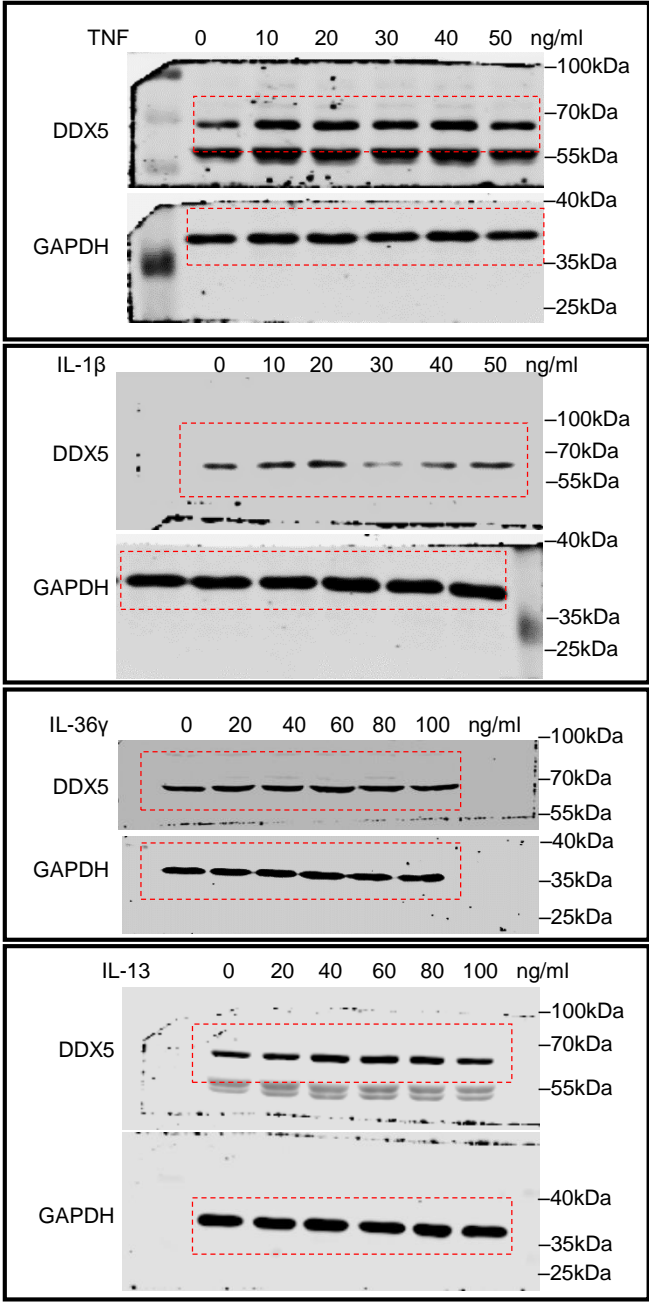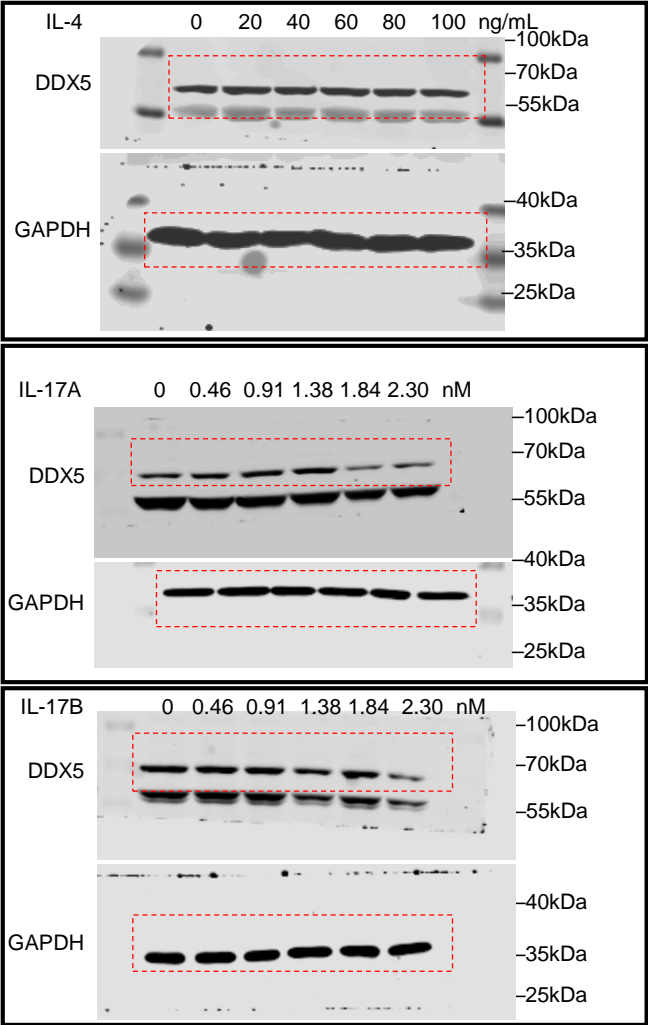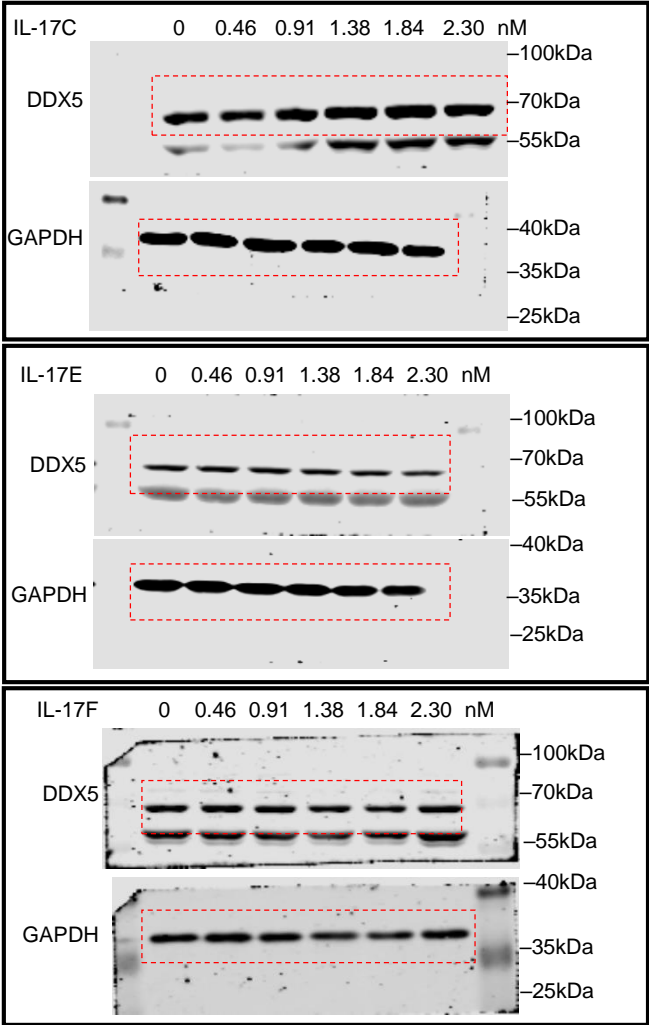

Supplement: Source Data Extended Data Fig. 3 — Unprocessed immunoblots. [file 41590_2022_1339_MOESM20_ESM.pdf]

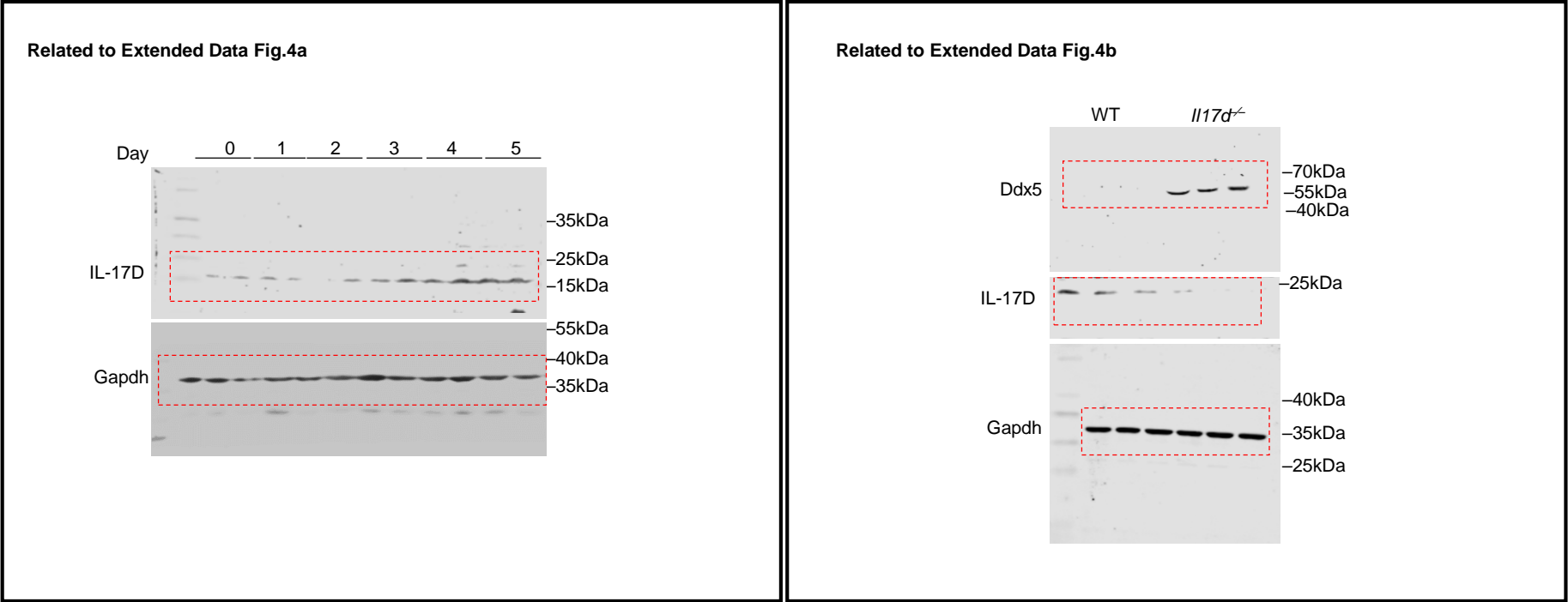

Supplement: Source Data Extended Data Fig. 4 — Unprocessed immunoblots. [file 41590_2022_1339_MOESM22_ESM.pdf]

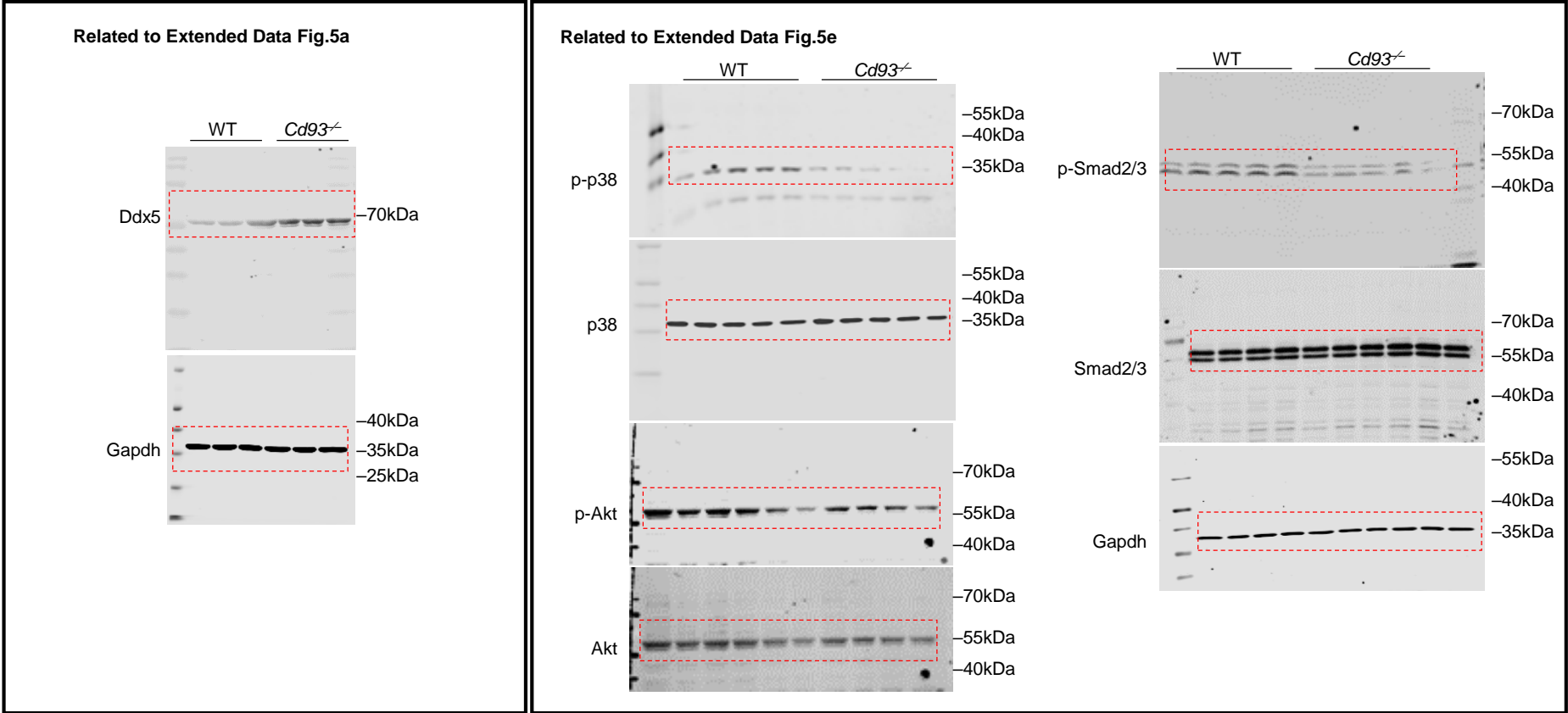

Supplement: Source Data Extended Data Fig. 5 — Unprocessed immunoblots. [file 41590_2022_1339_MOESM24_ESM.pdf]

Related to Extended Data Fig.8b

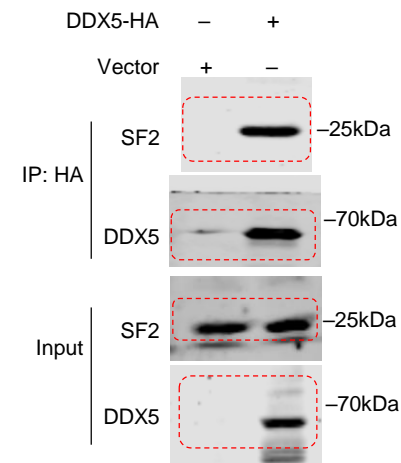

Related to Extended Data Fig.8g

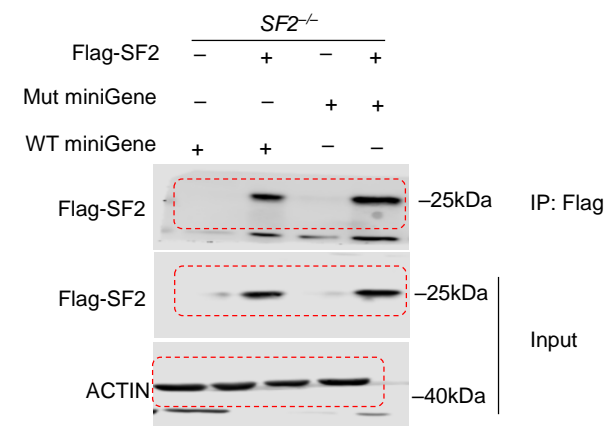

Related to Extended Data Fig.8c

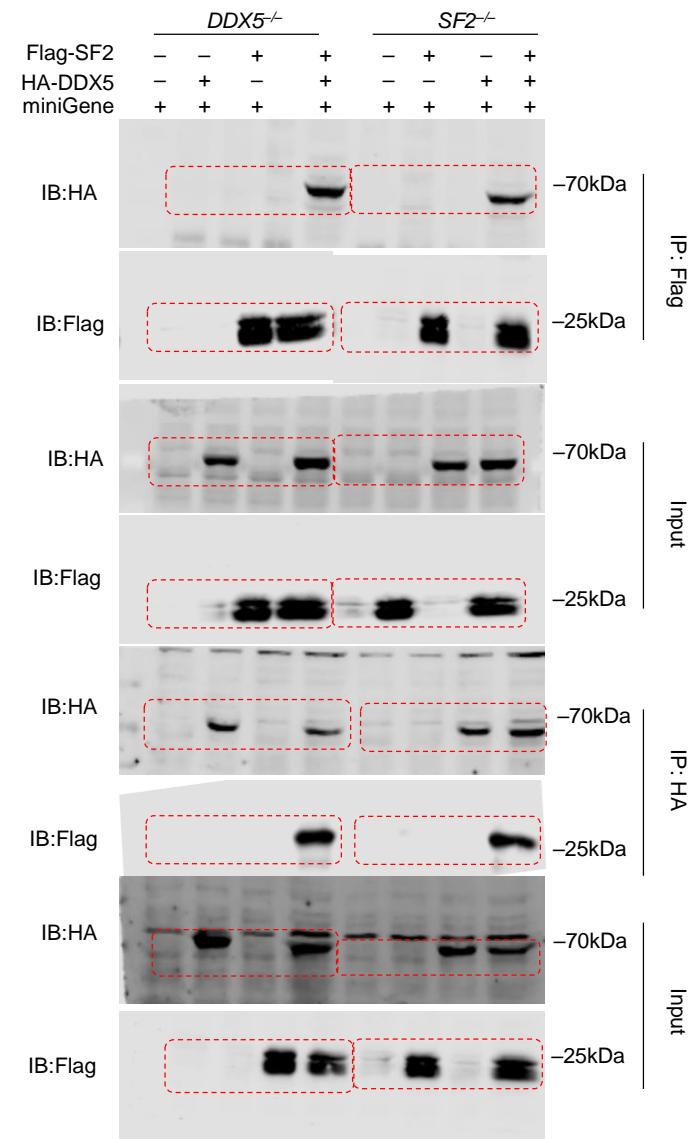

Supplement: Source Data Extended Data Fig. 8 — Unprocessed immunoblots. [file 41590_2022_1339_MOESM30_ESM.pdf]
