# Supplementary material for: IL-17D-induced inhibition of DDX5 expression in keratinocytes amplifies IL-36R-mediated skin inflammation
Source: Nat Immunol. 2022 Oct 21;23(11):1577–87. doi: 10.1038/s41590-022-01339-3 (PMC9663298; doi:10.1038/s41590-022-01339-3)

Source Data Figure 4 – Unprocessed Immunoblots

Related to Fig.4g

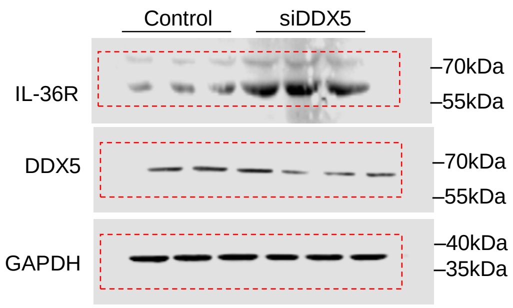

Related to Fig.4h

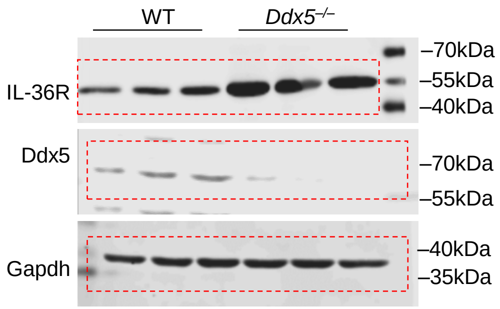

Related to Fig.4j

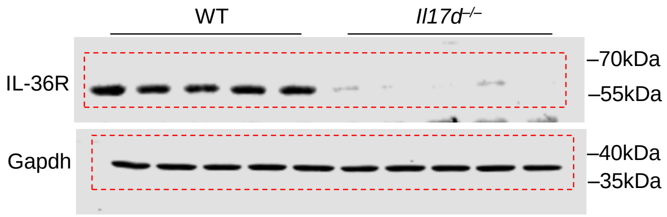

Related to Fig.4i

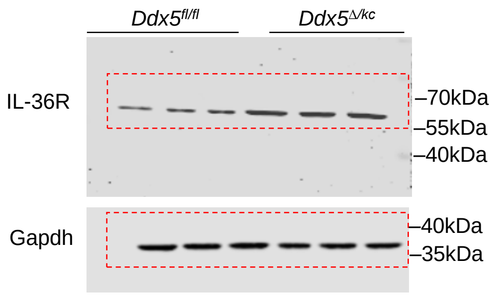

Related to Fig.4l

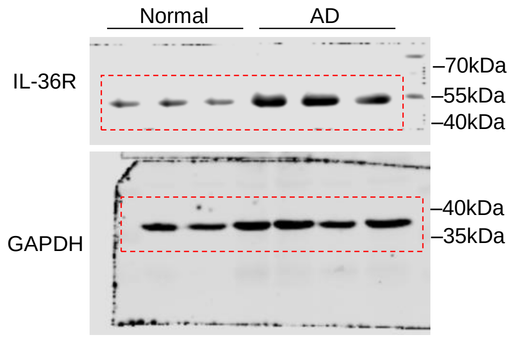

Related to Fig.4k

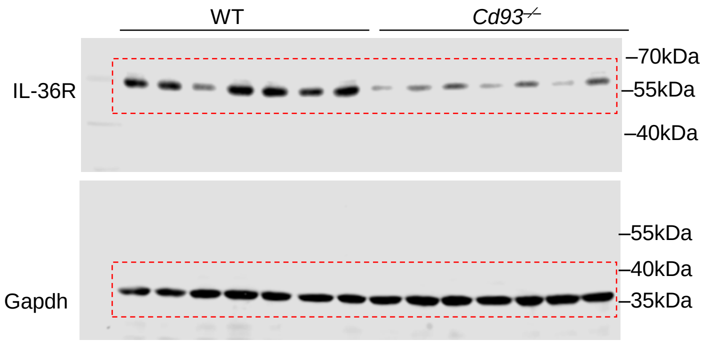

Supplement: Source Data Fig. 4 — Unprocessed immunoblots. [file 41590_2022_1339_MOESM11_ESM.pdf]
