# Supplementary material for: IL-17D-induced inhibition of DDX5 expression in keratinocytes amplifies IL-36R-mediated skin inflammation
Source: Nat Immunol. 2022 Oct 21;23(11):1577–87. doi: 10.1038/s41590-022-01339-3 (PMC9663298; doi:10.1038/s41590-022-01339-3)

Source Data Figure 5 – Unprocessed Gels

Related to Fig.5a

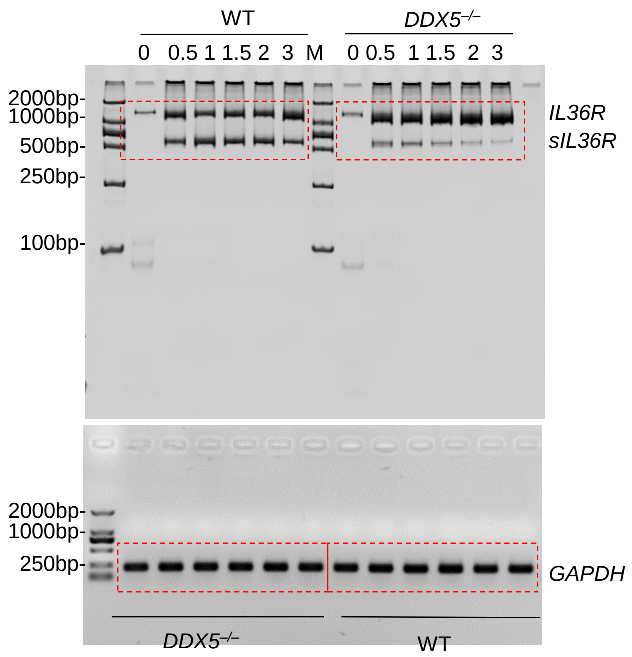

Related to Fig.5b

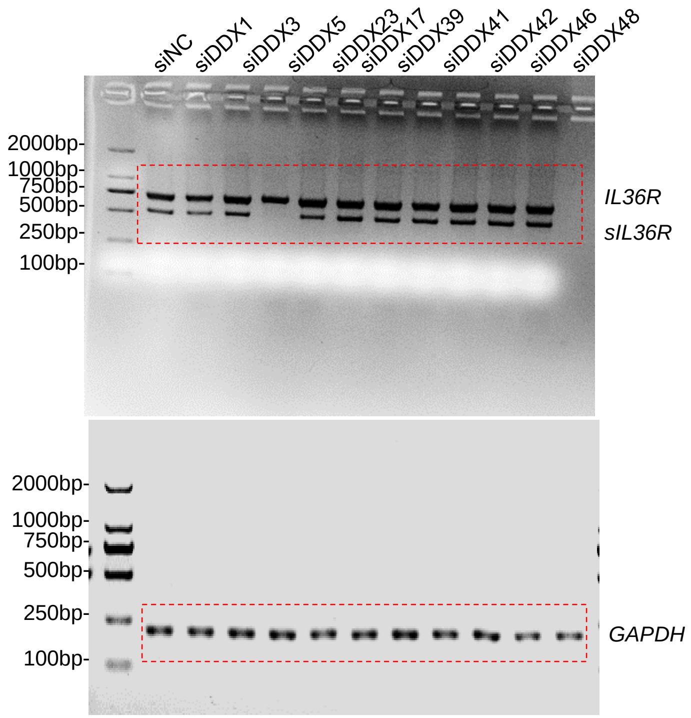

Source Data Figure 5 – Unprocessed Immunoblots

Related to Fig.5c

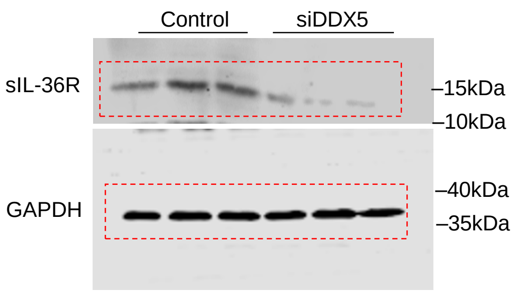

Related to Fig.5d

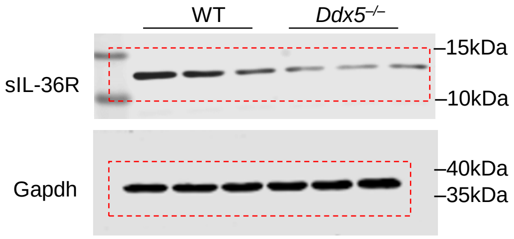

Related to Fig.5e

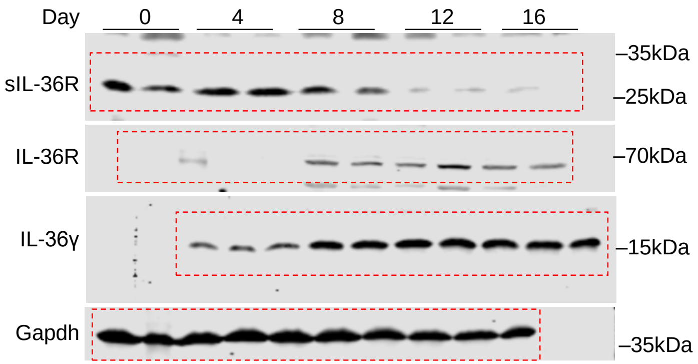

Related to Fig.5f

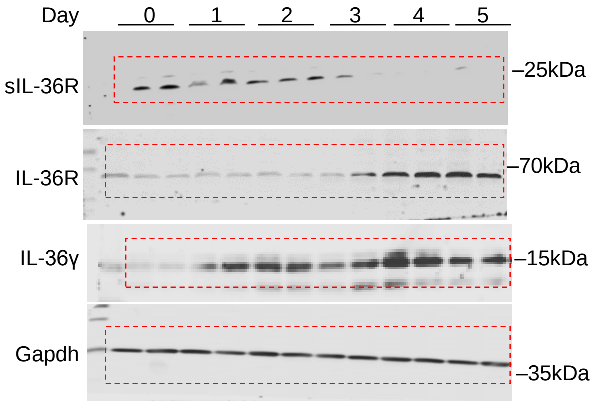

Related to Fig.5g

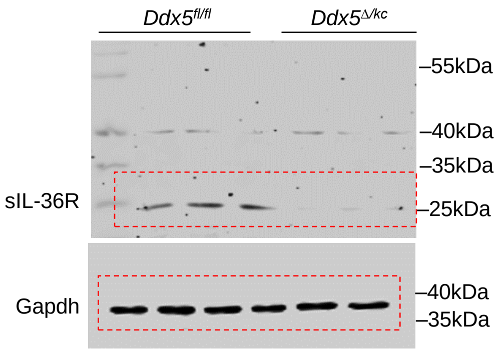

Related to Fig.5h

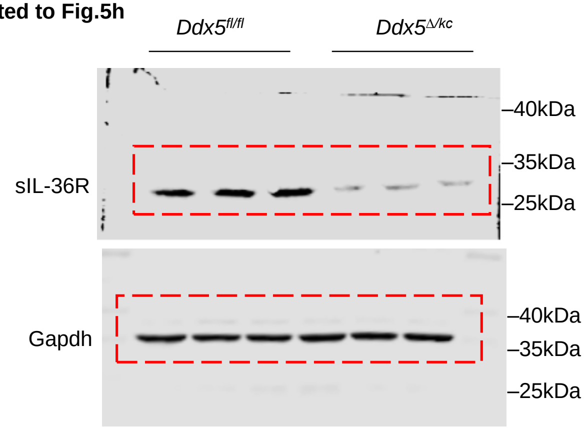

Source Data Figure 5 – Unprocessed Immunoblots

Related to Fig.5i

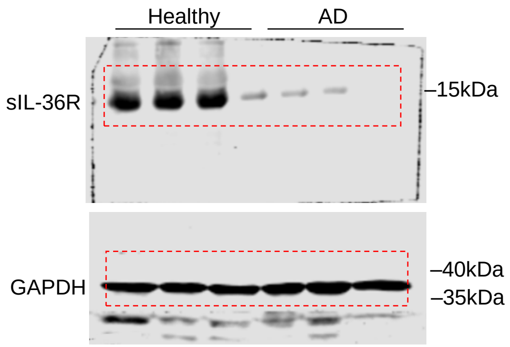

Related to Fig.5j

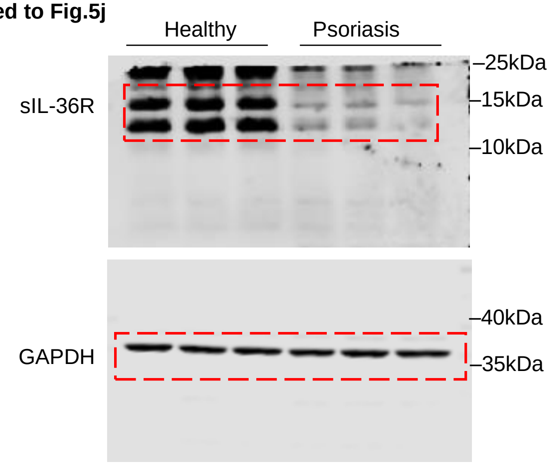

Related to Fig.5k

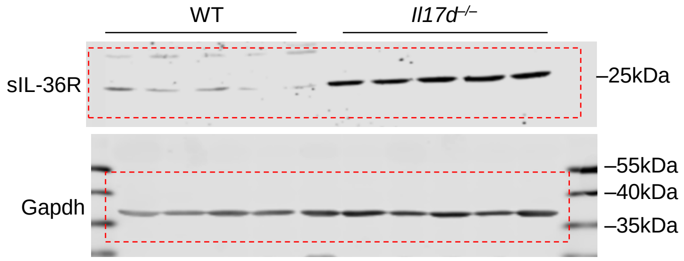

Related to Fig.5l

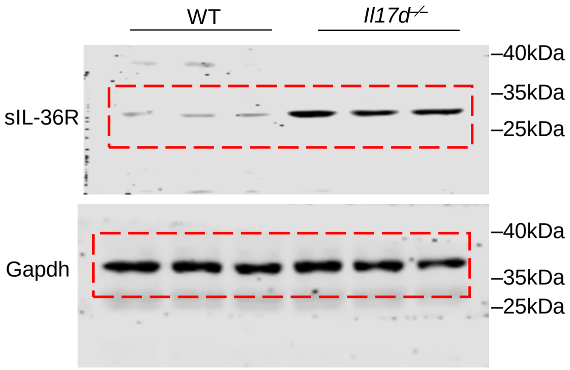

Related to Fig.5m

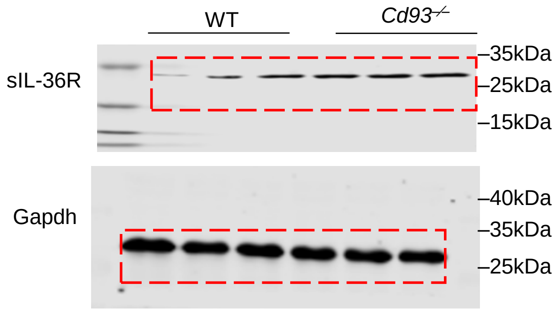

Related to Fig.5n

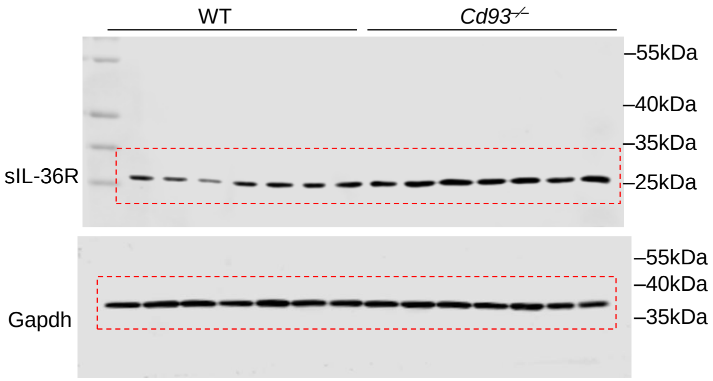

Supplement: Source Data Fig. 5 — Unprocessed immunoblots and gels. [file 41590_2022_1339_MOESM12_ESM.pdf]
