# Supplementary material for: IL-17D-induced inhibition of DDX5 expression in keratinocytes amplifies IL-36R-mediated skin inflammation
Source: Nat Immunol. 2022 Oct 21;23(11):1577–87. doi: 10.1038/s41590-022-01339-3 (PMC9663298; doi:10.1038/s41590-022-01339-3)

Source Data Extended Data Figure 6 – Unprocessed Immunoblots

Related to Extended Data Fig.6j

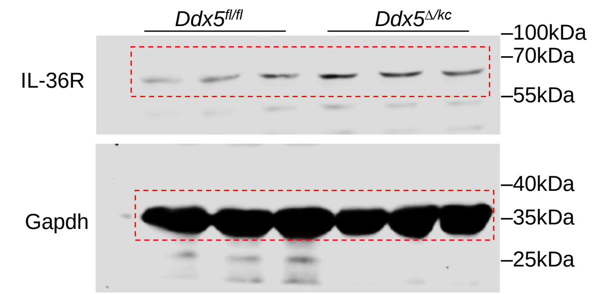

Related to Extended Data Fig.6l

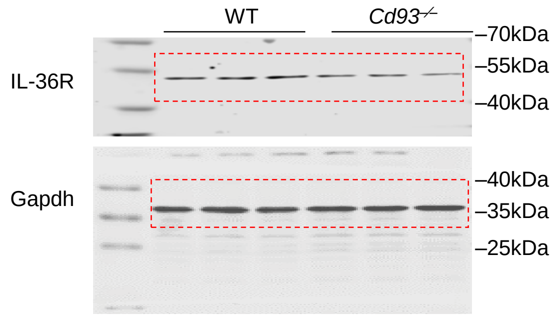

Related to Extended Data Fig.6k

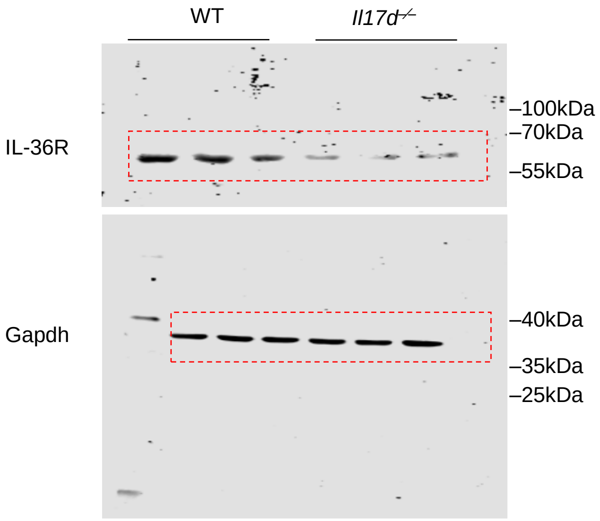

Related to Extended Data Fig.6m

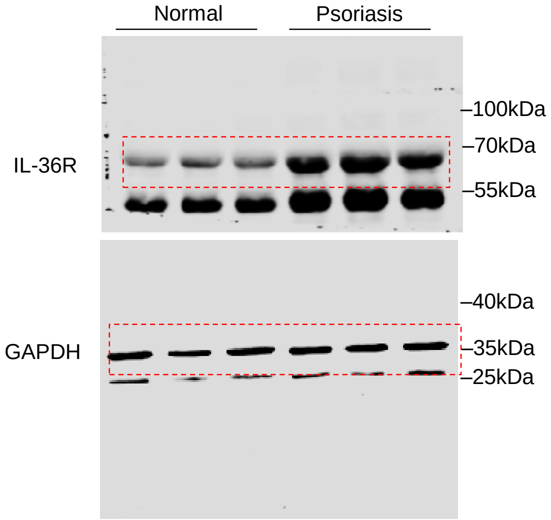

Supplement: Source Data Extended Data Fig. 6 — Unprocessed immunoblots. [file 41590_2022_1339_MOESM26_ESM.pdf]
