# Supplementary material for: IL-17D-induced inhibition of DDX5 expression in keratinocytes amplifies IL-36R-mediated skin inflammation
Source: Nat Immunol. 2022 Oct 21;23(11):1577–87. doi: 10.1038/s41590-022-01339-3 (PMC9663298; doi:10.1038/s41590-022-01339-3)

Source Data Extended Data Figure 7 – Unprocessed Gels and Immunoblots

Unprocessed Gels

Related to Extended Data Fig.7b

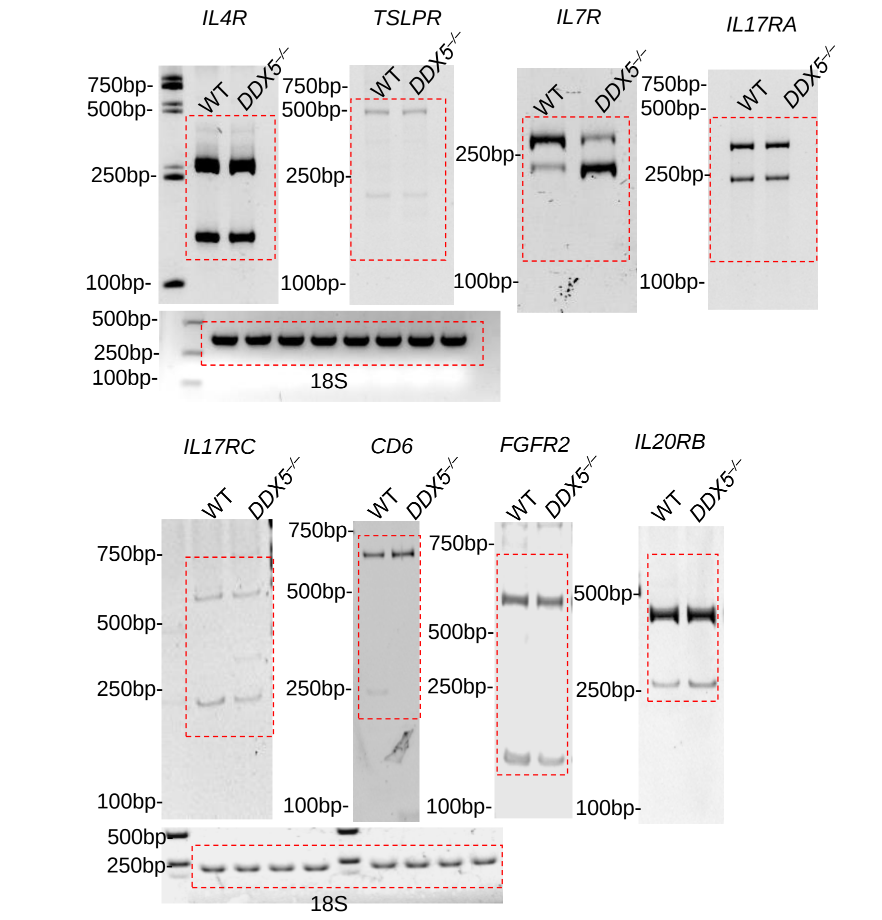

Related to Extended Data Fig.7c

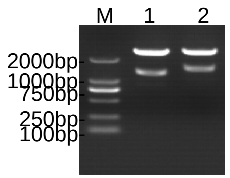

Related to Extended Data Fig.7d

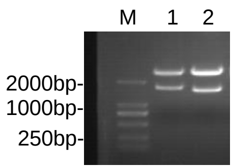

Related to Extended Data Fig.7i

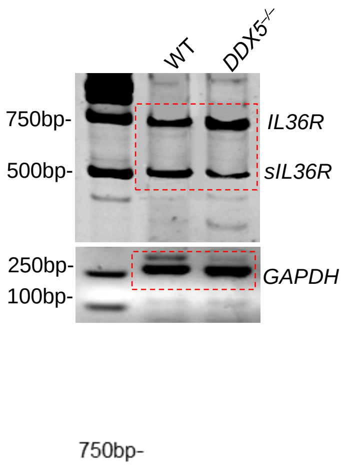

Related to Extended Data Fig.7k

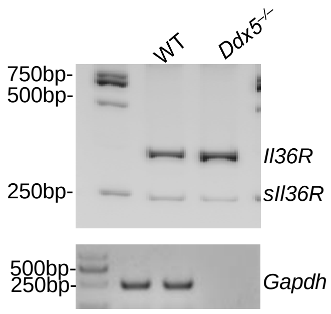

Unprocessed Immunoblots

Related to Extended Data Fig.7g

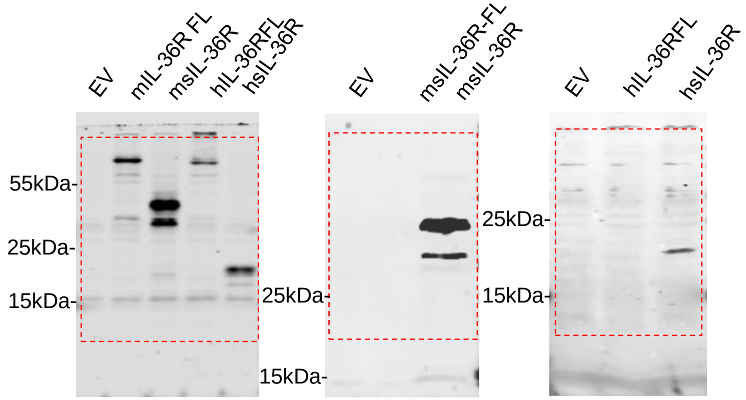

Related to Extended Data Fig.7h

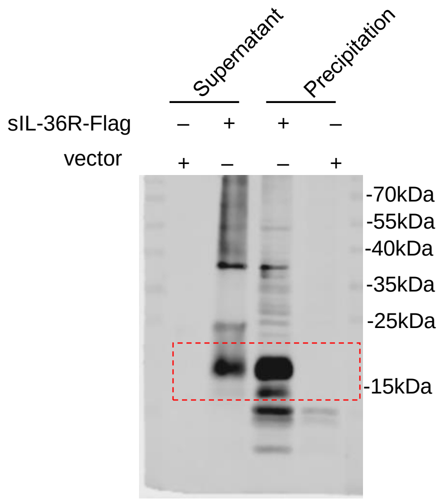

Supplement: Source Data Extended Data Fig. 7 — Unprocessed immunoblots and gels. [file 41590_2022_1339_MOESM28_ESM.pdf]
