# Supplementary material for: Author Correction: IL-17D-induced inhibition of DDX5 expression in keratinocytes amplifies IL-36R-mediated skin inflammation
Source: Nat Immunol. 2026 Aug 17;27(10):2172. doi: 10.1038/s41590-026-02636-x (PMC13630555; doi:10.1038/s41590-026-02636-x)

nature immunology

Supplementary information

Author Correction: IL-17D-induced inhibition of DDX5 expression in keratinocytes amplifies IL-36R-mediated skin inflammation

In the format provided by the authors and unedited

Original and Corrected Fig. 2b

Original

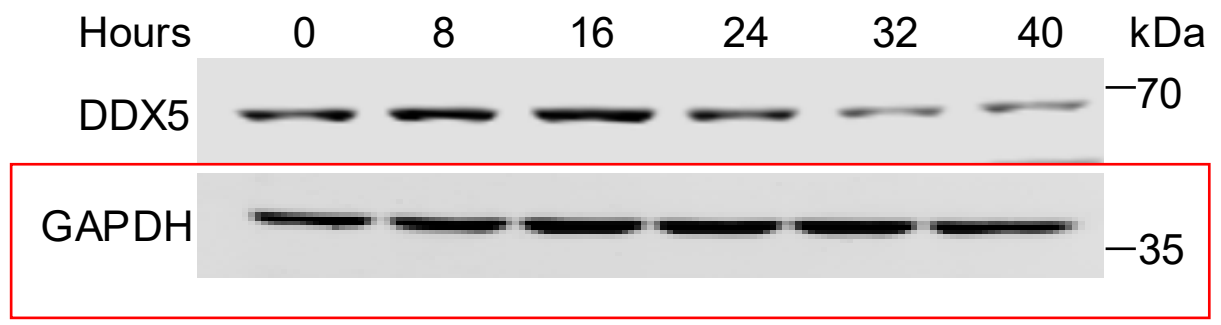

Corrected

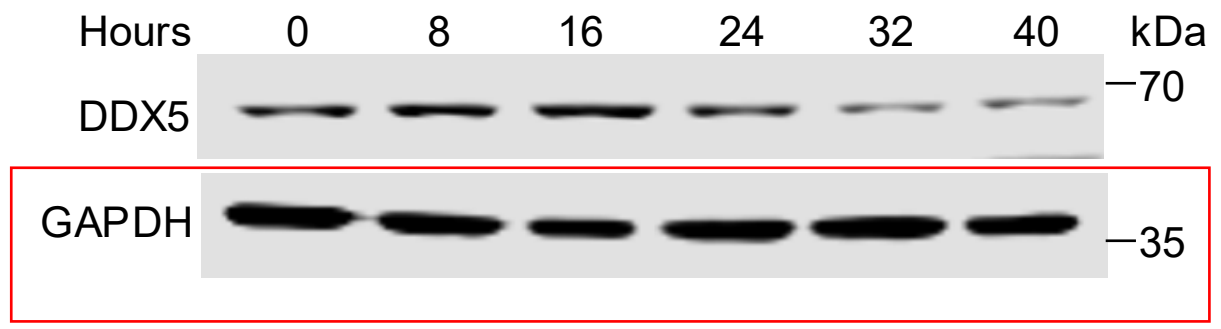

Original and Corrected Fig. 2h

Original

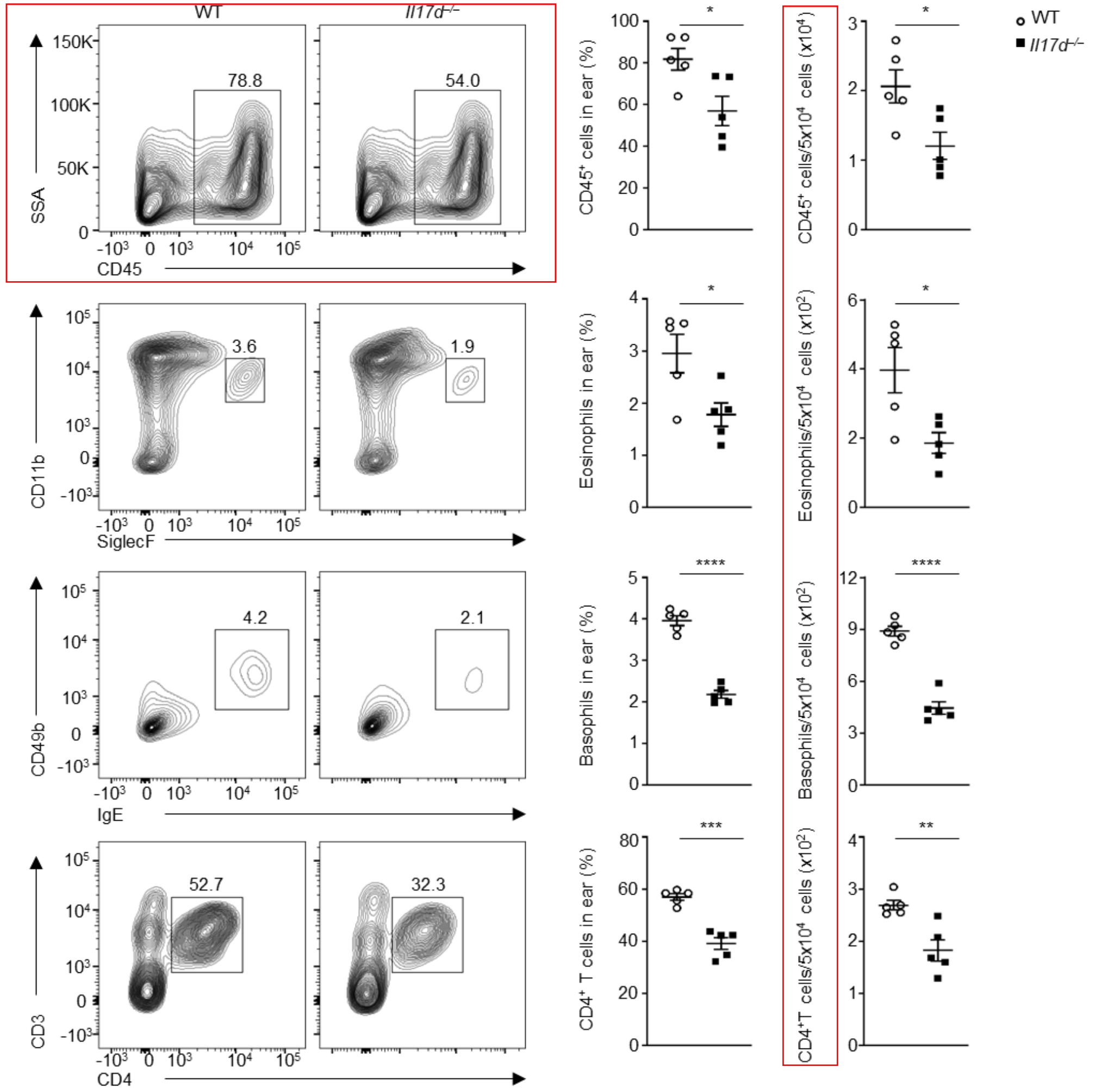

Corrected

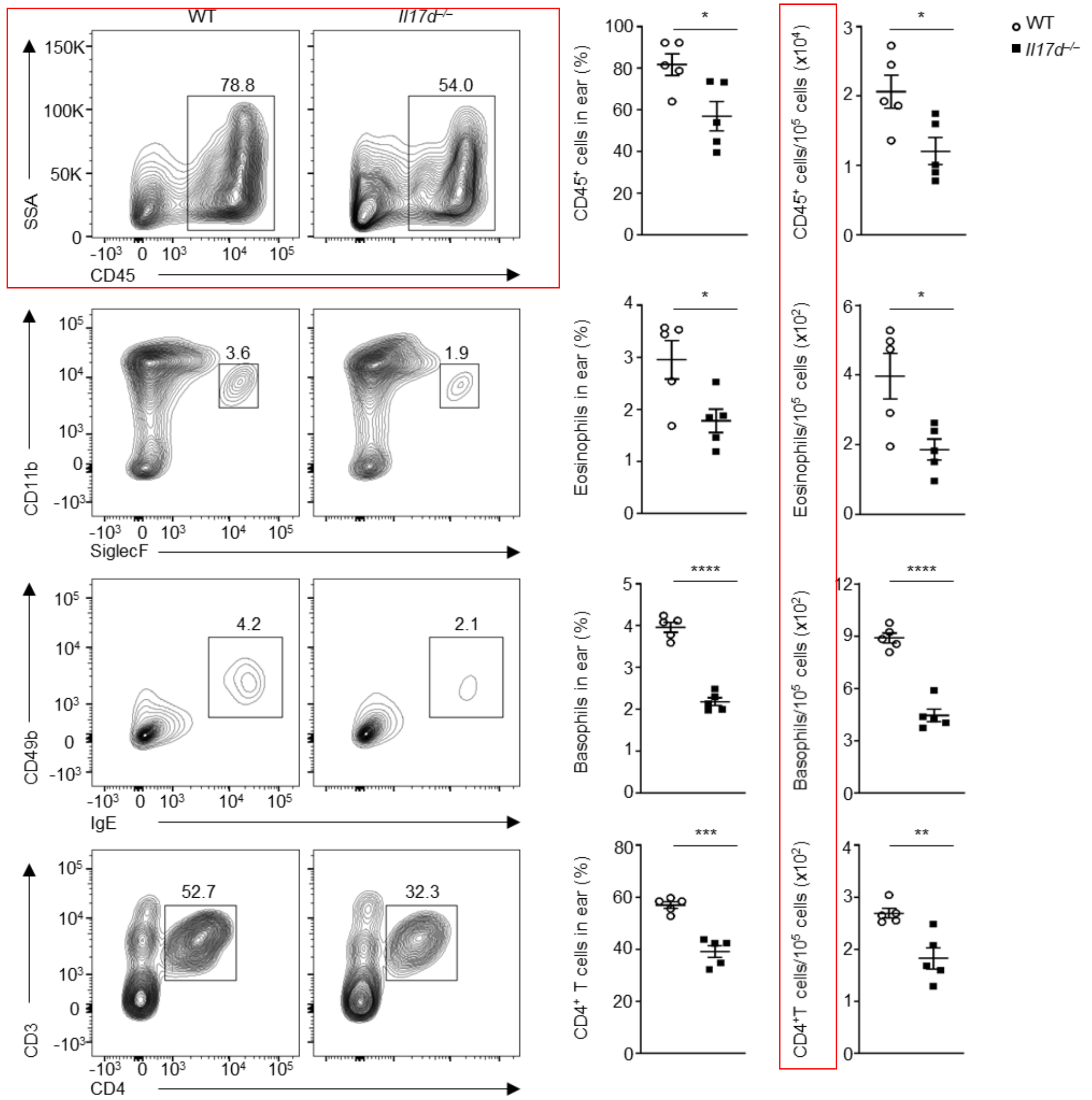

Original and Corrected Fig. 3h

Original

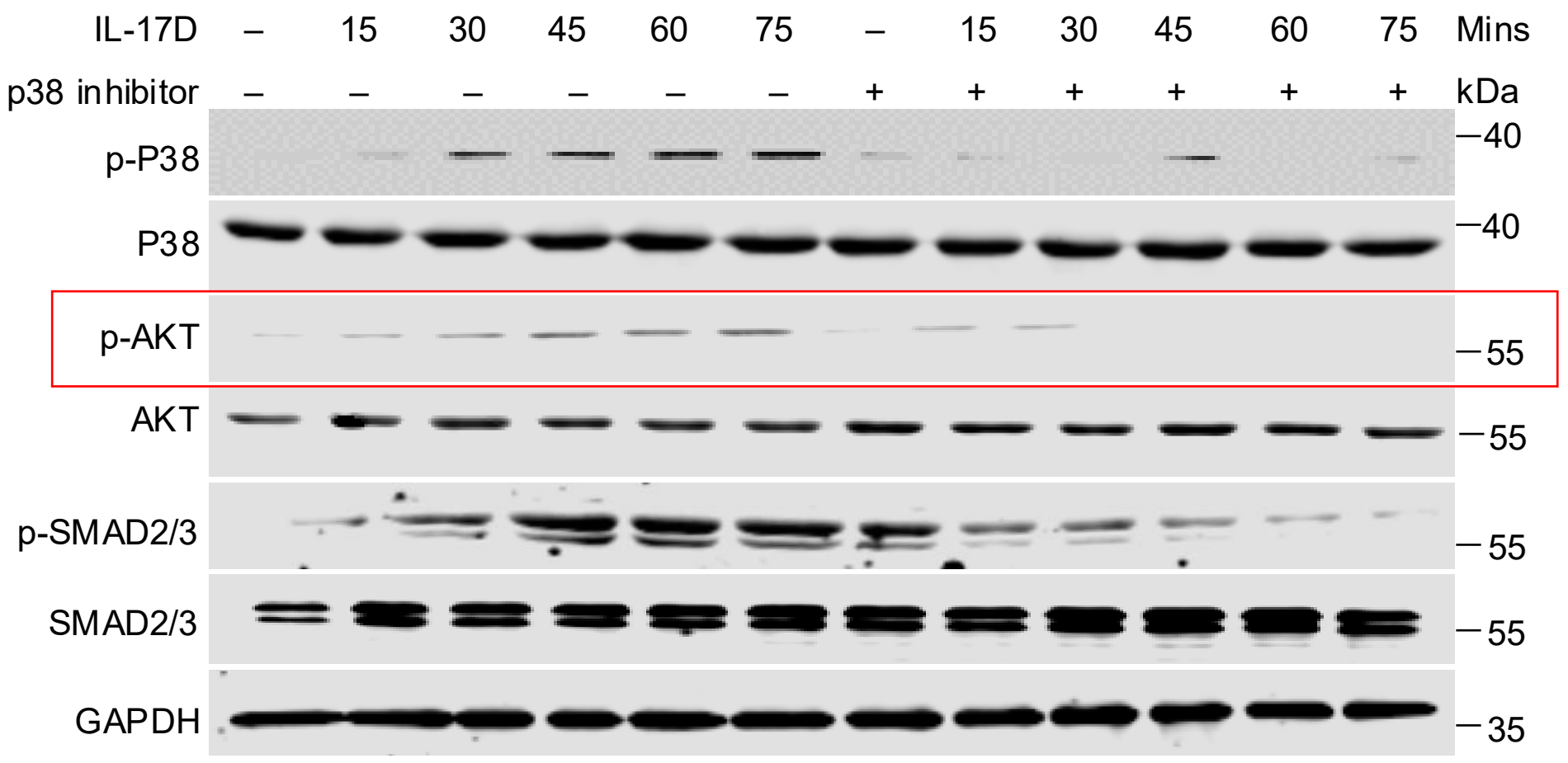

Corrected

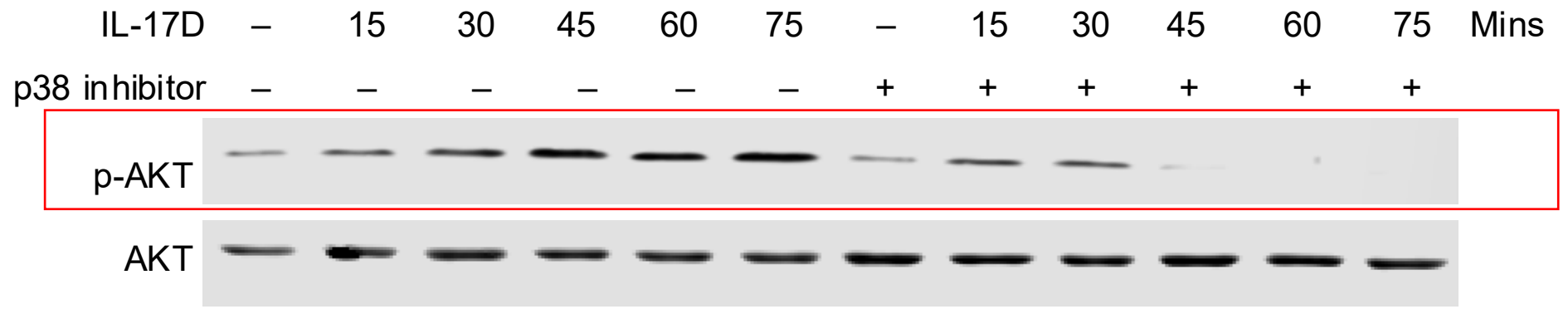

Original and Corrected Fig. 3i

Original

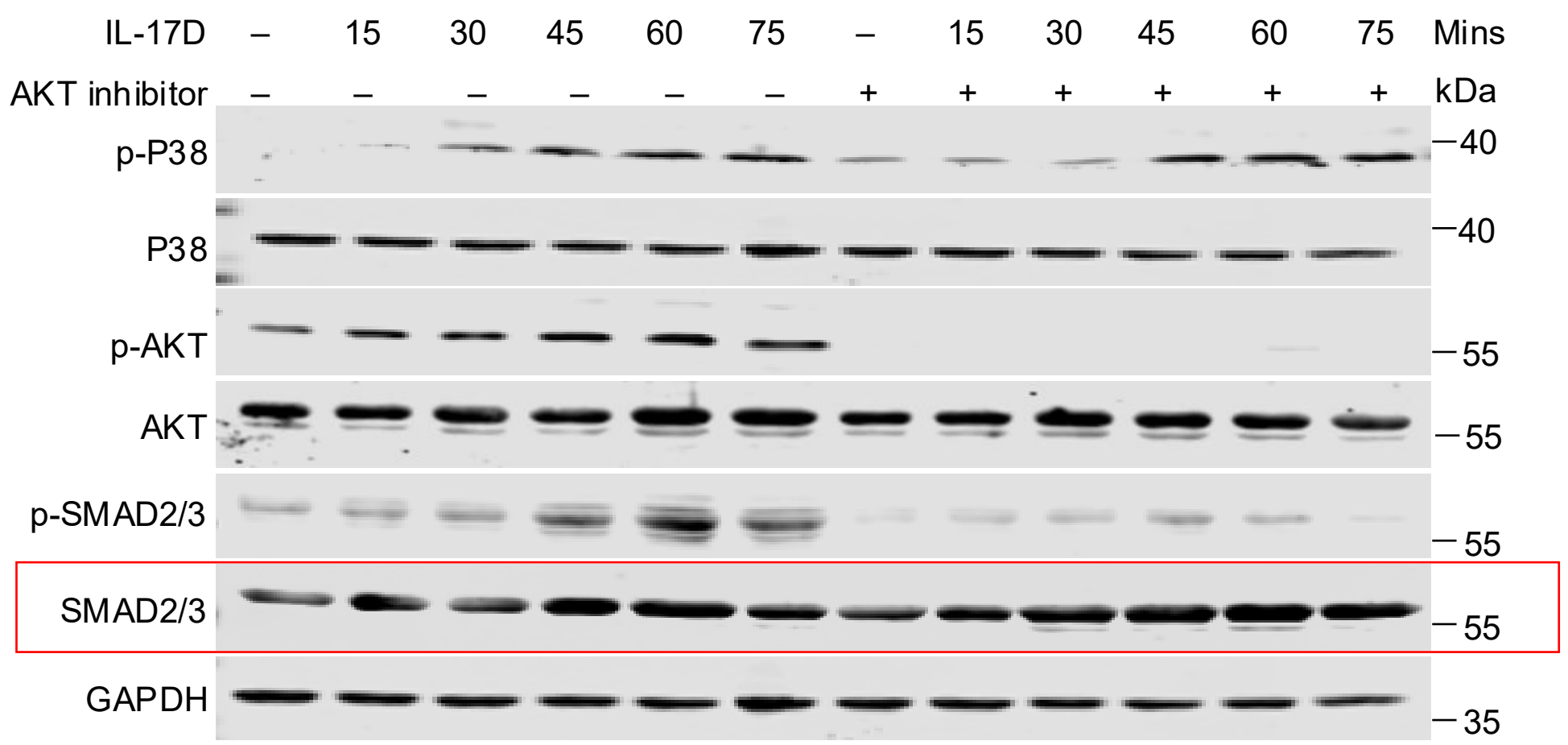

Corrected

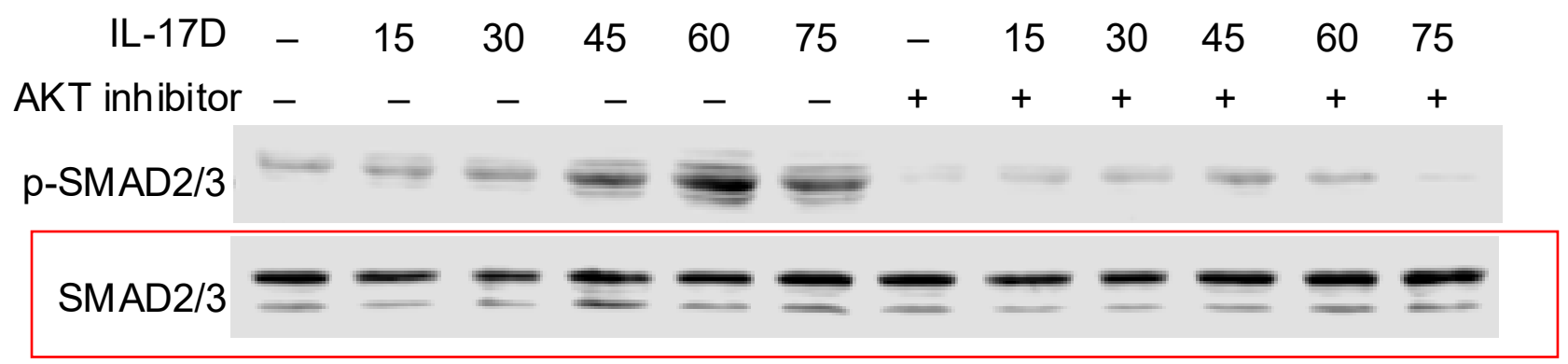

Original and Corrected Extended Data Fig. 4a

Original

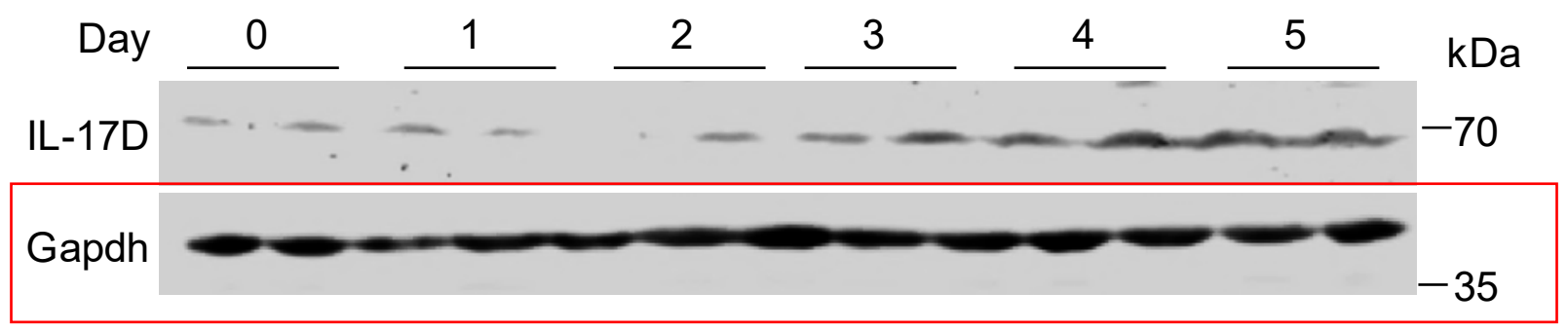

Corrected

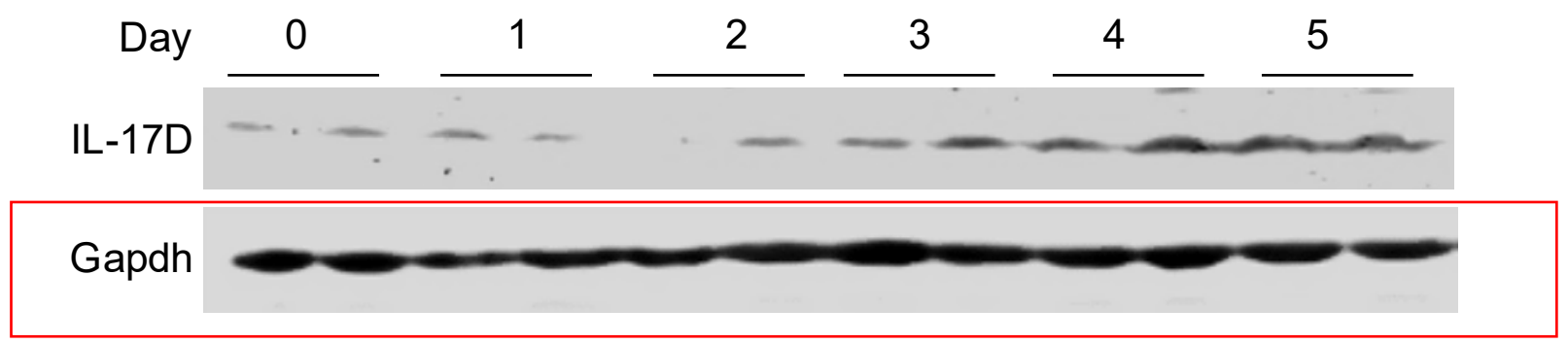

Supplement: Supplementary file 1 [file 41590_2026_2636_MOESM1_ESM.pdf]
